# An Overview of Spike Surface Glycoprotein in Severe Acute Respiratory Syndrome–Coronavirus

**DOI:** 10.3389/fmolb.2021.637550

**Published:** 2021-03-16

**Authors:** Muthu Kumaradoss Kathiravan, Srimathi Radhakrishnan, Vigneshwaran Namasivayam, Senthilkumar Palaniappan

**Affiliations:** ^1^ Department of Pharmaceutical Chemistry, SRM College of Pharmacy, SRMIST, Tamil Nadu, India; ^2^ Dr. APJ Abdul Kalam Research Lab, SRM College of Pharmacy, SRMIST, Tamil Nadu, India; ^3^ Pharmaceutical Institute, University of Bonn, Bonn, Germany; ^4^ Faculty of Pharmacy, Karpagam Academy of Higher Education, Tamil Nadu, India

**Keywords:** SARS-CoV-2, coronavirus, spike (S) glycoprotein, entry inhibitors, corona

## Abstract

The novel coronavirus originated in December 2019 in Hubei, China. This contagious disease named as COVID-19 resulted in a massive expansion within 6 months by spreading to more than 213 countries. Despite the availability of antiviral drugs for the treatment of various viral infections, it was concluded by the WHO that there is no medicine to treat novel CoV, SARS-CoV-2. It has been confirmed that SARS-COV-2 is the most highly virulent human coronavirus and occupies the third position following SARS and MERS with the highest mortality rate. The genetic assembly of SARS-CoV-2 is segmented into structural and non-structural proteins, of which two-thirds of the viral genome encodes non-structural proteins and the remaining genome encodes structural proteins. The most predominant structural proteins that make up SARS-CoV-2 include spike surface glycoproteins (S), membrane proteins (M), envelope proteins (E), and nucleocapsid proteins (N). This review will focus on one of the four major structural proteins in the CoV assembly, the spike, which is involved in host cell recognition and the fusion process. The monomer disintegrates into S1 and S2 subunits with the S1 domain necessitating binding of the virus to its host cell receptor and the S2 domain mediating the viral fusion. On viral infection by the host, the S protein is further cleaved by the protease enzyme to two major subdomains S1/S2. Spike is proven to be an interesting target for developing vaccines and in particular, the RBD-single chain dimer has shown initial success. The availability of small molecules and peptidic inhibitors for host cell receptors is briefly discussed. The development of new molecules and therapeutic druggable targets for SARS-CoV-2 is of global importance. Attacking the virus employing multiple targets and strategies is the best way to inhibit the virus. This article will appeal to researchers in understanding the structural and biological aspects of the S protein in the field of drug design and discovery.

## Introduction

**FIGURE 1:**
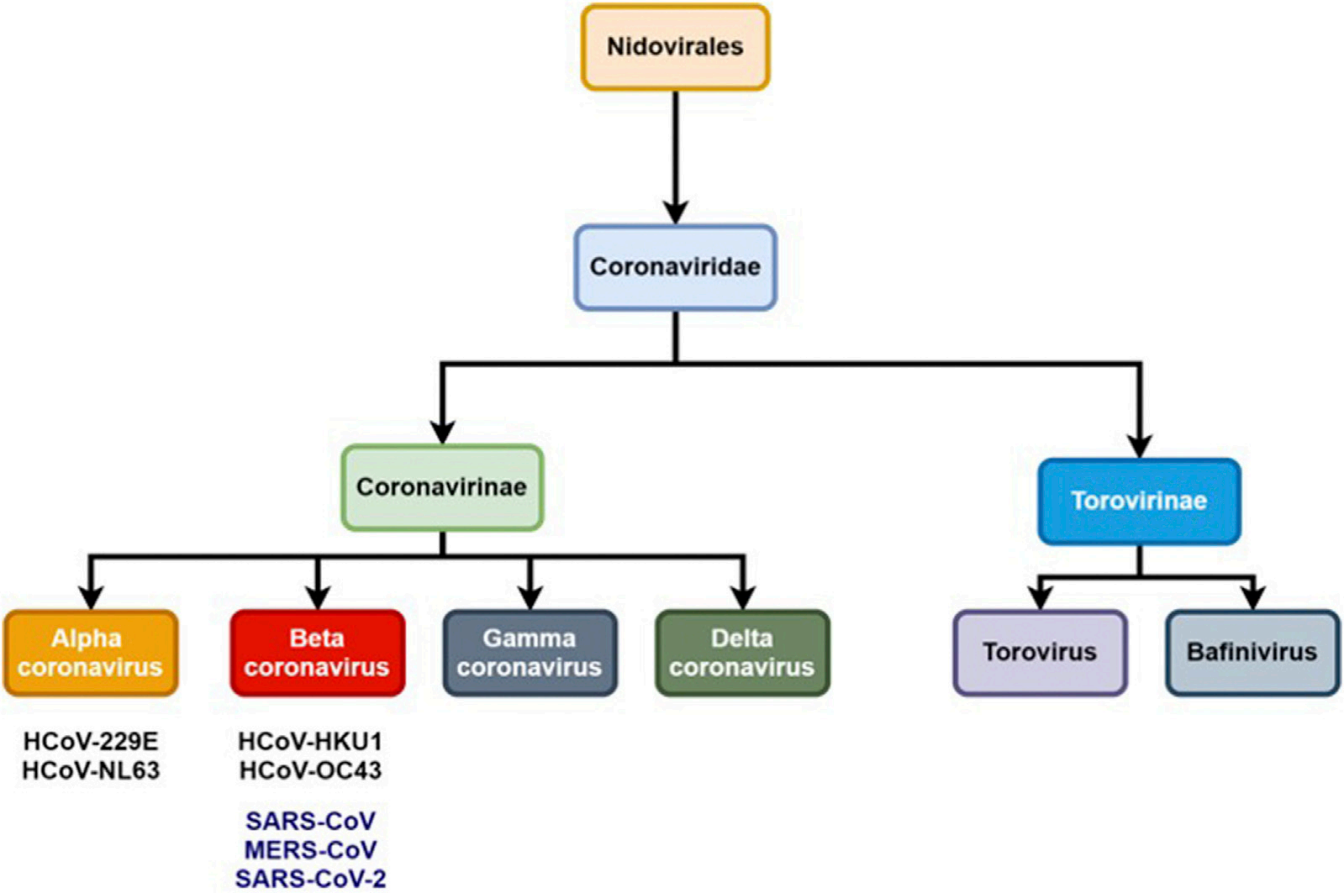
Schematic representation of Coronaviridae taxonomy.

Coronaviruses (CoVs) are pathogens from the Coronaviridae family and have an impact on human and animal health; in particular their respiratory and gastrointestinal tract system whose symptoms range from mild to lethal (Ghosh et al., 2020). The International Committee on Taxonomy of Viruses (ICTV) classifies coronaviruses into the Coronaviridae family and Nidovirales order which is further subdivided into two subfamilies: Torovirinae and Coronavirinae (Figure 1) (Pal et al., 2020).

The CoVs are large and enveloped positive-strand RNA viruses, and can be further subdivided into α-, β-, γ-, and δ- CoVs. Among the four subtypes, α- and β- CoVs are known to infect humans. Until now six human-CoVs (HCoV 229E, NL63, OC43, HKU1, SARS, and MERS) have been reported globally. Table 1 illustrates the classification of coronavirus, variants, and their host organism. Among these six human-CoVs, SARS- and MERS- CoVs are extremely pathogenic and the transmission within humans generally occurs via close contact through the inhalation of respiratory droplets or sneezing, similar to influenza and other respiratory pathogens (Pillaiyar et al., 2016; Ghosh et al., 2020). The remaining four CoVs cause mild respiratory infections leading to the common cold. At the end of 2002, the outbreak of SARS in Guangdong province in China registered 8098 cases with 774 deaths. Almost a decade passed since the outbreak of SARS-CoV, the subsequent zoonotic coronavirus MERS-CoVs emerged in Saudi Arabia with 2494 cases and 858 deaths (Source: WHO). At the end of 2019, another new strain of coronavirus 2019-nCoV was found among people reported for the recent ongoing pneumonia outbreak in the city of Wuhan in China (Huang C. et al., 2020). Until now (as of December 2, 2020, WHO) the 2019-nCoV had spread rapidly in over 220 countries and registered over 63,360,234 reported cases and 1,475,825 deaths (https://www.who.int/emergencies/diseases/novel-coronavirus-2019).

**TABLE 1:** Classification of different types of CoVs with their variants name, year of discovery, and host organism.

Type of CoV	Coronaviruses	Discovery	Natural host(s)
α-coronaviruses	HCoV-229E	1966	Bats
	HCoV-NL63	2004	Palm civets, bats
β-coronaviruses	HCoV-OC43	1967	Cattle
	SARS-CoV-1	2003	Palm civets
	HCoV-HKU1	2005	Mice
	MERS-CoV	2012	Bats, camels
	SARS-CoV-2	2019	Bats
Non-human	BCoV	1890	Cattle
	TGEV	1946	Pigs
	MHV	1949	Mice
	FIPV	1963	Cats
	CCoV	1971	Dogs
	PEDV	2013	Pigs

## Coronavirus Replication Cycle

**FIGURE 2:**
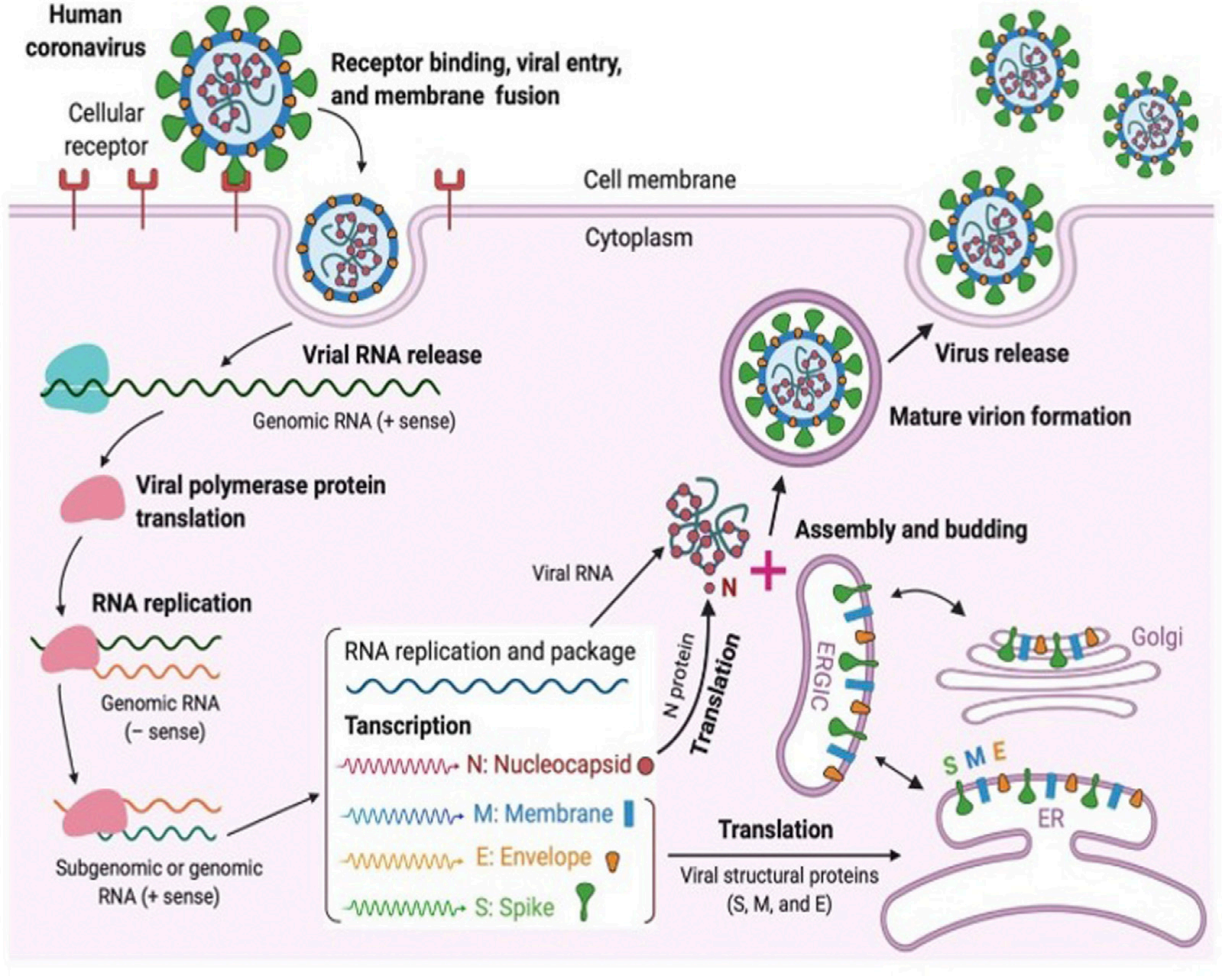
Coronavirus replication cycle [(Jiang et al., 2020) Copyright ©2020 Elsevier Inc., based on the reuse provisions of Elsevier’s COVID-19 Resource Center].

CoVs contain a non-segmented single-stranded RNA featuring the largest viral RNA genomes reported so far and ranging from approximately 26–32 kilobase (kb) genomes. CoVs are lipid enveloped and spherical in shape with a size of approximately 100–120 nM (Ghosh et al., 2020). SAR-CoV-2 belongs to the beta-coronavirus class comprising of ∼30 kb in length, and the replication cycle is shown in Figure 2.

Similar to other CoV neighbors, SARS-CoV-2 utilizes the host cell machinery for replication which involves various viral structural and non-structural proteins. Coronavirus particles consist of four main structural proteins namely the spike (S), membrane (M), envelope (E), and nucleocapsid (N) proteins. Briefly, a fully mature viral particle starts its journey with host cell membrane fusion or the endocytosis process. The binding of the receptor-binding domain (RBD) of the spike (S) protein to the host receptor, such as angiotensin-converting enzyme-2 (ACE2) or dipepitdylpeptidase IV (DDPIV), changes the RBD conformation which leads to the merging of the viral membrane with the host membrane. With the fusion process, the viral genetic material (single-stranded RNA) is injected into the cytoplasm for the host cell ribosome-dependent translation process in which ORF1ab is translated into viral polyproteins (e.g., pp1a, pp1b, etc.). Subsequently, various non-structural proteins, including RNA-dependent RNA polymerase and helicase, are produced from the pp1a and pp1b using the protease enzymes (e.g., PL^pro^ and 3CL^pro^). Non-structural proteins are involved in the viral transcription and replication process. Several copies of original viral RNA synthesized by RNA polymerase are now transcribed into a full-length mRNA negative-strand template for the translation process in which structural proteins are produced in the endoplasmic reticulum. Ultimately, all structural proteins and genomic RNA are compiled to form the virion, which is translocated into Golgi, where the virions are released out of the cell via transport through vesicles. To inhibit the virus progression, several key steps have been identified in the virus life cycle; 1) RBD binding which plays an important role in the viral fusion to host cells, 2) protease enzymes in synthesizing RNA-dependent RNA polymerase, and 3) RNA-dependent RNA polymerase for transcription. Blocking any of these crucial steps might be an attractive target for antiviral development, including drugs and vaccines (Huang Y. et al., 2020).

## Overview of the S Protein of SARS-CoV-2

**FIGURE 3:**
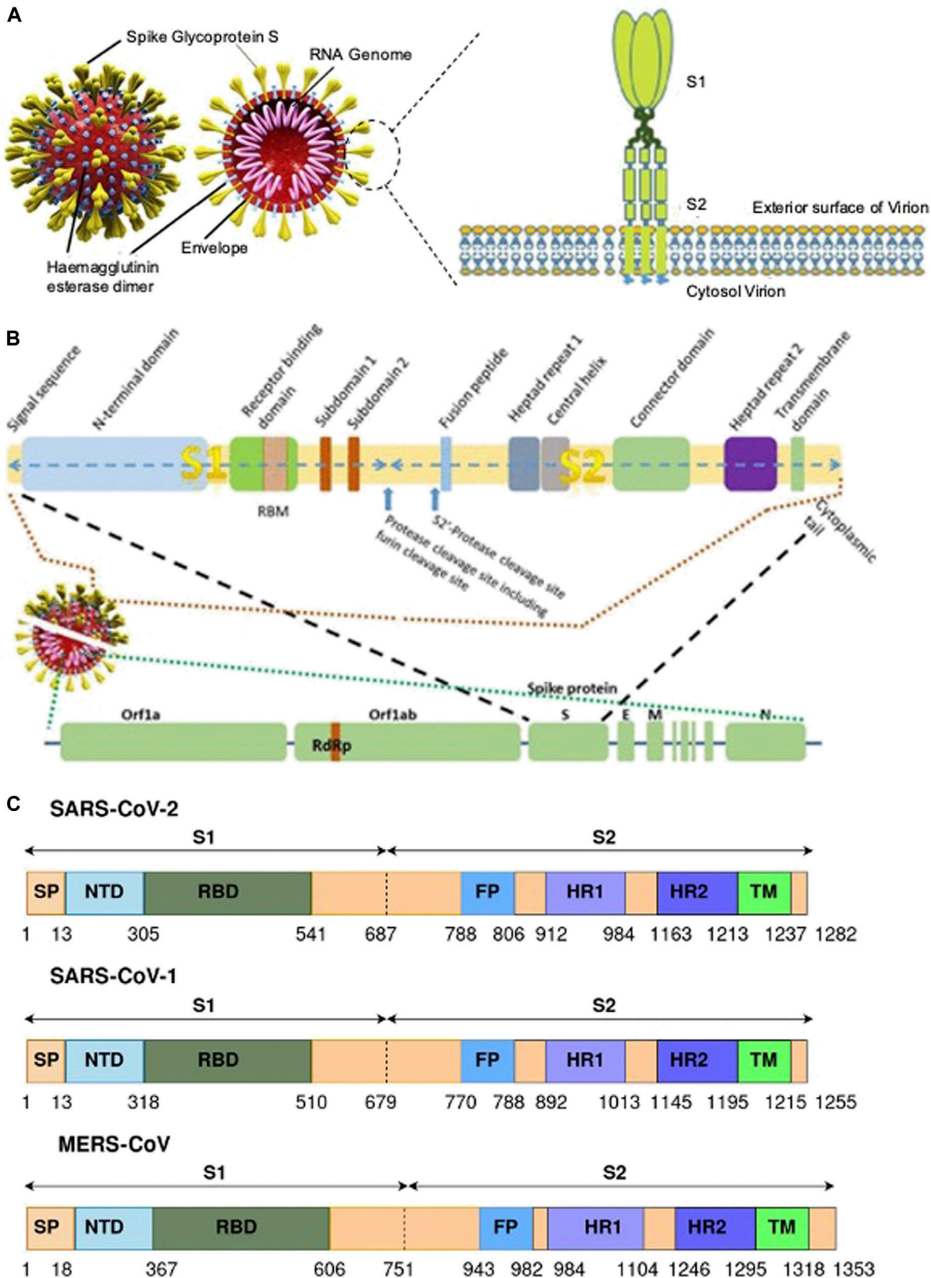
**(A)** A representative of SARS CoV-2 and the S protein with their different binding domains S1 and S2 [(Pillay, 2020) Copyright ©2020 Publisher BMJ]; **(B)** the different region of the S protein of SARS CoV-2 [(Pillay, 2020) Copyright ©2020 Publisher BMJ]; and **(C)** genomic information of the S protein and the different binding domains for SARS-CoV-2, SARS-CoV-1, and MERS-CoV (Bahrami and Ferns, 2020).

The S protein is a homotrimeric transmembrane glycoprotein fused by three monomer units. On the surface of the protein 100 crown-shaped spikes are present of ∼30 kb in length and is a larger part among the four structural proteins M, E, and N (Song et al., 2018). The length of each spike ranges from 20 to 40 nM making it more stretchable to fit into the angiotensin converting enzyme II (ACE2) of the host cell receptor (Zhou et al., 2020). Each monomer (∼180 kDa) of the S protein consists of a total of 1282 amino acids which is divided into two major functional domains S1 and S2 (Bahrami and Ferns, 2020). Thus, the trimer of an S protein contains three S1 and S2 subunits coiled together (Yan et al., 2020). The S1 domain can be segregated into a single peptide (SP), an N-terminal domain (NTD), C-terminal domain (CTD) also called the receptor binding domain (RBD) with a loop region known as the receptor binding motif (RBM). The S2 domain consists of a fusion peptide (FP), heptad repeat (HR) 1 and 2, and a transmembrane (TM) and cytoplasmic (CP) domain (Figure 3). The S1 domain is responsible for the recognition of ACE2 and S2 mediates membrane fusion into the host cell. The sequence motif “KRSFIEDLLFNKV” is responsible for the initial binding of SARS-CoV to lung cells and activates the S protein by proteolytic cleavage (Robson, 2020).

The surface subunit S1 comprises 687 amino acids organized into SP, NTD, and RBD. The S1 subunit initiates the process of viral entry via attaching with the cell receptor. At the top of each S1 monomer, one RBD is present for interaction with ACE2. In the specified domain, RBDs undergo hinge-like conformational movement that transiently exposes an open state or a closed state (McKee et al., 2020). In particular, the extended loop region, RBM of the RBD contains the amino acid residues that bind to ACE2 (Lan et al., 2020). Comparing RBM of SARS-CoV-1 and SARS-CoV-2, the latter forms a larger binding interface and makes a large number of contacts with ACE2 with a higher binding affinity (K_d_ 31 and 4.7 nM, respectively) (Zhai et al., 2020).

Among the two domains S1 and S2, the SARS-CoV-2 S2 sequence shows 90% similarity with SARS-CoV-1. This suggests that the S2 domain is prone to less mutation and hence targeting the S2 domain might be useful in the preventive stage of viral infection. The shorter FP consists of 18 amino acids that play an important role in the fusion process and is responsible for the binding affinity toward the host cell. The HR1 and HR2 consist of a peptide sequence motif “HPPHCPC” representing hydrophobic (H), polar (P), and charged (C) residues. This sequence of the peptide region adopts an α-helix with a hydrophobic interface to drive the membrane fusion. Among the different variants of CoV, HR is highly conserved, and in particular, HR2 is 100% identical in comparison to the other regions, HR1 (88%), TM (93%), and CP (97%) in the S2 domain (Table 2). The TM is long enough in length to anchor the S protein in the membrane, has three conserved and distinctive domains namely N-terminal tryptophan-rich and hydrophobic central region ends with a cysteine-rich C-terminal domain. In the final section of the S2 domain, the CP tail has a high amount of S-acylated cysteine residues.

**TABLE 2:** Percent similarity of the various domains of the S protein for SARS-CoV-2 in comparison with SARS-CoV-1.

Domain	SARS CoV-1	Percent similarity (%)
S1	Overall	64
	NTD	51
	RBD	74
	RBM	50
S2	Overall	90
	FP	93
	HR1	88
	HR2	100
	TM	93
	CP	97

The SARS-CoV-2 S cleaves into S1 and S2 domains (Figure 3C). The S1 domain comprises of SP located at the N-terminus, RBD, and RBM. The S2 subunit has residues with FP, HR1, HR2, TM domain, and cytoplasm domain. The SARS-CoV-2 has 18 newly added amino acids when compared with SARS-CoV. The RBD in SARS-CoV has fewer amino acid residues when compared with SARS-CoV-2, which could be the reason for increased binding affinity toward the cellular receptor. Furthermore, there is no significant difference in amino acid residue between SARS and SARS-CoV-2 in the RBM region. The FP for both SARS and SARS-CoV-2 possess 18 conserved residues (Krishnamoorthy et al., 2020, Bahrami and Ferns, 2020).

## Binding Mechanism of the S Protein

**FIGURE 4:**
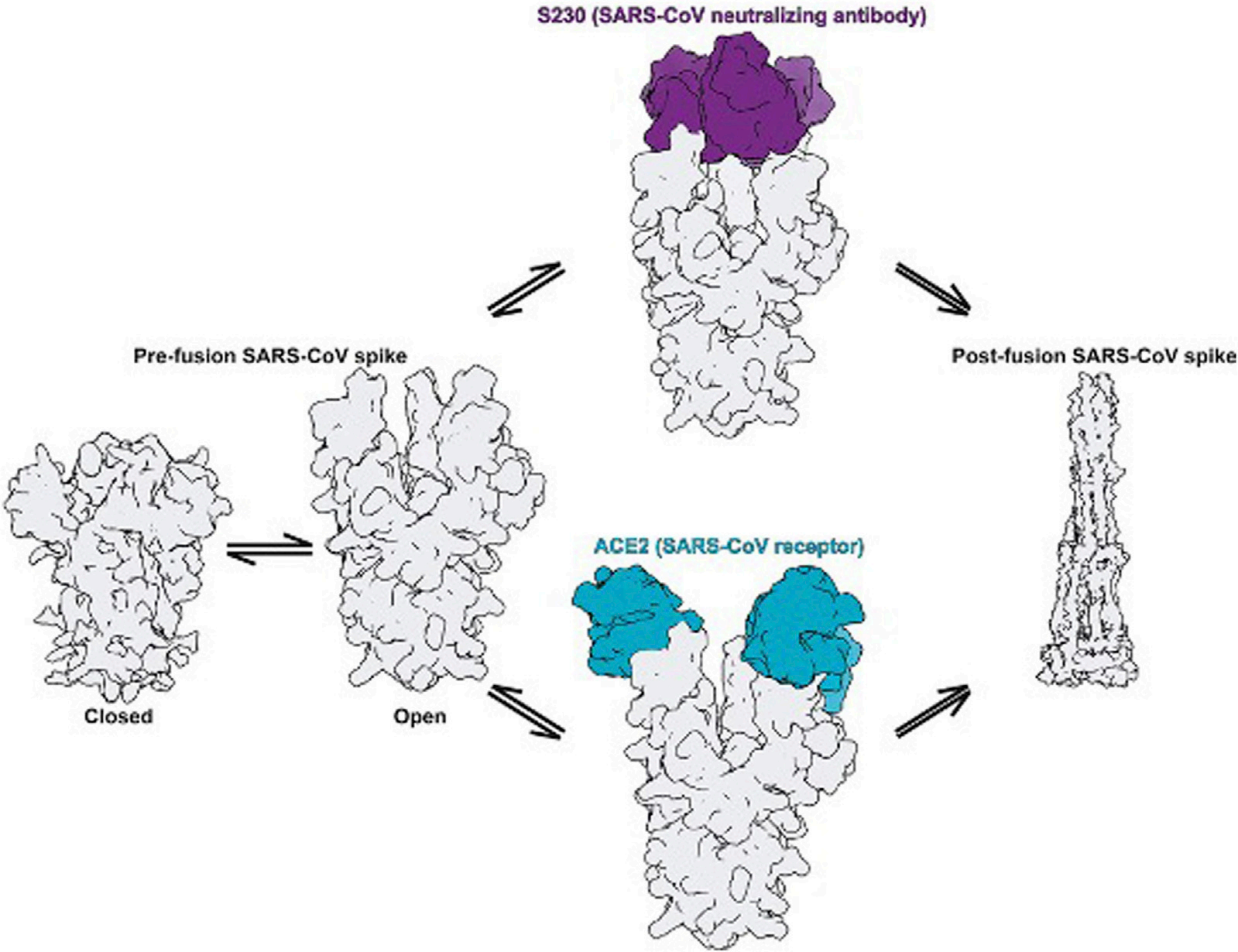
Fusion states in the S protein elucidate the mechanism of activation [(Walls et al., 2019) Copyright ©2020 Elsevier Inc., based on the reuse provisions of Elsevier’s COVID-19 Resource Center].

The entry of coronavirus through the S protein is a combined process involving receptor-binding and proteolytic processing to promote virus penetration into the host cell (Walls et al., 2020). In the pre-fusion conformation state, the S protein exists in the non-covalently bound state. This state reveals that the binding within the S protein is less stable and can open upon interaction with receptors as depicted in Figure 4. The RBD in S1 extends a loop to bind with the host peptidase domain (PD) of ACE2 through RBM. Once bound, the S2 domain undergoes structural rearrangement to activate the S protein for membrane fusion. This conformational change in the S2 domain causes the fusogenic potential to penetrate into the host cell. The heptad-repeat regions HR1 and HR2, gather into a six-helix bundle (HB) and bring the FP and cellular membrane in a hairpin conformation (Alnefaie and Albogami, 2020). The affinity between HR1 and HR2 against each other stabilizes this required conformation and confirms the fusion of the virus into the cellular membrane (Guo et al., 2020). The FP along with heptad regions HR1 and HR2 in the S2 domain assist viral fusion into the host cell (Liu et al., 2004; Xia et al., 2020). The fusion between the S2 domain and ACE2 receptor allows the spike to transform from the pre-fusion to the post-fusion conformation. The crystal structure of SARS-CoV-2’s RBD in complex with ACE2 showed that the RBD connects with the proteolytic domain (PD) of ACE2. At the N terminus, Q498, T500, and N501 of the RBD interacts via H-bond with Y41, Q42, K353, and R357 from ACE2. The RBD contacts via Y453, the ACE2 PD at the residue H34. In the C terminal region, van der Waals interactions are formed between Q474 of RBD and Q24 of ACE2, F486 of RBD, and M82 of ACE2.

The amino acid Q498 recognizes ACE2 and is responsible for infecting host cells; N501 helps in the transmission from human to human. L455 helps in viral binding to the ACE2 receptor. F486 supports binding and enhances viral infection. S494 provides positive support for enhancing the binding of the virus to ACE2. Upon viral infection, the post-fusion state begins with the activation of protease enzymes such as furin and TMPRSS-2 (Hoffmann et al., 2020b). The S protein of SARS-CoV-2 differs from SARS-CoV in its furin recognition site “RRAR” and is absent in other types of coronaviruses, making it a unique cleavage site (Coutard et al., 2020). This site can be an important target for inhibitors (Seidah and Prat, 2012). The S trimer is extensively decorated with N-linked glycans that are important for proper folding and for modulating accessibility to host proteases and neutralizing antibodies.

The advancement in crystallographic techniques provides a greater understanding of the structural biology of proteins. With the recent revolutionary advancement of cryo-EM, the number of protein structures obtained is growing at a very high rate and in particular protein structures are being found that are difficult to crystallize. This is visible from the number of reported structures (129 structures) for SARS-CoV-2. Supplementary Table S1 shows the summary and list of available crystal structures of the S protein along with the complex. The high resolution (1.5 Å) structures using X-ray crystallography are available for MERS-CoV (PDB ID: 5 × 4R), SARS-CoV-1 (PDB ID: 1ZVA), and SARS-CoV-2 (PDB ID: 6M1V). In the current pandemic situation, the application of these techniques provides a structural understanding of CoVs which is greatly important (163 structures are reported in 2020) for drug discovery and development. The structures of the spike protein from MERS, SARS-CoV-1, and -2 were determined and in particular, the receptor binding domain (RBD), N-terminal domain (NTD), and C-terminal domain (CTD) were predominately reported due to their importance in binding with the host cell receptors. The reported crystal structures with different antibodies help to form structures and understand the binding mechanism of the S protein, the functional movements of the domains, and their involvement in binding with the host cell receptor. Specifically, detailed information was obtained for SARS-CoV-2 with the ACE2 receptor.

The open reading frames (ORF) ORF1a and ORF1b are translated into polyproteins pp1a (4382 amino acids) and pp1ab (7073 amino acids). These polyproteins are processed by 3-C-like protease (3CL^Pro^) and papain-like protease (PL^Pro^) to generate a variety of non-structural proteins (NSPs), including RNA-dependent RNA polymerase (RdRp) and helicase, for catalyzing viral genome replication and protein synthesis.

## Targeting the S Protein

### Vaccines

Since the first outbreak of SARS-CoV-1 in 2002, there has been active involvement in the development of vaccines against coronaviruses (Jiang et al., 2012; Modjarrad et al., 2016; Zhou et al., 2019; Dai et al., 2020; Wang C. et al., 2020). The recent outbreak of SARS-CoV-2 affected a large number of people and streamed larger efforts in the development of vaccines. As of November 2020, WHO has not recommended any vaccines for SARS-CoV-2, but a few of the vaccines are closer to approval in selected countries. The vaccine development targeting the S protein can be grouped as full-length S protein, RBD, and RNA.

#### Full-Length S Protein

A great deal of interest and focus on developing vaccines has targeted the full-length S protein of SARS-CoV. The vaccine for the full-length S protein showed required immunity against SARS-CoV-1 suppressing viral proliferation but resulted in a harmful immune response (Jiang et al., 2005). The vaccine-induced antibodies against SARS-CoV-2 bind with the virus. In this, neutralizing antibodies provides efficient blockade for viral infection and non-neutralizing antibodies generate an antibody-dependent enhancement effect that can aggravate the infection (Garber, 2020; Iwasaki and Yang et al., 2020; Tetro, 2020; Ulrich et al., 2020). The studies on MERS-CoV neutralizing single-domain antibodies (sdAbs) from immunized dromedary camels and llamas showed EC_50_ values between 0.001–0.003 μg/ml and low K_d_ values in the range of 0.1–1 nM (Seidah and Prat, 2012). Furthermore, the sdAbs showed an EC_50_ of 0.0009–0.07 μg/ml and 0.13–0.51 μg/ml against SARS-CoV-2 pseudotypes, and authentic SARS-CoV-2, respectively (Chi et al., 2020). Liu L et al. identified that anti S protein immunoglobulin (IgG) on administration in healthy macaque with SARS-CoV infection, resulted in severe acute lung injury due to antibody-dependent enhancement (ADE) induced by peptides 597–603 of the S protein (Wang et al., 2016; Liu et al., 2019). Further identifying the antibodies that cause infections and avoiding ADE has to be considered in the vaccine development targeting the full-length S protein.

#### RBD-sc DIMERS in Vaccine Development

Due to the drawbacks of the full-length S protein vaccine, the focus shifted to the RBD region as the vaccine candidate. The antigenic epitopes from the RBD of SARS-CoV neutralize the antibodies as well as the CD8^+^ T cell responses. The RBD-dimer vaccine significantly increased neutralizing antibodies since it exposed dual receptor-binding motifs and protected mice against MERS-CoV infection better than RBD-monomer (Dai et al., 2020). This strategy has led to the design of a vaccine for SARS-CoVs with 10–100 fold enhancement of neutralizing antibodies. A recent study on a vaccine based on RBD against SARS and MERS found good efficacy (Jiang et al., 2012; Modjarrad et al., 2016; Dai et al., 2020; Wang and Tu, 2020) but also found a few limitations including low immunogenicity, protein sequences, and fragment lengths. The RBD vaccine generates potent antibodies and provides sustained protection when compared with the full-length S protein vaccine (Yang et al., 2020). A recombinant RBD protein-based vaccine is also equally effective but requires repeat doses.

The epitope can be used to develop vaccine as it can stimulate immune responses using isolated B cell or T cells, and the use of multiple epitopes can further improve vaccine efficacy. Recently five epitopes were identified through literature mining located in the fully exposed RBD hotspot regions of the S protein possessing antigenicity including three B cell epitopes (“RQIAPGQTGKIADYNYKLPD,” “SYGFQPTNGVGYQ,” and “YAWNRKRISNCVA”) and two T cell epitopes (“KPFERDISTEIYQ” and “NYNYLYRLFR”) (Li et al., 2020). All five epitopes were found to be non-toxic and have the potential to be developed as a vaccine candidate.

#### mRNA Vaccine

In the epidemic of SARS-CoV-2, the development of mRNA vaccines has gained huge interest. The flexibility in the design of the RNA vaccine has made it more advantageous during the pandemic. The RNA vaccine is well tolerated by the human body and is considered to be safe. In an RNA vaccine, the genetic information for the antigen is delivered generally through a lipid nanoparticle. Currently, many mRNA vaccines are under development for viruses other than SARS-CoVs like Zika and cytomegalovirus. Among the 51 vaccines in clinical trials for SARS-CoVs, six of the vaccines are based on RNA (https://www.who.int).

The vaccine mRNA-1273 developed by Moderna Therapeutics in collaboration with the National Institute of Allergy and Infectious Disease Vaccine Research Center (NIAID VRC) is based on mRNA that encodes for a full-length, prefusion stabilized S protein of SARS-CoV-2 encapsulated by a novel lipid nanoparticle. The mRNA-1273 vaccine is currently in Phase III and is in the process of approval in selected countries.

Another mRNA-based vaccine candidate is BNT162b2 which encodes a full-length S protein with two stabilizing proline residues developed by BioNTechin in collaboration with Fosun Pharma and Pfizer. This vaccine is in Phase III clinical trials. BNT162b2 was found to be 95% effective against SARS-CoV-2 after 28 days of the first dose and showed a good safety profile (Mulligan et al., 2020). The European Medical Agency has received the application for conditional marketing authorization for BNT162b2.

Furthermore, the CVnCoV vaccine developed by CureVac is under Phase II, the Lunar-COV19 vaccine by Arcturus/Duke-NUS is in Phase I/II, and two vaccines from Imperial College London and People’s Liberation Army (PLA) Academy of Military Sciences/Walvax Biotech are in Phase I clinical trials (c.f. Table 3). Still the current vaccines are in the developmental phase and the process of approval is unclear about issues including bulk production, stability, storage, and mucosal immunity upon injection (Krammer, 2020).

**TABLE 3:** List of mRNA vaccines in various stages of clinical trials.

Vaccine name	Company name	Clinical status
mRNA-1273	Moderna/NIAID	Phase III
BNT162b2	BionTech/Fosun pharma/Pfizer	Phase III
CVnCoV	CureVac	Phase II
LUNAR-COV19	Arcturus/Duke-NUS	Phase I/II
LNP-nCoVsaRNA	Imperial college London	Phase I
ARCoV	People’s liberation army academy of military sciences/Walvax biotech	Phase I

### Human Monoclonal Antibody Targeting RBD in Vaccine Development

Targeting only the RBD reduces the levels of antibody titer thereby making it a safe and efficacious target. Tian et al. revealed that the most potent SARS-CoV-specific neutralizing antibodies (e.g., m396, CR3014, CR30222) targeting the ACE2 binding site of SARS-CoV failed to bind the SARS-CoV-2 S protein (Tian et al., 2020). This indicates that changes in the amino acid could have caused the exacerbation of antibodies. The effect on cross-neutralizing antibodies has to be further studied for targeting RBD in the development of vaccines.

### Inhibitors

#### Small Molecule Inhibitors

SARS-CoV-2 transfers into the human cell by first binding the spike of the S protein with the host cell receptors. The S protein of SARS-CoV-2 shows an 80% similarity with SARS-CoV-1 and a 96% similarity with bat-CoV RaTG13 (Zhou et al., 2020). Zhang et al. also revealed that the genome sequence of SARS-CoV-2 has 89.1% similarity toward SARS-like coronaviruses (Wu et al., 2020). SARS-CoV-2 uses the ACE2 receptor for entry into the host cell similar to SARS-CoV-1 (Zhou et al., 2020). The RBD from SARS-CoV-2 and SARS-CoV-1 that interact with ACE2 are found to have 74% similarity (Yan et al., 2020). Plasmin resonance spectrometry uncovered that the RBD of the spike of SARS-CoV-2 has a high affinity (K_d_ = 14.7 nM) for the ACE2 receptor of the host cell (Wrapp et al., 2020).

ACE is a central component of the renin-angiotensin system and controls blood pressure. It is a highly glycosylated type I integral membrane protein and converts angiotensin I to angiotensin II. Though ACE1 and ACE2 both cleave the peptide, there is a significant difference in their mechanism of function. Angiotensin (Ang) I (a decapeptide) is converted into Ang II (an octapeptide) by ACE1. This involves dipeptide His-Leu from Ang I to form Ang II. This process is responsible for vaso- and broncho-constriction, increased vascular permeability, inflammation, fibrosis, and thereby causing acute respiratory distress syndrome (ARDS) and lung failure (Yang et al., 2014).

CoVs use two receptor binding pathways, viz., clathrin (endosomal) and non-clathrin pathways (non-endosomal) (Inoue et al., 2007; Wang et al., 2008). In the clathrin pathway, the S protein of the CoV binds to the host receptor and embodies vesicles that mature to late endosomes. These endosomes get acidified and stimulate the H^+^-dependent activation of cellular cathepsin L proteinase in late endosomes and lysosomes, cleaving and activating the S protein which initiates viral fusion. SARS-CoV-2 also uses host cell receptor CD147 along with ACE2 for entry into the host cell (Wang K. et al., 2020). In the non-clathrin pathway, membrane fusion is the critical stage in the CoV life cycle. Membrane fusion is activated by cleavage of the host proteases include cathepsin L, TMPRSS2, and TMPRSS1 1D (airway trypsin-like protease) at the S1/S2 cleavage site (Shirato et al., 2013). These proteases are also an attractive target for SARS-CoV-2 (Zhou et al., 2015). Recently, the significance of TMPRSS2 in the life cycle of SARS-CoV-2-infected Vero E6 cells was confirmed (Geller et al., 2012). Table 4 illustrates the cellular receptors in the coronaviruses.

**TABLE 4:** Classification and cellular receptor of the coronaviruses.

HCoV genera	Coronaviruses	Cellular receptor
α-coronaviruses	HCoV-229E	Human aminopeptidase N (CD13)
	HCoV-NL63	ACE2
β-coronaviruses	HCoV-OC43	9-*O*-acetylated sialic acid
	HCoV-HKU1	9-*O*-acetylated sialic acid
	SARS-CoV-1	ACE2
	MERS-CoV	DPP4
	SARS-CoV-2	ACE2

#### S Domain Inhibitors

**FIGURE 5:**
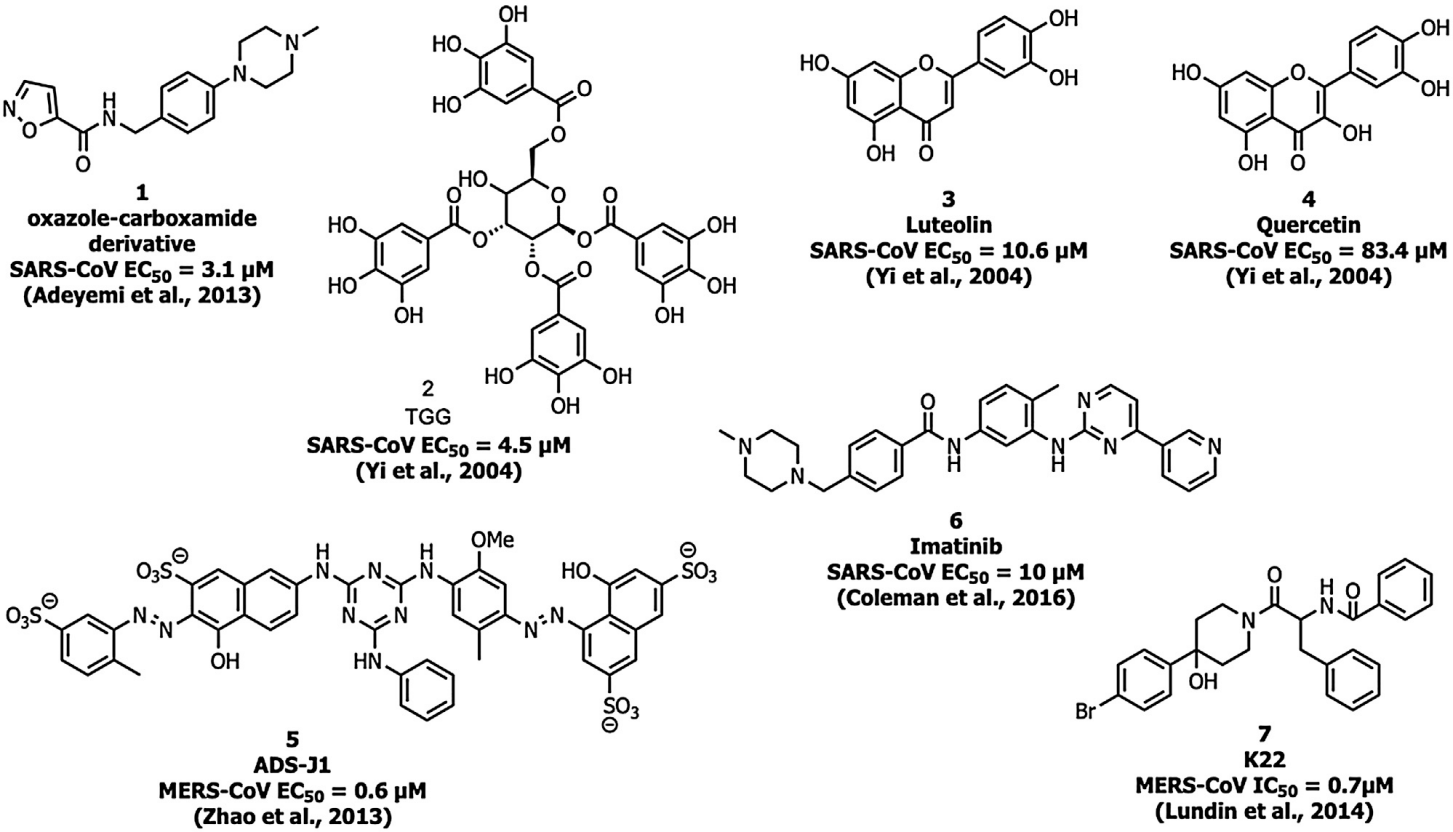
Inhibitors targeting the spike protein S domains S1 and S2.

Adedeji et al. screened a chemical library of 3000 molecules for the SARS-CoV-1 entry inhibitor and identified an oxazole-carboxamide derivative (Figure 5) (1) as a lead molecule that interferes with the RBD blocking ACE2 recognition. Compound 1 showed inhibition with an EC_50_ value of 3.1 µM and a 50% cytotoxic concentration (CC_50_) value of >100 μM, but it did not affect the expression levels of ACE2 (Adedeji et al., 2013).

Yi and his coworkers identified two molecules, TGG (2) and luteolin (3) that inhibited the viral entry into Vero E6 cells by binding with the S2 protein of SARS-CoV-1 (Yi et al., 2004). Compounds 2 and 3 exhibited an EC_50_ of 4.5 and 10.6 µM, respectively, and CC_50_ of 1.08 and 0.155 mM with a selectivity index (SI) of 240.0 and 14.62, respectively. The compounds 2 and 3 were safe up to 232.2 and 456 mg/kg, respectively in the LD_50_ acute toxicity study. An analog of compound 3, quercetin (4) showed inhibitory activity at EC_50_ = 83.4 µM and CC_50_ = 3.32 mM (Yi et al., 2004).

Small-molecule HIV entry inhibitor, ADS-J1 (5) inhibits >90% of MERS-CoV pseudovirus infection in NBL-7 and Huh-7 cells at a concentration of 20 µM (Zhao et al., 2013). ADS-J1 inhibits the entry of pseudotyped MERS-CoV (EC_50_ = 0.6 µM) in the DPP4-expressing cell line and CC_50_ with 26.9 µM in NBL-7 and Huh-7 cells by MTT assay by forming a six-helix bundle and interrupting the interactions between HR1 and HR2 of MERS-CoV. Chu et al. identified that ADS-J1 (5) also possesses potential inhibitory activity against SARS-CoV-1 viral entry (EC_50_ = 3.89 µM) (Chu et al., 2008).

An Abelson kinase inhibitor, imatinib (6) inhibits S protein-induced fusion of coronaviruses including SARS-CoV, MERS-CoV, and infectious bronchitis virus (IBV) at 10 µM and without cytotoxic effects in Vero cells up to concentrations of 100 µM (Coleman et al., 2016; Sisk et al., 2018).

Lundin et al. screened a library of 16,671 diverse set of molecules and identified a small molecule inhibitor, K22 (7), which inhibited HCoV-229E with an IC_50_ value of 0.7 μM K22 targets the initial stage in the life cycle of HCoV-229E and possibly interacts with viral particles and results in the inactivation state of the virus (Lundin et al., 2014).

#### ACE2 Inhibitors

**FIGURE 6:**
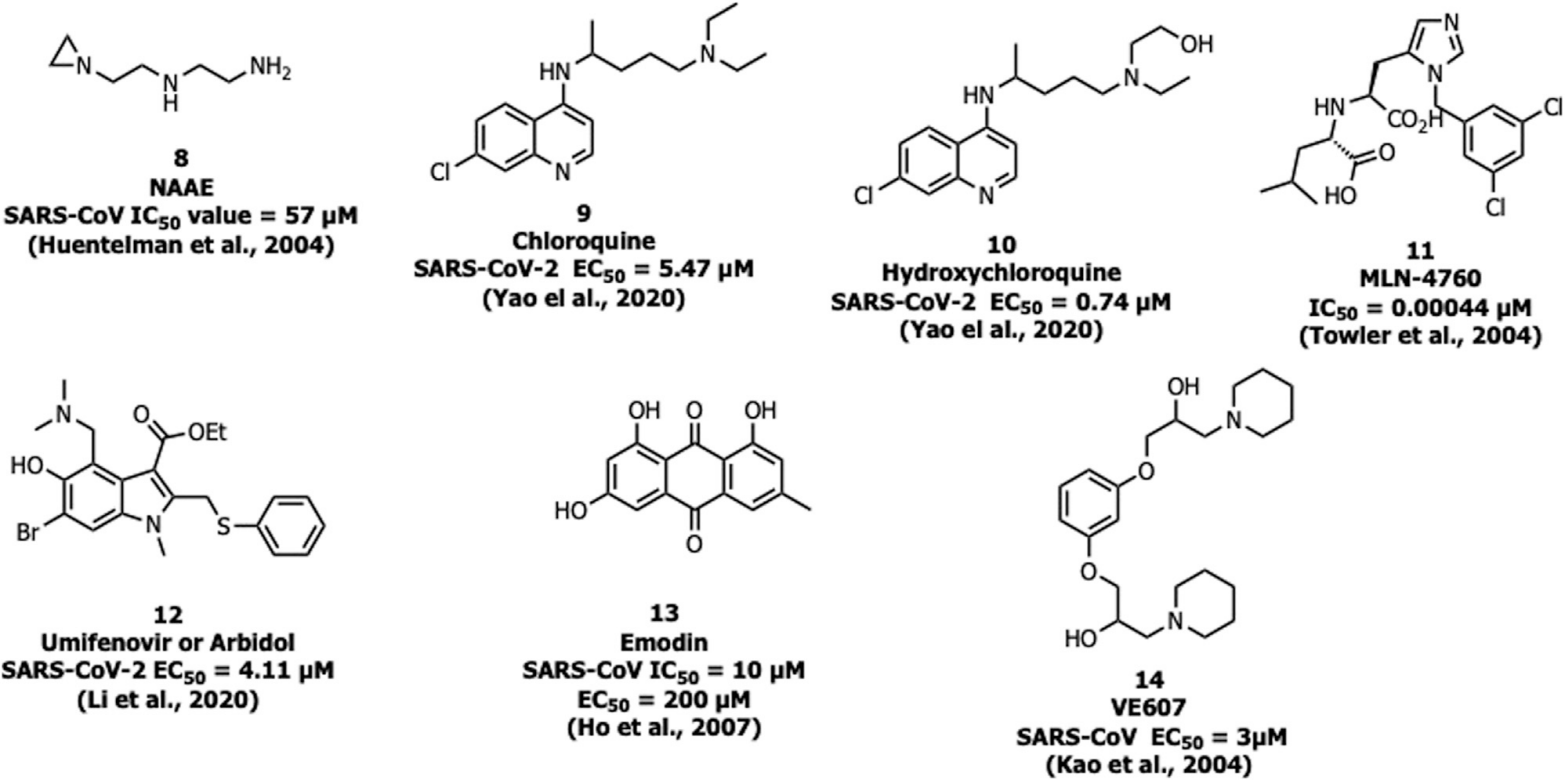
Inhibitors for SARS-CoV-1 and -2 targeting ACE2.

*N*-(2-aminoethyl)-1-aziridine-ethanamine (NAAE, 8) was identified as a potent ACE2 inhibitor with an IC_50_ value of 57 µM and *K*_i_ value of 459 µM (Figure 6) from a virtual screening of 140,000 compounds which inhibited SARS-CoV-1 by modulating S-glycoprotein-mediated membrane fusion (Huentelman et al., 2004). Savarino et al. reported the antiviral property of chloroquine (9), one of the safe and cost-effective drugs for the management of malaria and amebiasis (Savarino et al., 2003). Chloroquine showed good *in vitro* activity against almost all lethal forms of coronavirus, SARS-CoV-1, MERS-CoV, and SARS-CoV-2. Against SARS-CoV-2, chloroquine showed an EC_50_ value of 5.47 µM (Keyaerts et al., 2004; Devaux et al., 2020; Yao et al., 2020). It is assumed that chloroquine inhibits the production of proinflammatory cytokines (such as interleukin-6) by reducing acute respiratory distress syndrome (ARDS) (Savarino et al., 2003). The mechanistic study showed that chloroquine interferes with the terminal glycosylation of ACE2 and affects the interaction between the RBD of SARS-CoV-1 and ACE2 (Vincent et al., 2005).

A derivative of chloroquine, hydroxychloroquine (10) is another antimalarial drug experimented with against SARS-CoV-2, but still, the benefits are unclear (Mahase, 2020). It inhibits SARS-CoV-2 *in vitro* with an EC_50_ value of 0.74 µM (Yao et al., 2020). In March 2020, WHO announced that chloroquine and hydroxychloroquine were involved in the clinical trials for the treatment against SARS-CoV-2 (https://www.who.int). The trials were initiated by the US National Institutes of Health (NIH) in April 2020 and the study involved 96,032 subjects affected by SARS CoV-2, however, it is not clear regarding the effective benefits of hydroxychloroquine or chloroquine alone or in combination with macrolides against SARS-CoV-2 (like azithromycin or clarithromycin) (Mehra et al., 2020). Due to safety precautions, in May 2020, WHO announced that the clinical trials were stopped on using hydroxychloroquine as a drug for the treatment against SARS-CoV-2 (https://www.who.int).

One of the most potent and selective small-molecule inhibitors so far against ACE2 is MLN-4760 (11) with an IC_50_ of around 440 pM. It interacts with the zinc active site and imitates the transition state peptide. Hence MLN-4760 can be a useful inhibitor in the prevention of viral binding to ACE2 and results in the blockage of infection (Towler et al., 2004).

Umifenovir or arbidol (12) is a broad-spectrum inhibitor used as an antiviral drug against influenza. Arbidol inhibits the virus-host cell fusion and prevents the entry of virus which is also applicable for coronavirus (Kadam and Wilson, 2017), and currently the drug is under clinical trials for the treatment of SARS-CoV-2 (Li and De Clercq, 2020). In another study, arbidol (12) was found to decrease the viral load and act by binding with the S protein, and was involved in trimerization that inhibits the host cell and membrane fusion (IC_50_ = 4.11 μM) (Wang et al., 2020).

Ho et al. reported that the active component from *Polygonum multiflorum* and *Rheum officinale*, emodin (13), blocks S protein interaction with ACE2 with an IC_50_ value of 10 µM and an EC_50_ value of 200 µM (Ho et al., 2007).

Recently, three selected compounds among 50,240 structurally diverse molecules, MP576, HE602, and VE607 (14) were evaluated against SARS-CoV M^pro^, helicase, and viral entry, respectively using a phenotype-based screening. Among them, VE607 (14) ((1-[3-(2-Hydroxyl-3-piperidin-1-yl-propoxy)-phenoxy]-3-piperidin-1-ylpropan-2-ol)), blocked the SARS-CoV S protein pseudotype virus infection of 293T cells expressing ACE2 with an EC_50_ of 3 μM and inhibited SARS-CoV plaque formation with an EC_50_ of 1.6 μM (Kao et al., 2004).

Hanson et al., performed drug repurposing of 3384 small molecule drugs with 25 hits using a proximity-based assay that measures the binding of SARS-CoV-2 RBD and ACE2 (Hanson et al., 2020). Even though the unbound states of the S protein and ACE2 lacks druggable pockets, there are well-defined pockets in the bound states for drug development. By application of computational approaches, Patil et al. showed that several antiviral drugs used against HCV and HIV viruses, e.g., atazanavir, grazoprevir, saquinavir, simeprevir, telaprevir, and tipranavir, could serve as immediate investigational molecules and possibly as a potential candidate inhibitor (Patil et al., 2020).

#### Proteolytic Inhibitors

**FIGURE 7:**
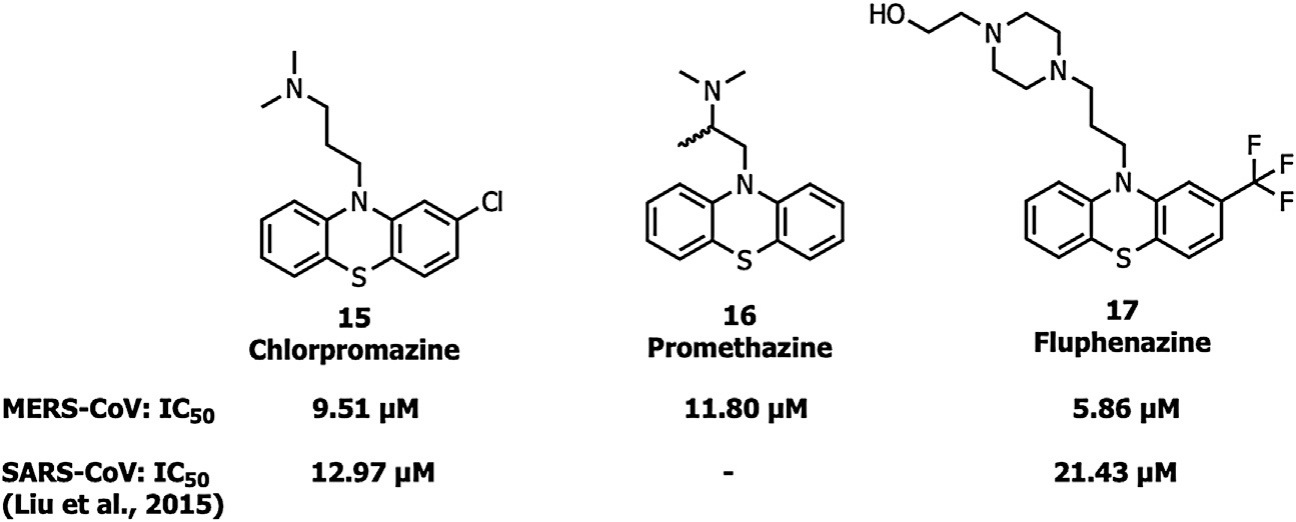
Neurotransmitter inhibitors targeting clathrin/non-clathrin pathways.

Chlorpromazine (15), promethazine (16), and fluphenazine (17) neurotransmitter blockers (Figure 7) inhibit S protein-induced fusion of MERS-CoV and SARS-CoV-1 (Liu et al., 2015). Chlorpromazine, an inhibitor of clathrin-mediated endocytosis, was already reported to inhibit human CoV-229E, hepatitis C virus, infectious bronchitis virus, as well as mouse hepatitis virus-2 (MHV2) (Krizanová et al., 1982; Joki-Korpela et al., 2001; Nawa et al., 2003; Chu and Ng, 2004).

**FIGURE 8:**
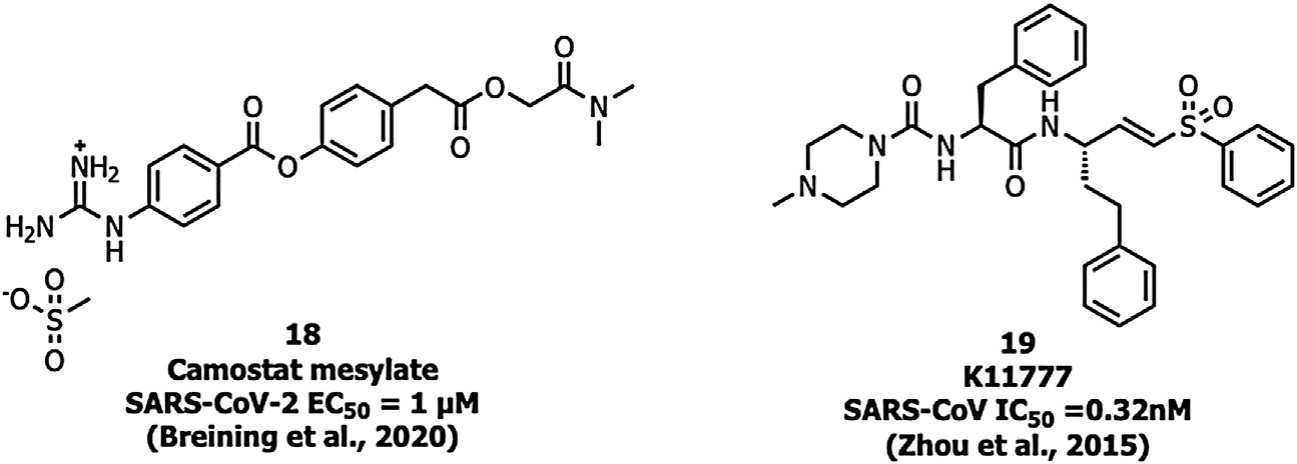
Inhibitors targeting TMPRSS2.

Ouabain and bufalin inhibitors block clathrin-mediated endocytosis and prevent MERS-CoV entry. Ouabain (50 nM) and bufalin (10–15 nM) inhibited infections by MERS-CoV and VSV (vesicular stomatitis virus). Breining et al. identified camostat (18), a protease inhibitor, as a TMPRSS2 blocker at 10 µM in SARS-CoV-1. However, at a higher concentration (100 µM) the inhibition efficiency was only up to 65% which shows that 35% of entry happens via the endosomal cathepsin pathway. The study also showed >95% blockade of viral entry with a combination treatment of EST (a cathepsin inhibitor) and 18 (Breining et al., 2000). Complete inhibition of viral entry was also observed with a combination of both 18 and E-64d (a cathepsin inhibitor) (Hoffmann et al., 2020a). Tissue cultures of another cysteine protease inhibitor, K11777 (19), (Figure 8) showed inhibition in the sub-nanomolar range against the replication of SARS-CoV-1 and MERS-CoV (Zhou et al., 2016). However further studies using tissue culture and animal models need to be carried out to confirm TMPRSS2 inhibition.

Teicoplanin blocks the entry of SARS-CoV-2 pseudoviruses (IC_50_ = 1.66 µM). Teicoplanin is a glycopeptide antibiotic used in the prophylactic treatment of Gram-positive bacterial infections including methicillin-resistant *Staphylococcus aureus* and *Enterococcus faecalis*. It is also an inhibitor of cathepsin L of SARS-CoV-1, MERS-CoV, and Ebola virus, and prevents viral entry (Zhang et al., 2020).

**FIGURE 9:**
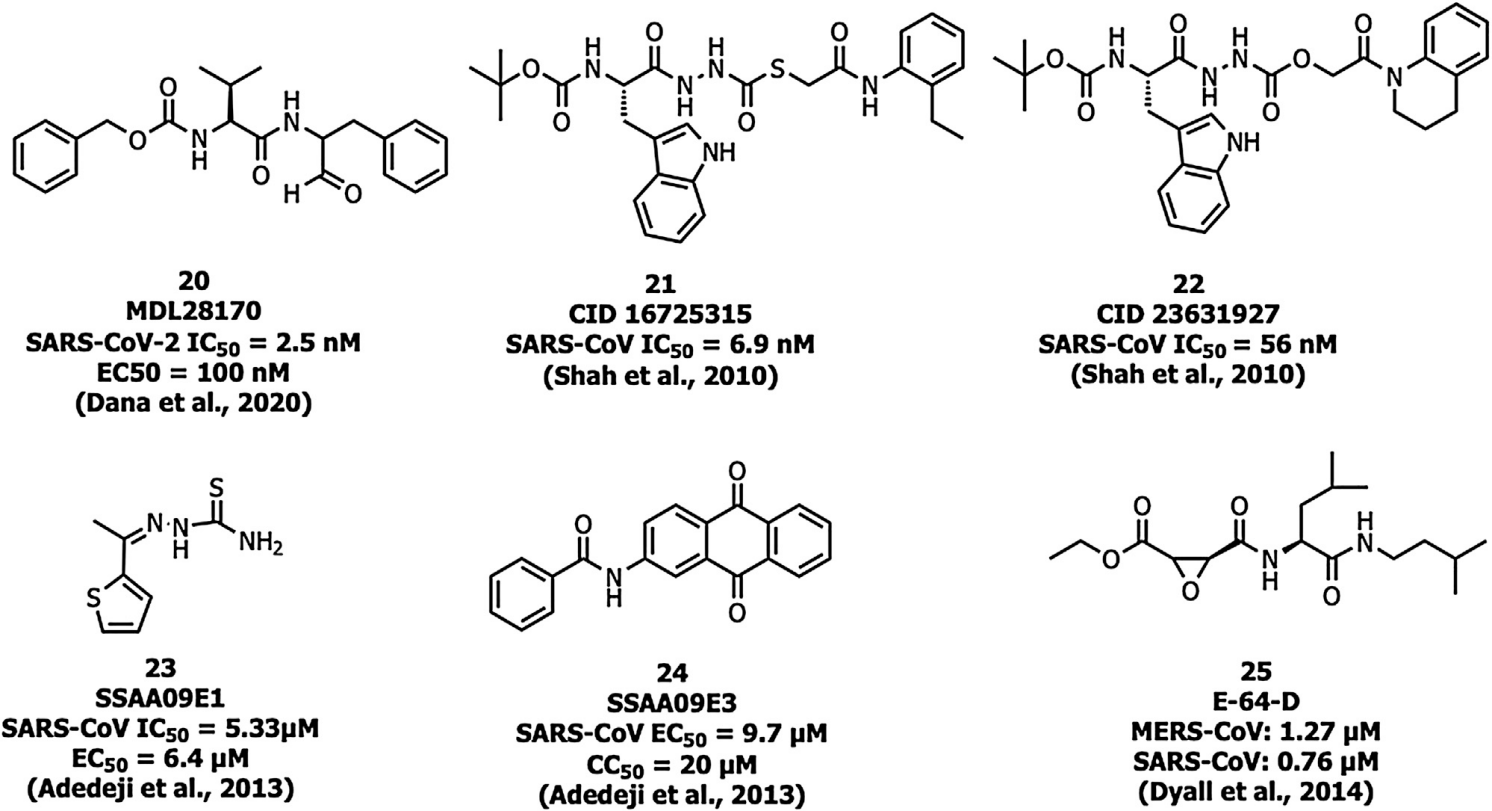
Cathepsin L inhibitors with antiviral activity.

Human cathepsin L, a cysteine endopeptidase, activates the S protein into a fusogenic state to escape the late endosomes, and thereby interferes with viral entry (Dana and Pathak, 2020). MDL28170 (20) (Figure 9) inhibits cathepsin-L-mediated substrate cleavage with IC_50_ and EC_50_ values of 2.5 nM and 100 nM, respectively (Simmons et al., 2005). CID 16725315 (21) and CID 23631927 (22) are SARS-CoV cathepsin L inhibitors reported with an IC_50_ value of 6.9 nM and 56 nM, respectively (Shah et al., 2010). SSAA09E1 (23) was identified as an inhibitor of cathepsin L proteinase among ∼14,000 compounds with an IC_50_ value of 5.33 µM. The compound 23 showed an EC_50_ value of around 6.4 µM in a pseudotype-based assay in 293T cells and was non-cytotoxic below 100 µM (Adedeji et al., 2013).

Adedeji et al. reported SSAA09E3 (24) as an inhibitor of virus-cell membrane fusion in pseudotype-based and antiviral-based assays. The viral entry inhibitor compound 24 showed an EC_50_ value of 9.7 µM, and a CC_50_ value of 20 µM against a pseudotype-based assay in 293T cells (Adedeji et al., 2013). E-64-D (25) blocked cathepsin of both MERS-CoV and SARS-CoV-1 infections (Dyall et al., 2014; Lu et al., 2014).

#### Glucose Regulation Protein 78 Inhibitors

Understanding the M, E, and NSP6 proteins suggest that the SARS-CoV S protein activates several unfolded protein response (UPR) effectors such as glucose-regulated protein (GRP) 78, GRP94, and CCAAT/enhancer-binding protein (C/EBP) homologous protein during the transcription process. Endoplasmic reticulum (ER) stress and UPR are induced in infected cells during CoV infection. GRP94 and GRP78 or binding immunoglobulin protein (BiP) are molecular chaperones and sensitive markers of ER stress (Chan et al., 2006).

The main UPR responsible for the viral entry including human and bat coronaviruses is GRP78. GRP78 is a luminal protein abundantly present in the ER and translocates to the cell surface during ER stress or coronavirus infection. After translocation to the cell surface membrane, GRP78 recognizes the virus by the substrate-binding domain (SBD) and mediates the entry of the virus into the cell. Further, it also plays a major role in the synthesis of viral protein, maturation, and inactivates three enzymes responsible for cell death or differentiation, *viz*., activating transcription factor (ATF) 6, protein kinase RNA-like endoplasmic reticulum kinase (PERK), and inositol-requiring enzyme (IRE) 1. Once the threshold of UPR accumulation is reached, these enzymes are released by the GRP78 and inhibit protein synthesis, and enhance refolding (Chang et al., 2006; Ha et al., 2020; Ibrahim et al., 2020).

GRP78 is a crucial element for a viral infection to new cells. Depletion of GRP78 leads to a decrease in protein synthesis or improper folding of viral proteins and results in impaired budding or immature virions with diminished infectivity. GRP78 maintains the ER homeostasis and thereby expedites viral component assembly by providing an ecosystem for growth. It is also captured into the viral particle and augments infection (Ha et al., 2020). It would be highly advantageous to inhibit the interaction between the S protein of SARS-CoV-2 and host cell receptor GRP78 to diminish the viral infection rate (Ibrahim et al., 2020).

Rayner et al. determined that AR12 (a derivative of celecoxib; 26) inhibits the production of the S protein of SARS-CoV-2 and thereby suppresses infectious virion generation. Compound 26 decreases ACE2 and GRP78 expression in the cell surface and total GRP78 levels. Compound 26 not only catalytically inhibits the GRP78 ATPase activity but also reduces the chaperone proteins, which are linked with low S protein and the production of infectious virions (Rayner et al., 2020).

**FIGURE 10:**
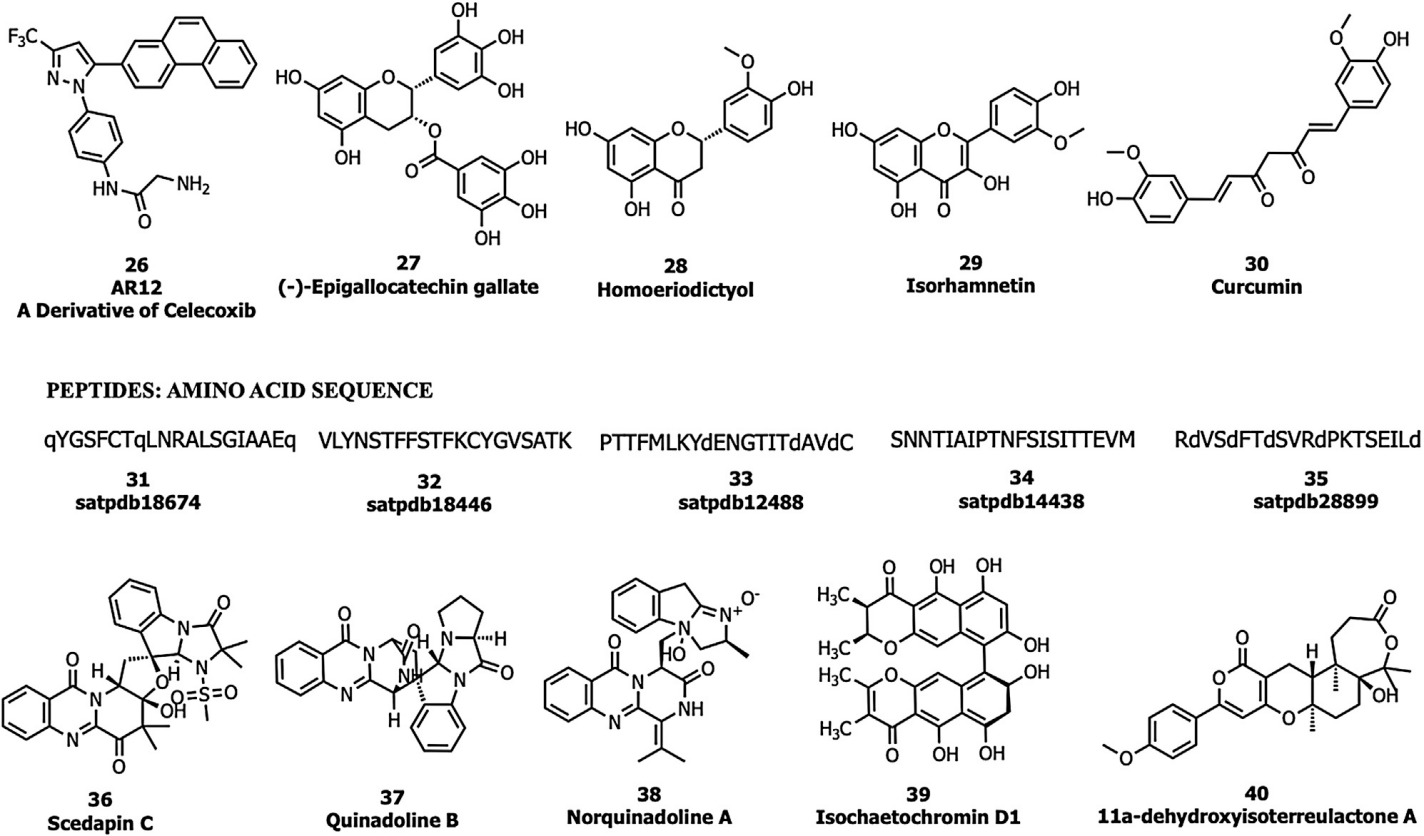
GRP78 inhibitors.

Allam et al., performed an *in silico* screening of a library of compounds and identified four potential phytochemicals (polyphenols, *viz*., epigallocatechin gallate (EGCG; 27), homoeriodictyol (28), isorhamnetin (29), and curcumin (30)) and five peptides (satpdb18674 (31), satpdb18446 (32), satpdb12488 (33), satpdb14438 (34), and satpdb28899 (35)) that inhibited the interaction of the SARS-CoV-2 S protein with GRP78 using molecular docking approaches (Allam et al., 2020), Quimque et al. docked 97 antiviral molecules from fungi secondary metabolites followed by molecular dynamics simulation and in silico ADMET prediction. Three fumiquinazoline alkaloids, scedapin C (36), quinadoline B (37), and nor-quinadoline A (38); the polyketide isochaetochromin D1 (39); and the terpenoid 11a-dehydroxyisoterreulactone A (40) exhibited strong in silico inhibition against GRP78 of SAR-CoV-2 (Quimque et al., 2020) (Figure 10).

#### Peptide-based Inhibitors

Peptide-based or peptidomimetic inhibitors are larger molecules consisting of amino acid linkages with molecular size ranging from 1137 to 1814 Da (Ou et al., 2020). Peptide-based inhibitors are hypothesized to prevent the entry of the virus into human cells by disrupting the interaction of RBD of SARS-CoV-2 and ACE2. In a molecular dynamics simulation study carried out by Zhang et al. (2020), the protein-protein interactions were analyzed between the SARS-COV-2 S protein and human ACE2. The S protein binding peptide 1 (SBP1; 41) was synthesized with a sequence of 23 amino acids derived from the ACE2 α1 helix and a dissociation constant K_d_ of 14.7 nM suggesting that SBP1 binds with the RBD of the S protein with low nanomolar affinity. The peptide inhibitor found to prevent entry of the virus into human cells (Coutard et al., 2020).

Ho et al. reported that peptide molecules significantly blocked the interaction of the S protein with ACE2 (IC_50_ = 1.88 nM) (Ho et al., 2006). Han et al. stated that charged residues located at positions 22 and 57 are critical for the entry of virus (Han et al., 2006). Based on this concept, various peptides were synthesized and two compounds were found, 42 (IC_50_ = 50 µM) and 43 (IC_50_ = 6.0 µM), with significant inhibitory activity against SARS-CoV-1. The introduction of a glycine binding linker in compound 42 with an ACE2-derived peptide (residues 351–357) further improvised the activity (IC_50_ = 100 nM) and reduced the cytotoxicity up to 200 µM (Han et al., 2006).

An *in silico* design of an antiviral (Seidah and Prat, 2012; Chan et al., 2020) HR2-derived peptide-like structure showed competitive inhibition of the binding of the HR2 domain to the HR1 domain (Bosch et al., 2004). It should also be noted here that the HR1-derived peptide failed to inhibit the viral infection due to the antiparallel binding of HR1 with three HR2 domains. This evidence suggests that targeting HR2 with HR2-derived peptides might prove a promising strategy in drug design against SARS-CoV-2.

Xia et al. reported that a potent fusion inhibitor, EK1C4 (44), lipopeptide-targeted the S-glycoprotein-mediated cell membrane fusion of SARS-CoV-2, pseudotyped SARS-CoV-2, and live SARS-CoV-2 infection with IC_50_ values of 1.3, 15.8, and 36.5 nM, respectively (Xia et al., 2020). IPB02 (45), another lipopeptide fusion inhibitor targeting the HR1 region was developed (Zhao et al., 2013), which restricted the cell fusion activity of SARS-CoV-2 S-glycoprotein (IC_50_ = 25 nM) and SARS-CoV-2 pseudovirus (IC_50_ = 80 nM) (Table 5).

**TABLE 5:** Peptide inhibitors targeting the S protein.

Peptide	Peptide sequence	Coronavirus	Activity
SBP1 (41)	IEEQAKTFLDKFNHEAEDLFYQS	SARS-CoV-1	K_d_ = 14.7 nM
42	EEQAKTFLDKFNHEAEDLFYQSS	SARS-CoV-1	IC_50_ = 50 µM
43	EEQAKTFLDKFNHEAEDLFYQSSLASWNYNTNITEE	SARS-CoV-1	IC_50_ = 6.0 µM
EK1C4 (44)	SLDQINVTFLDLEYEMKK.EEAIKKLEESYIDLKEL-GSGSG-PEG4-Chol	SARS-CoV-2	IC_50_ = 1.3 nM
IPB02 (45)	ISGINASVVNIQKEIDRLNEVAKNLNESLIDLQELK (Chol)	SARS-CoV-2	IC_50_ = 25 nM

#### Natural Product Inhibitors

Heparin, a natural anti-coagulant was explored as an antiviral agent for SARS-CoV, herpes, flavivirus, influenza, and HIV. A recent study has explored that SARS-CoV-2 utilizes HSPG (heparin sulfated peptide glycan) for entry into the host cell (Zhang et al., 2020). In order to understand the binding mechanism, Mycroft-West and his co-workers explored and reported the tight binding between S1 RBD and heparin using molecular modeling studies (Courtney et al., 2020). Furthermore, Liu and his coworkers identified a common octasaccharide composed of IdoA2S-GlcNS6S that inhibits the spike—heparin interaction with an IC_50_ value of 38 nM (Liu et al., 2020).

**FIGURE 11:**
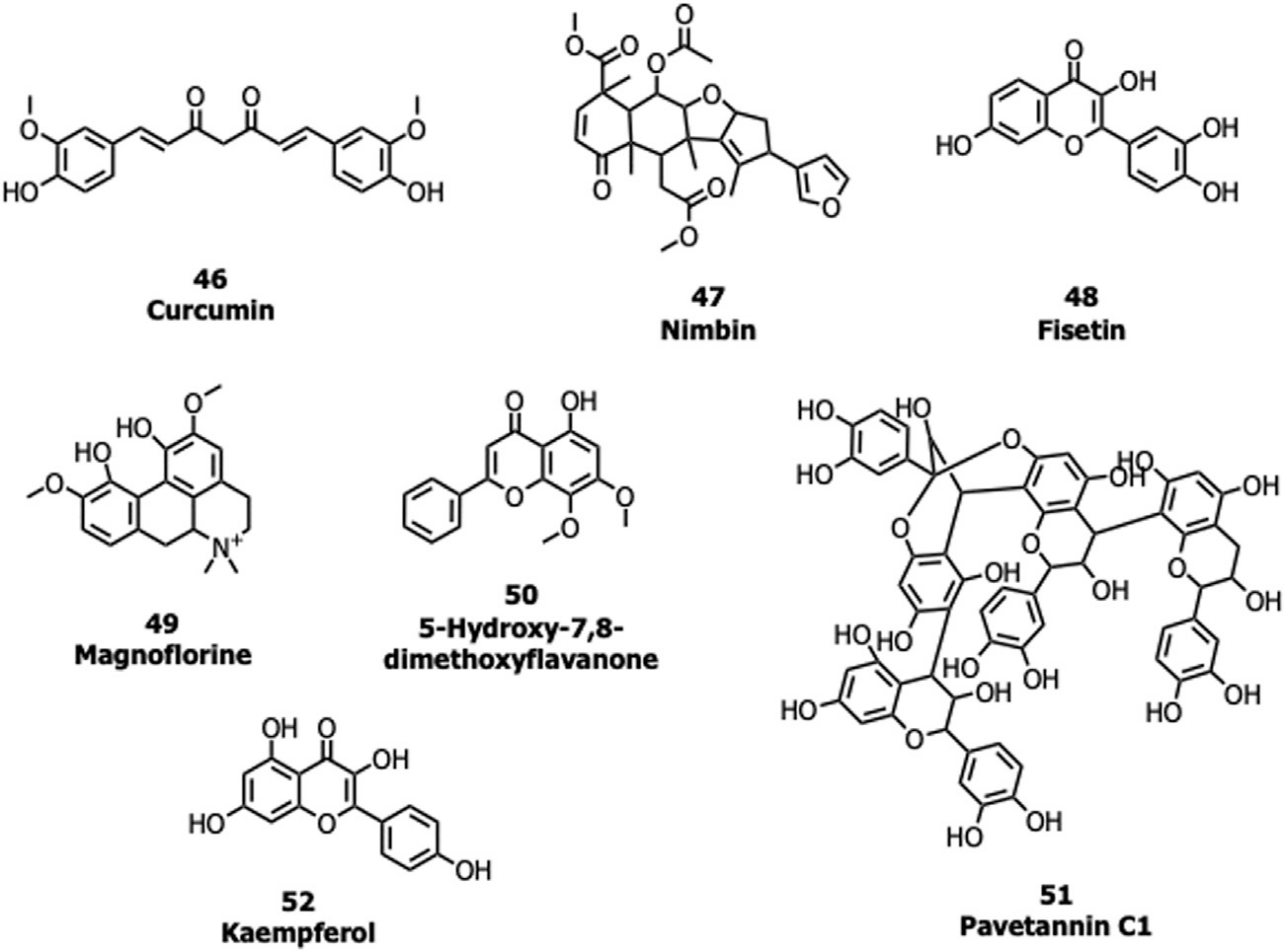
Natural products targeting the S protein.

Many natural products possessing immunomodulatory properties and antiviral activity such as curcumin (46), nimbin (47), fisetin (48) withaferin A, andrographolide, and flavonoids/non-flavonoids were screened against the S protein of SARS-CoV-2 (Vimal K. Maurya et al., 2020; Pandey et al., 2020). An Indian Official Siddha Formulation termed Kabasura Kudineer Chooranam and JACOM (patented formulation) possessing (Kiran et al., 2020) 37 active constituents such as magnoflorine (49), 5-Hydroxy-7,8-dimethoxyflavanone (50), vasicinone, quercetin, and luteolin, etc., were also subjected to docking studies against the S protein. Twenty-three different saikosaponins (Sinha et al., 2020) and 48 active compounds from all cinnamon varieties including pavetannin C1 (51) and kaempferol (52) (Prasanth et al., 2020) were screened against the S protein. Though many of these compounds had shown good binding efficacy with the S protein, further lab biological experiments are required to prove their potency and mechanism of action (Figure 11).

Griffthin (GRFT) is a carbohydrate-binding protein consisting of 121 amino acids (12.7 kDa), and inhibits viral entry by binding with the S protein (O’Keefe et al., 2010). GRFT reduced the percentage of cells killed by SARS-CoV with an EC_50_ = 48 nM. Urtica dioica agglutinin, a small plant monomeric lectin inhibits SARS-CoV S protein with an IC_50_ value of 0.53 μg/ml (Kumaki et al., 2011).

## Conclusion and Future Perspective

SARS-CoV-2 is one of the most highly pathogenic and contagious human coronaviruses next to SARS-CoV-1 and MERS-CoV posing threat to human life globally. This review about the spike protein focused on the structural information, binding mechanism of the spike along with a special emphasis on the S1 and S2 domains of SARS-CoV including the vaccines and inhibitors currently under development. SARS-CoV-2 and SARS-CoV-1 share similar structural features in their spike proteins with about 74% similarity in RBD, but differ in “RRAR furin recognition site.” The difference in sequence, binding pattern, and binding affinity of SARS-CoV-2 in comparison to SAR-CoV-1 with the host cell receptor ACE2 makes the drug development process more tedious. The structural uniqueness in the spike protein has led the focus of the drug discovery process toward the development of vaccines targeting the full-length S protein, RBD-sc dimers, mRNA, human monoclonal antibodies, and potential drug candidates including small molecule inhibitors, S domain inhibitors, ACE2 inhibitors, proteolytic inhibitors, glucose regulation protein 78 inhibitors, peptide-based inhibitors, and natural product inhibitors.

By the end of June 2020, mutations with the spike protein at the 614th amino acid position were identified due to an alteration in the single-nucleotide of the RNA code (D614G mutation), and further mutations in the sequence were found to be more transmissible (Korber et al., 2020; Yurkovetskiy et al., 2020). High numbers of genomic sequence availability and high-resolution structural information provides an opportunity to analyze the evolutionary pathway and reveal the functional basis of the mutation at the molecular level. With the available data, integrating evolutionary and structural analysis with advanced computational techniques such as artificial intelligence provides important functional information of the mutations in SARS-CoVs and will help to combat the current pandemic situation (Garvin et al., 2020).

Although the current pandemic situation has forced scientists to develop a vaccine in a very short period of time, there is a revolution in the vaccine development process. The researchers are successful with the development of a vaccine based on mRNA encoding the spike protein, with successful examples from Moderna Therapeutics and BioNtech. These vaccines are currently under Phase III clinical trials and have been approved in a few countries on the basis of emergency conditions. In addition to mRNA, an epitope can also be used to develop a vaccine as it can stimulate immune responses using isolated B cells or T cells. These preliminary success stories give us an indication that targeting the spike protein would be more advantageous in rapid drug discovery for SARS-CoV-2. Despite great efforts in the development of vaccines for HIV, HBV, and HCV, small molecule therapeutics have proven more effective for treatment. In the current pandemic situation, where SARS-CoV-2 has affected a large number of the population, an effective approach would be to attack the virus from every possible angle.

The application of repurposing strategies with known antivirals show beneficial effects in certain studies, to date there is no systemic treatment for SARS-CoV-2. Targeting small molecule inhibitors including natural inhibitors could possibly inhibit viral replication. Even after the identification of highly potent inhibitors, pharmacokinetic and toxicity studies must be cleared for the candidate molecules in order to get the approval as a drug. The focus on the natural product drug discovery could possibly reduce the toxicity issues related to small molecule inhibitors. With the available information, it is very clear that future discoveries could aim at targeting the spike protein, thereby identifying the capability of the phenotypic changes and act on designing effective candidates in the prevention and transmission of the virus.
