# Supplementary material for: An Overview of Spike Surface Glycoprotein in Severe Acute Respiratory Syndrome–Coronavirus
Source: Front Mol Biosci. 2021 Mar 16;8:637550. doi: 10.3389/fmolb.2021.637550 (PMC8058706; doi:10.3389/fmolb.2021.637550)
Supplement: Supplementary file 1 [file DataSheet1.PDF]

An Overview of Spike Surface Glycoprotein in Severe Acute Respiratory Syndrome–Coronavirus

Muthu Kumaradoss Kathiravan[1,2#], Srimathi Radhakrishnan[1,2#], Vigneshwaran Namasivayam[3*], Senthilkumar Palaniappan[4*]

[1]Department of Pharmaceutical Chemistry, SRM College of Pharmacy, SRMIST, Kattankulathur, Kancheepuram 603 203. Tamil Nadu. India.

[2]Dr. APJ Abdul Kalam Research Lab, SRM College of Pharmacy, SRMIST, Kattankulathur, Kancheepuram 603 203. Tamil Nadu. India.

[3]Pharmaceutical Institute, University of Bonn, 53121 Bonn, Germany. vnamasiv@uni-bonn.de

[4]Faculty of Pharmacy, Karpagam Academy of Higher Education, Pollachi Main Road, Eachanari Post, Coimbatore 641 021. Tamil Nadu. India. drsenthilkumar.p@kahedu.edu.in.

[#]These authors contributed equally to this work

[*]Corresponding authors

Address for Correspondence:

Dr. Senthikumar Palaniappan
Email: drsenthilkumar.p@kahedu.edu.in
Phone: +91 7373614404

Abstract

The origin of the novel coronavirus by December 2019 marked its existence in the province of the city Hubei, P.R. China. This contagious disease named as COVID-19 resulted in a massive expansion within six months by spreading to more than 213 countries. Despite the ~~available~~availability of antiviral drugs for the treatment of various viral infections, it was concluded by the WHO that there is no medicine till date to treat novel CoV, SARS-CoV-2. By the 21st century, it is identified that SARS-COV-2 is the most highly virulent human coronavirus and occupies the third position following SARS, MERS CoVs, with the highest mortality rate. The genetic assembly of SARS-CoV-2 is segmented into structural and non-structural proteins, of which two-third of the viral genome encodes non-structural proteins and the remaining genome encodes structural proteins. The most predominant structural proteins that make the SARS-CoV-2 include spike surface glycoprotein (S), membrane protein (M), envelope protein (E), and nucleocapsid protein (N). This review will focus on one of the four major structural proteins in the CoV assembly, the spike, which is involved in the host cell recognition, fusion process and is one of the promising targets. The monomer disintegrates into S1 and S2 subunits with the S1 domain necessitating binding of the virus to its host cell receptor and the S2 domain mediating the viral fusion. On viral infection by the host, the S protein is further cleaved by the protease enzyme to two major subdomains S1/S2. Spike is~~ also~~ proved to be an interesting target for developing vaccines and in particular, RBD-single chain dimer has shown initial success. The availability of small molecules and peptidic inhibitors for the host cell receptors is discussed in brief. The development of new molecules and therapeutic druggable targets for SARS-CoV-2 is of global importance. Attacking the virus employing multiple targets and strategies is the best way to inhibit the virus. This article will appeal to the researchers in understanding the structural and biological aspects of S protein in the field of drug design and discovery.

Coronaviruses (CoVs) are pathogens from the family, Coronaviridae and have an impact on human and animal health in particular their respiratory and gastrointestinal tract system that range from mild to lethal (Ghosh et al., 2020). The International Committee on Taxonomy of Viruses (ICTV) classifies coronaviruses into Coronaviridae *family* and Nidovirales *order* which is further subdivided into two subfamilies as *Torovirinae* and *Coronavirinae* (**Figure 1**) (Pal et al., 2020).

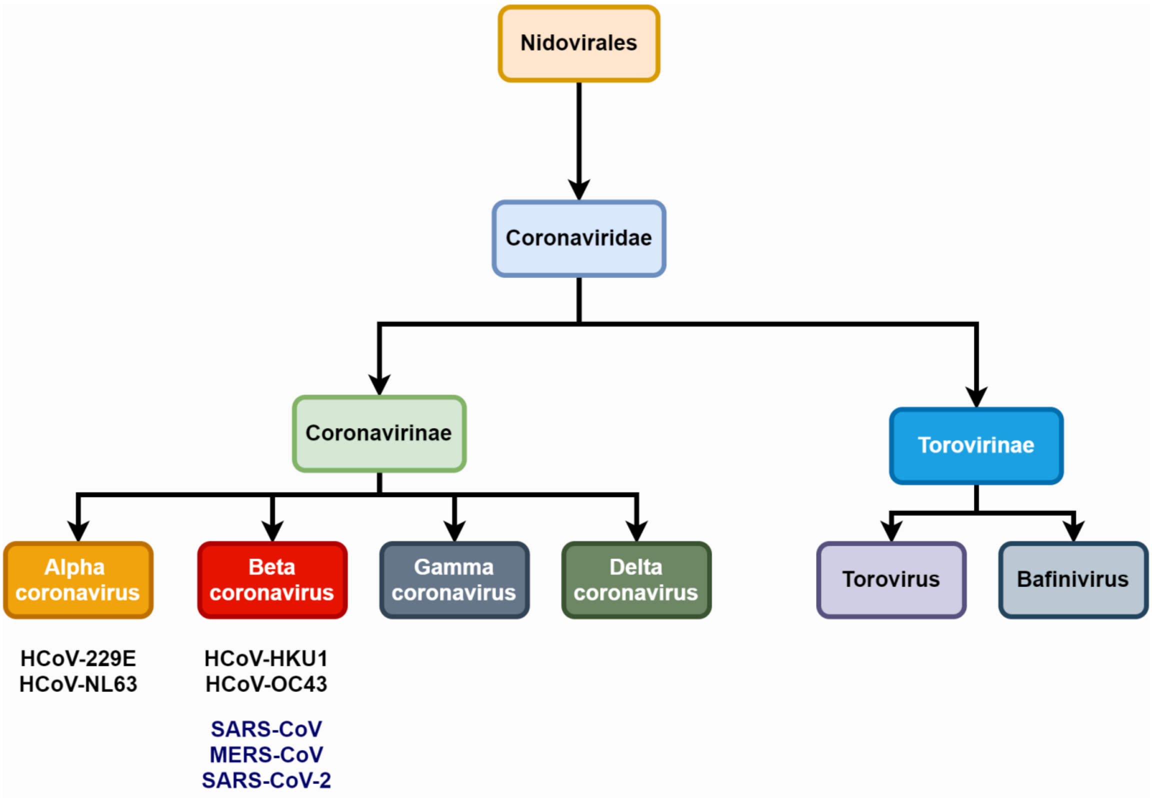

Figure 1. Schematic representation of Coronaviridae taxonomy

The CoVs are large and enveloped positive-strand RNA virus, further being subdivided into α-, β-, γ-, and δ-_CoVs. Among the four subtypes, α- and β- CoVs are identified to infect humans. Till now six human-CoVs (HCoV 229E, NL63, OC43, HKU1, SARS, and MERS) have been reported globally. **Table 1** illustrates the classification of coronavirus, variants, and their host organism. ~~From~~Among these six human-CoVs, SARS- and MERS- CoVs are

extremely pathogenic and the transmission ~~among~~within humans generally occurs through close contacts ~~via~~through inhalation of respiratory droplets ~~inhaled~~ or ~~sneezed~~sneeze similar to influenza and other respiratory pathogens (Ghosh et al., 2020; Pillaiyar et al., 2016). The remaining four CoVs cause mild respiratory infections leading to the common cold. At the end of 2002, the outbreak of SARS in Guangdong province in China registered 8098 cases with 774 deaths. Almost a decade has been passed since the outbreak of SARS-CoV, the subsequent zoonotic coronavirus MERS-CoVs have emerged in Saudi Arabia with 2494 cases and 858 deaths (Source: WHO). At the end of ~~the last year~~ 2019, another new strain of coronavirus 2019-nCoV has been found among the people reported for the recent ongoing pneumonia outbreak in the city of Wuhan in China. Till now (as of December 02, 2020, WHO) the 2019-nCoV had spread rapidly in over 220 countries and registered over 63,360,234 reported cases as affected and 1,475,825 deaths (https://www.who.int/emergencies/diseases/novel-coronavirus-2019).

Table 1. Classification of different types of CoVs with their variants name, year of discovery, and ~~the~~ host organism of the CoVs

**Type of CoV**	**Coronaviruses**	**Discovery**	**Natural Host(s)**
α-Coronaviruses	HCoV-229E	1966	Bats
	HCoV-NL63	2004	Palm civets, bats
β-Coronaviruses	HCoV-OC43	1967	Cattle
	SARS-CoV-1	2003	Palm civets
	HCoV-HKU1	2005	Mice
	MERS-CoV	2012	Bats, camels
	SARS-CoV-2	2019	Bats
Non-human	BCoV	1890	Cattle
	TGEV	1946	Pigs
	MHV	1949	Mice
	FIPV	1963	Cats
	CCoV	1971	Dogs
	PEDV	2013	Pigs

1. Coronavirus replication cycle

CoVs ~~contains~~contain a non-segmented single-stranded RNA featuring the largest viral RNA genomes reported so far and ranging approximately 26-32 kilobase (kb) genomes. CoVs are lipid enveloped and spherical in shape with a size of approximately 100–120 nM (Ghosh et al., 2020). SAR-CoV-2 belongs to the beta-corona virus class comprising of ~30 kb in length and the replication cycle is shown in **Figure 2**.

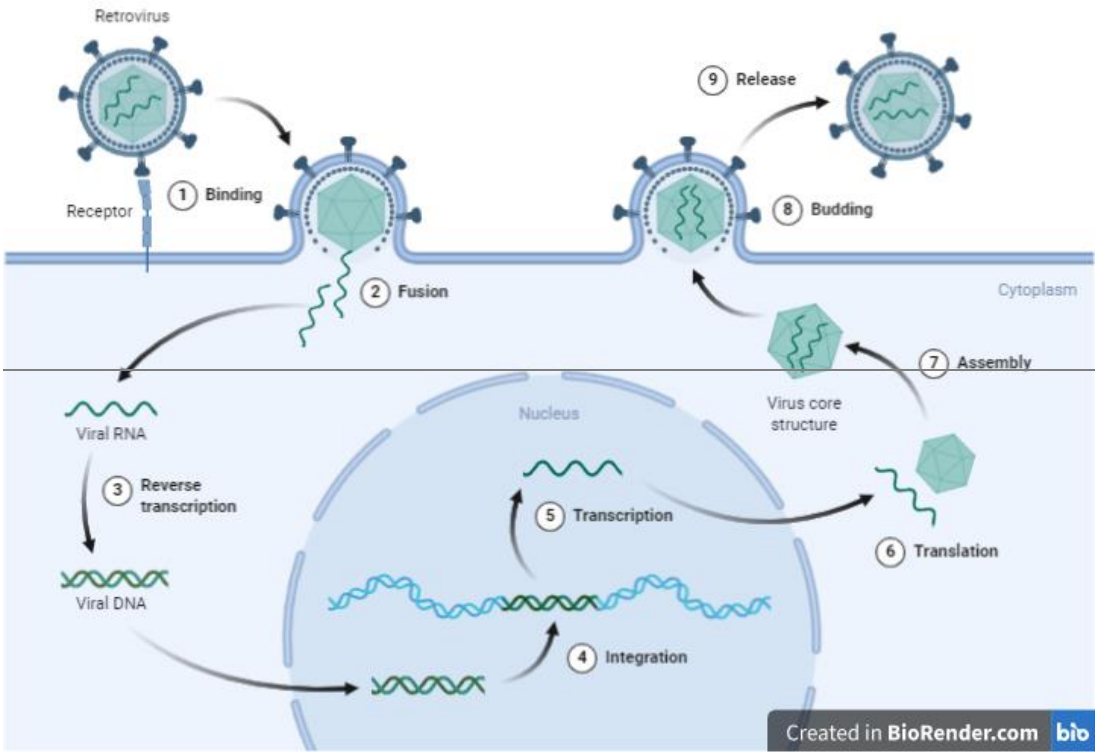

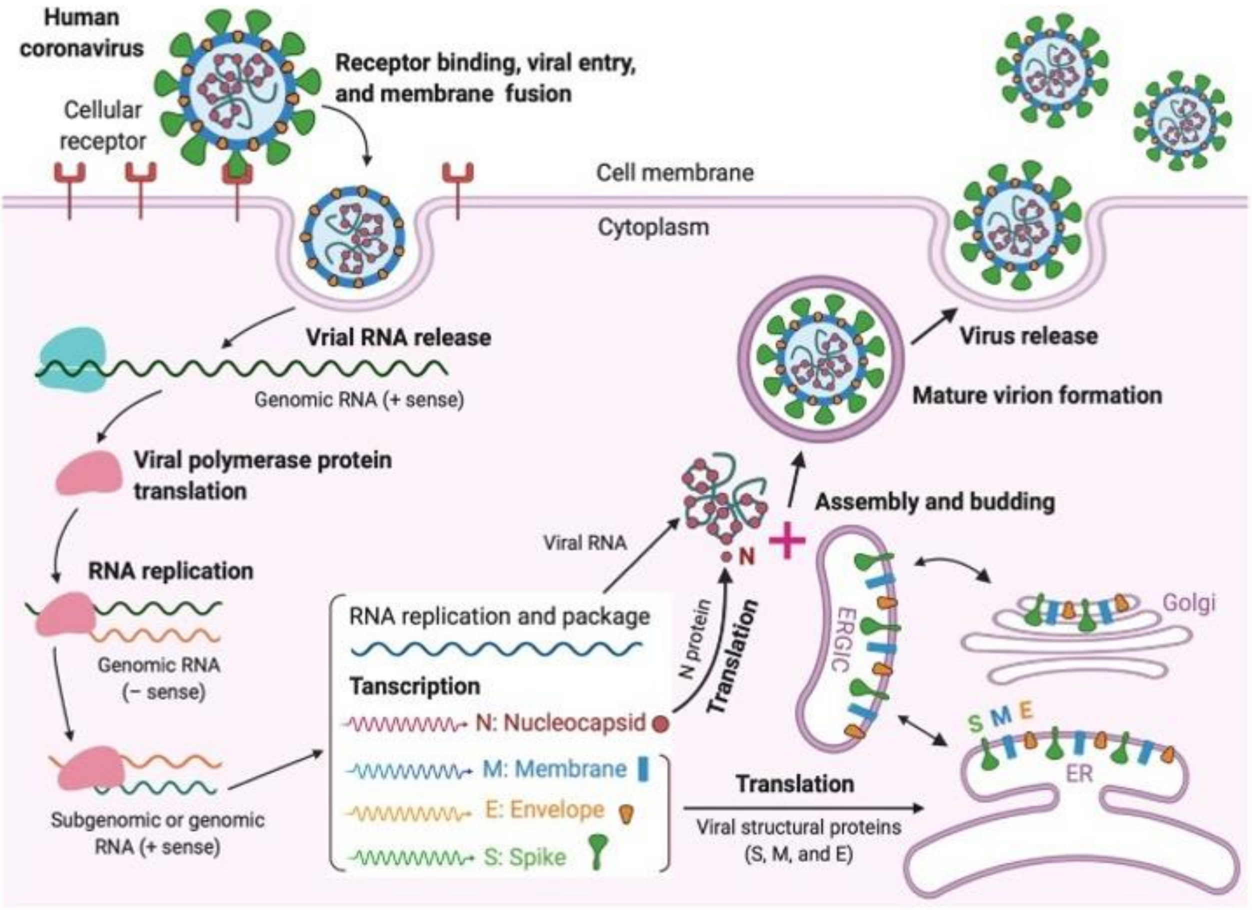

Figure 2. Coronavirus replication cycle ~~(Source: https://www.biorender.com)~~[(Jiang et al., 2020) Copyright ©2020 Elsevier Inc., based on the reuse-provisions of Elsevier's COVID-19 Resource Centre]

Similar to other CoV neighbors, SARS-CoV-2 utilizes the host cell-machinery for replication which involves various viral structural and non-structural proteins. Coronavirus particles consist of four main structural proteins namely the spike (S), membrane (M), envelope (E), and nucleocapsid (N) proteins. Briefly, a fully mature viral particle starts its journey with host cell membrane fusion or endocytosis process. Based on the host cell fusion mechanism, binding of the receptor-binding domain (RBD) of the spike (S) protein to the host receptor such as angiotensin-converting enzyme-2 (ACE2), dipepitdylpeptidase IV (DDPIV) changes the RBD conformation which leads to the merging of the viral membrane with the host

membrane. With the fusion process, the viral genetic material (single-stranded RNA) is injected into the cytoplasm for the host cell ribosome dependent-translation process in which ORF1ab is translated into viral polyproteins (e.g., pp1a, pp1b, etc.). Subsequently, various non-structural proteins, including RNA-dependent RNA polymerase and helicase, are produced from the pp1a and pp1b using the protease enzymes (e.g., PL^{pro} and $3CL^{pro}$). Non-structural proteins are involved in the viral transcription and replication process. Several copies of original viral RNA synthesized by RNA polymerase are now transcribed into full-length mRNA negative-strand template for the translation process in which structural proteins are produced in the endoplasmic reticulum. Ultimately, all structural proteins and genomic RNA are compiled to form the virion, which is translocated into Golgi, where the virions are released out of the cell by transporting through vesicles. To inhibit the virus progression, several key steps have been identified in the virus life cycle; (i) **RBD binding** which plays an important role in the viral fusion to host cell, (ii) **protease enzymes** play a key role in synthesizing RNA-dependent RNA polymerase and (iii) **RNA-dependent RNA polymerase** for transcription. Blocking any of these crucial steps might be an attractive target for antiviral development, including drugs and vaccines (Huang et al., 2020).

3. Overview of the S protein of SARS-CoV-2

The S protein is a homotrimeric transmembrane glycoprotein fused by three monomer units. On the surface of the protein 100 crown-shaped spikes are present with ~30Kb in length and as ~~the~~a larger part among the four structural proteins M, E, and N (Song et al., 2018). The length of each spike ranges from 20-40 nm making it more stretchable to fit into the Angiotensin Converting Enzyme II (ACE2) of the host cell ~~receptors~~receptor (Zhou et al., 2020). Each monomer (~180 KDa) of the S protein consists of a total of 1273 amino acids which is divided into two major functional domains S1 and S2. Thus, the trimer of an S protein contains three S1 and S2 subunits coiled together (Yan et al., 2020). The S1 domain

can be segregated into a single peptide (SP), N-terminal domain (NTD), C-terminal domain (CTD) also called the receptor binding domain (RBD) with a loop region known as receptor binding motif (RBM). The S2 domain consists of fusion peptide (FP), heptad repeat (HR) 1 and 2, transmembrane (TM) and cytoplasmic (CP) domain (**Figure 3**). S1 domain is responsible for the recognition of ACE2 and S2 mediates membrane fusion into the host cell. The sequence motif "KRSFIEDLLFNKV" is responsible for the initial binding of SARS-CoV to lung cells and activates the S protein by proteolytic cleavage. (Robson et al., 2020).

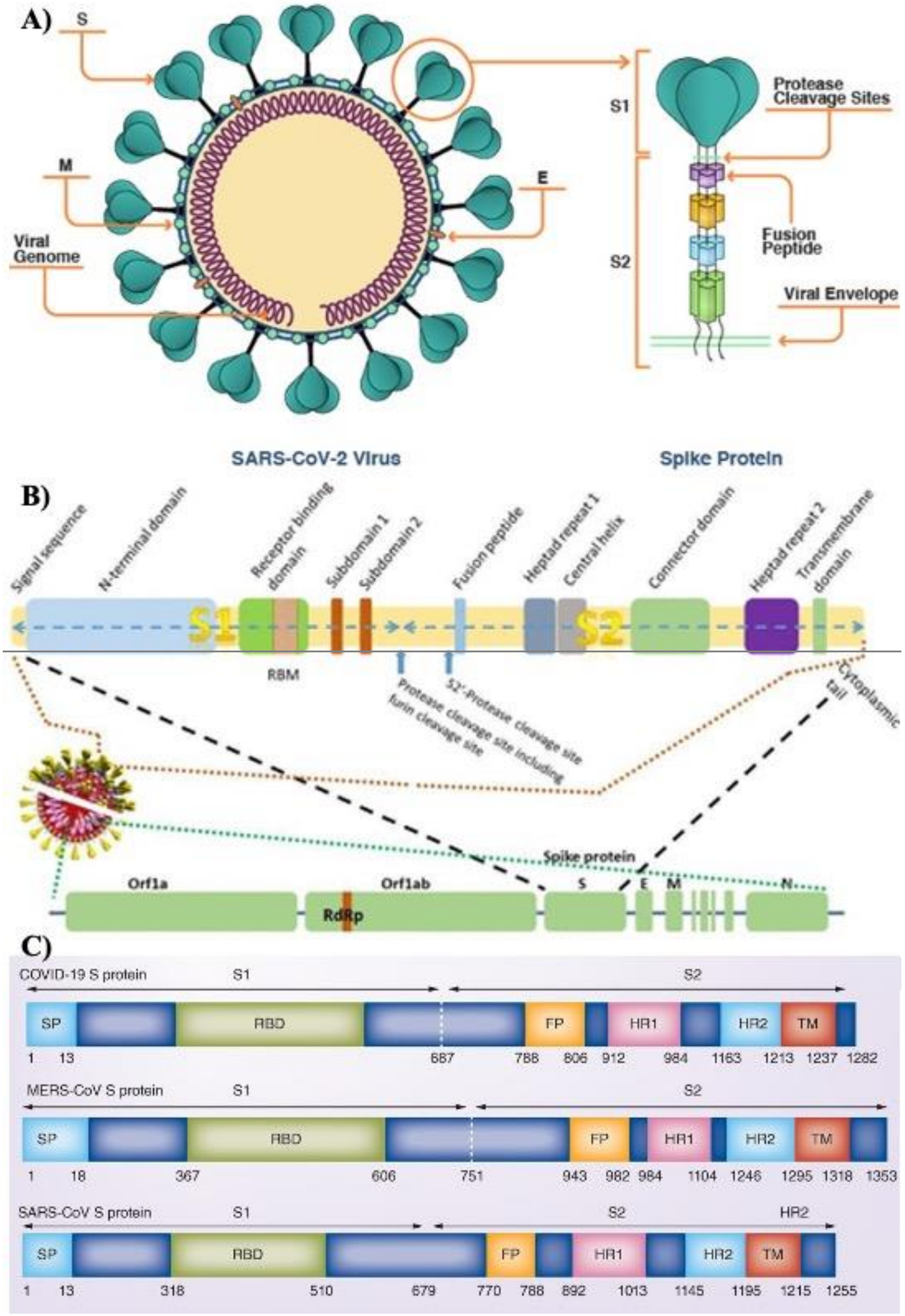

A)
S
M
E
Viral Genome
S1
S2
Protease Cleavage Sites
Fusion Peptide
Viral Envelope
SARS-CoV-2 Virus
Spike Protein
B)
Signal sequence
N-terminal domain
Receptor binding domain
Subdomain 1
Subdomain 2
Fusion peptide
Heptad repeat 1
Central helix
Connector domain
Heptad repeat 2
Transmembrane domain
S1
S2
RBM
Protease cleavage site including furin cleavage site
S2'-Protease cleavage site
Cytoplasmic tail
Spike protein
Orf1a
Orf1ab
RdRp
S
E
M
N
C)
COVID-19 S protein
S1
S2
SP
RBD
FP
HR1
HR2
TM
1 13 687 788 806 912 984 1163 1213 1237 1282
MERS-CoV S protein
S1
S2
SP
RBD
FP
HR1
HR2
TM
1 18 367 606 751 943 982 984 1104 1246 1295 1318 1353
SARS-CoV S protein
S1
S2
HR2
SP
RBD
FP
HR1
HR2
TM
1 13 318 510 679 770 788 892 1013 1145 1195 1215 1255

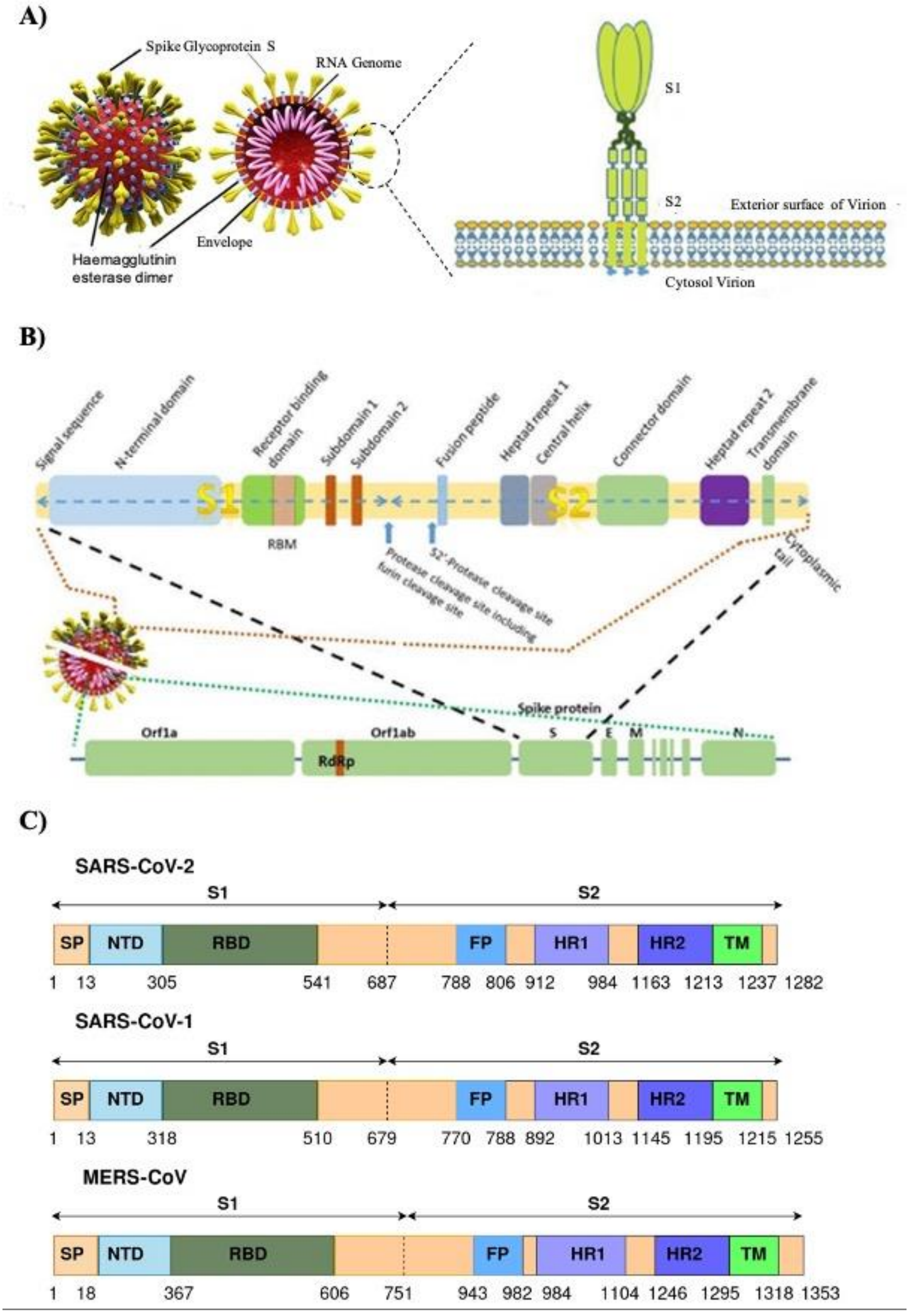
A)
Spike Glycoprotein S
RNA Genome
Envelope
Haemagglutinin esterase dimer
S1
S2
Exterior surface of Virion
Cytosol Virion
B)
Signal sequence
N-terminal domain
Receptor binding domain
Subdomain 1
Subdomain 2
Fusion peptide
Heptad repeat 1
Central helix
Connector domain
Heptad repeat 2
Transmembrane domain
S1
S2
RBM
Protease cleavage site including furin cleavage site
S2'-Protease cleavage site
Cytoplasmic tail
Spike protein
Orf1a
Orf1ab
RdRp
S
E
M
N
C)
SARS-CoV-2
S1
S2
SP NTD RBD FP HR1 HR2 TM
1 13 305 541 687 788 806 912 984 1163 1213 1237 1282
SARS-CoV-1
S1
S2
SP NTD RBD FP HR1 HR2 TM
1 13 318 510 679 770 788 892 1013 1145 1195 1215 1255
MERS-CoV
S1
S2
SP NTD RBD FP HR1 HR2 TM
1 18 367 606 751 943 982 984 1104 1246 1295 1318 1353

Figure 3. **A)** TheA representative of SARS CoV-2 and the S protein with their different binding domains S1 and S2 (https://www.cas.org/blog/covid-19-spike-protein);[(Pillay et al., 2020) Copyright ©2020 Publisher BMJ]; **B)** the different region of the S protein of SARS CoV-2 ([(Pillay et al., 2020) Copyright ©2020 Publisher BMJ] and **C)** StructuralGenomic information of S protein. and the different binding domains for SARS-CoV-2, SARS-CoV-1 and MERS-CoV (Bahrami et al., 2020).

The surface subunit S1 comprises of 672 amino acids organized into SP, NTD, and RBD. S1 subunit initiates the process of viral entry via attaching with the cell receptor. At the top of each S1 monomer, one RBD is present for interaction with ACE2. In the specified domain, RBDs undergo hinge-like conformational movementsmovement that transiently exposeexposes an open state or a closed state (McKee et.al, 2020). In particular, the extended loop region, RBM of the RBD contains the amino acid residues that bind to ACE2 (Lan et al., 2020). Comparing RBM of SARS-CoV-1 and SARS-CoV-2, the latter forms a larger binding interface and makes a large number of contacts with ACE2 with a higher binding affinity (K_d 31 and 4.7 nM, respectively) (Zhai et al., 2020).

Among the two domains S1 and S2, the sequence of S2 of SARS-CoV-2 S2 sequence shows 90% similarity with SARS-CoV-1. This suggests that the S2 domain is prone to less mutation and hence targeting the S2 domain might be useful in the preventive stage of viral infection. The shorter FP consists of 18 amino acids that play an important role in the fusion process and is responsible for the binding affinity towards the host cell. The HR1 and HR2 consist of a peptide sequence motif “HPPHCPC” representing hydrophobic (H), polar (P) and charged (C) residues. This sequence of the peptide region adopts an α-helix with a hydrophobic interface to drive the membrane fusion. Among the different variants of CoV, HR is highly

conserved, and in particular, HR2 is 100% identical in comparison to the other regions, HR1 (88%), TM (93%), and CP (97%) in the S2 domain (**Table 2**). The TM is long enough in length which anchors the S protein in the membrane, has three conserved and distinctive domains namely N-terminal tryptophan-rich and hydrophobic central region ends with cysteine-rich C-terminal domain. In the final section of the S2 domain, the CP tail has a high amount of S-acylated cysteine residues.

The SARS-CoV-2 S has 1273 amino acids and cleaves into S1 and S2 domains (**Figure 3C**). The S1 domain comprises of SP (1–13) located at the N-terminus, RBD (319–541), and RBM (437-508). The S2 subunit has 686–1273 residues with FP (788–806), HR1 (912–984), HR2 (1163–1213), TM domain (1213–1237), and cytoplasm domain (1237–1273). The SARS-CoV-2 has newly added 18 amino acids when compared with SARS-CoV. The RBD (318-510) in SARS-CoV has fewer amino acids residue when compared with SARS-CoV-2, which could be the reason for increased binding affinity towards the cellular receptor. Furthermore, there is no significant difference in amino acid residue between SARS and SARS-CoV-2 in the RBM region. The FP for both SARS and SARS-CoV-2 possess 18 conserved residues (Krishnamoorthy et al., 2020).

Table 2. Percent similarity of the various domains of S protein for SARS-CoV-2 in comparison with SARS-CoV-1

Domain	SARS CoV-1	Percent Similarity (%)
S1	Overall	64
	NTD	51
	RBD	74
	RBM	50
S2	Overall	90
	FP	93
	HR1	88
	HR2	100
	TM	93
	CP	97

4. Binding Mechanism of the S protein

The entry of coronavirus through the S protein is a combined process involving receptor-binding and proteolytic processing to promote virus penetration into the host cell (Walls et al., 2020). In the pre-fusion conformation state, the S protein exists in the non-covalently bound state. This state reveals that the binding within the S protein is less stable and can open ~~up on~~upon interaction with receptors as depicted in **Figure 4**. The RBD in S1 extends a loop to bind with the host Peptidase Domain (PD) of ACE2 through RBM. Once bound, the S2 domain undergoes structural rearrangement to activate the S protein for membrane fusion. This conformational change in the S2 domain causes the fusogenic potential to penetrate into the host cell. The heptad-repeat regions HR1 and HR2, gather into a six-helix bundle (HB) and bring ~~the~~ FP and cellular membrane in a hairpin conformation (Alnefaie et al., 2020). The affinity between HR1 and HR2 against each other stabilizes this required conformation and confirms the fusion of the virus into the cellular membrane (Guo et al., 2020). The FP along with heptad region HR1 and HR2 in the S2 domain assists viral fusion into the host cell (Xia

et al., 2020; Liu et al., 2004). The fusion between the S2 domain and ACE2 receptor allows the spike to transform from the Pre-fusion to the Post-fusion conformation. The crystal structure of SARS-CoV-2's RBD in complex with ACE2 showed that the RBD connects with the proteolytic domain (PD) of ACE2. At the N terminus Q498, T500, and N501 of the RBD interacts via H-bond with Y41, Q42, K353, and R357 from ACE2. The RBD contacts via Y453, the ACE2 PD at the residue H34. In the C terminal region, van der Waals interactions are formed between Q474 of RBD and Q24 of ACE2, F486 of RBD, and M82 of ACE2.

The amino acid Q498 recognizes ACE2 and is responsible for infecting host cells; N501 helps in the transmission from human to human. L455 helps in viral binding to the ACE2 receptor. F486 supports binding and enhances viral infection. S494 provides positive support for enhancing the binding of the virus to ACE2. Upon viral infection, the post-fusion state begins with the activation of protease enzymes such as furin and TMPRSS-2 (Hoffmann et al., 2020). The S protein of SARS-CoV-2 differs from SARS-CoV in its furin recognition site "RRAR" and is absent in other types of coronaviruses, making it a unique cleavage site (Coutard et al., 2020). This site can be an important target for inhibitors (Seidah et al., 2012). S trimer is extensively decorated with N-linked glycans that are important for proper folding and for modulating accessibility to host proteases and neutralizing antibodies. ~~Supplementary Table S1 represents the available crystal structures of S protein along with the complex.~~

The advancement in crystallographic techniques provides a greater understanding of the structural biology of proteins. With the recent revolutionary of cryo-EM, the number of protein structures is growing at a very high rate and in particular the protein which is difficult to crystallize. This is visible from the number of reported structures (129 structures) for SARS-CoV-2. Supplementary Table S1 shows the summary and the list of available crystal structures of S protein along with the complex. The high resolution (1.5 Å) structures using X-ray crystallography is available for MERS-CoV (PDB ID: 5X4R), SARS-CoV-1 (PDB ID:

1ZVA) and SARS-CoV-2 (PDB ID: 6M1V). In the current pandemic situation, the application of these techniques provides a structural understanding of CoVs which is greatly important (163 structures are reported in 2020) for drug discovery and development. The structures of spike protein from MERS, SARS-CoV-1 and -2 were determined and in particular, the Receptor binding domain (RBD), N-terminal domain (NTD), C-terminal domain (CTD) are reported predominately due to their importance in binding with the host cell receptors. The reported crystal structures with different antibodies help to form structures and understand the binding mechanism of the S-protein, the functional movements of the domains and their involvement in binding with the host cell receptor. Specifically, detailed information was obtained for SARS-CoV-2 with the ACE2 receptor.

The Open Reading Frames (ORF) ORF1a and ORF1b are translated into polyproteins pp1a (4382 amino acids) and pp1ab (7073 amino acids). These polyproteins are processed by 3-C-like protease ($3CL^{Pro}$) and papain-like protease (PL^{Pro}) to generate a variety of non-structural proteins (NSPs), including RNA-dependent RNA polymerase (RdRp) and helicase, for catalyzing viral genome replication and protein synthesis.

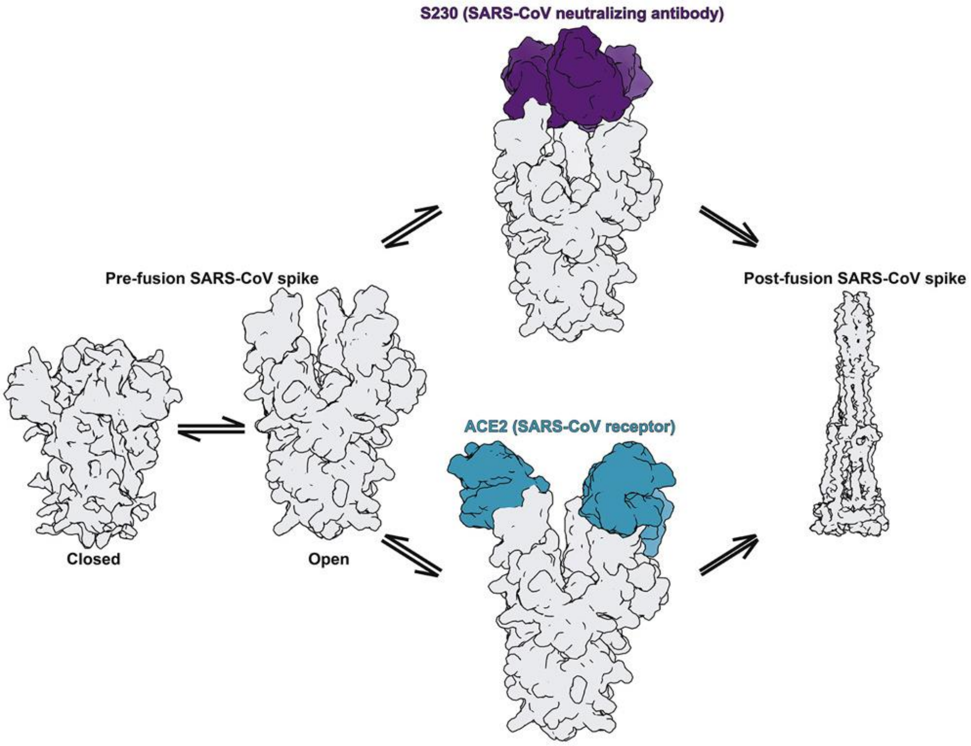

Figure 4. Fusion states in S protein elucidate the mechanism of activation ~~(~~[(Walls et al., 2019) Copyright ©2020 Elsevier Inc., based on the reuse-provisions of Elsevier's COVID-19 Resource Centre].

5. Targeting S protein

5.1. Vaccines

Since the first outbreak of SARS-CoV-1 in 2002, there is active involvement in the development of vaccines against coronaviruses (Modjarrad et al., 2016; Jiang et al., 2012; Wang et al., 2020; Zhou et al., 2019; Dai et al., 2020). The recent outbreak of SARS-CoV-2 affected ~~a~~ large number of people and streamed larger efforts in the development of vaccines. As of November 2020, WHO has not recommended any vaccines for SARS-CoV-2, but few of the vaccines are closer to approval in selected countries. The vaccine development targeting S protein can be grouped as full-length S protein, RBD, and RNA.

a) Full-length S-protein

A great interest and focus are on developing vaccines targeting the full-length S-protein of SARS-CoV. The vaccine for full-length S protein showed required immunity against SARS-CoV-1 suppressing the viral proliferation but resulted in harmful immune response (Jiang et al., 2005). The vaccine-induced antibodies against SARS-CoV-2 bind with the virus. In this, neutralizing antibodies provides efficient blockade for viral infection and non-neutralizing antibodies generate antibody-dependent enhancement effect that can aggravate the infection (Garber et al., 2020; Iwasaki and Yang et al., 2020; Tetro et al., 2020; Ulrich et al., 2020). The studies in MERS-CoV neutralizing single-domain antibodies (sdAbs) from immunized dromedary camels and lamas showed EC_{50} values between 0.001 - 0.003 μg/mL and low K_d values in the range 0.1 to 1 nM (Seidah et al., 2012). Furthermore, the sdAbs showed EC_{50} of 0.0009 - 0.07 μg/mL and 0.13 - 0.51 μg/mL against SARS-CoV-2 pseudotypes, and authentic SARS-CoV-2, respectively (Chi et al., 2020). Liu L et al. identified that anti S-protein immunoglobulin (IgG) on administration in healthy macaque with SARS-CoV infection ~~resulted~~, resulting in severe acute lung injury due to antibody-dependent enhancement (ADE) induced by peptide 597-603 of S protein (Liu et al., 2019; Wang et al., 2016). Further identifying the antibodies that cause infections and avoiding ADE has to be considered in the vaccine development targeting full-length S protein.

b) RBD-sc DIMERS in vaccine development:

Due to the drawbacks of the full-length S protein vaccine, the focus shifted to the RBD region for the vaccine candidate. The Antigenic epitopes from the RBD of SARS-CoV neutralize the antibodies as well as the CD8+ T cell responses. The RBD-dimer vaccine significantly increased neutralizing antibody since it exposed dual receptor-binding motifs and protected mice against MERS-CoV infection better than RBD-monomer (Dai et al.,

2020). This strategy has led to the design of a vaccine for SARS-CoVs with 10-100 fold enhancement of neutralizing antibodies. A recent study on vaccine based on RBD against SARS and MERS have shown good efficacy (Modjarrad et al., 2016; Jiang et al., 2012; Wang et al., 2020; Dai et al., 2020) but have few limitations including low immunogenicity, protein sequences, and fragment lengths. The RBD vaccine generates potent antibodies and provides sustained protection when compared with the full-length S protein vaccine (Yang et al., 2020). Recombinant RBD protein-based vaccine is also equally effective but needs repeated dosing.

The epitope can be used to develop a vaccine as it can stimulate immune responses using isolated B-cell or T-cells and the use of multiple epitopes can further improve vaccine efficacy. Recently five epitopes were identified through literature mining located in the fully exposed RBD hotspot regions of the S protein possessing antigenicity include three B-cell epitopes (‘RQIAPGQTGKIADYNYKLPD’, ‘SYGFQPTNGVGYQ’ and ‘YAWNRKRISNCVA’), and two T-cell epitopes (‘KPFERDISTEIYQ’ and ‘NYNYLYRLFR’). (Li et al., 2020). All five epitopes were found to be non-toxic and have the potential to be developed as a vaccine candidate.

c) mRNA vaccine:

In the epidemic of SARS-CoV-2, the development of mRNA vaccines has gained huge interest. The flexibility in the design of the RNA vaccine makes it more advantageous during the pandemic. The RNA vaccine is well tolerated by the human body and is considered to be safe. In an RNA vaccine, the genetic information for the antigen is delivered generally through a lipid nanoparticle. Currently, many mRNA ~~viruses~~vaccines are under development other than SARS-CoVs like Zika and cytomegalovirus. Among the 51 vaccines in clinical trials for SARS-CoVs, six of the vaccines are based on RNA (https://www.who.int).

The vaccine mRNA-1273 developed by Moderna Therapeutics in collaboration with the National Institute of Allergy and Infectious Disease Vaccine Research Center (NIAID VRC) is based on mRNA that encodes for a full-length, prefusion stabilized S protein of SARS-CoV-2 encapsulated by novel lipid nanoparticle. The mRNA-1273 vaccine is currently in Phase III and is in the process of approval in selected countries.

Another mRNA-based vaccine candidate is BNT162b2 which encodes a full-length S protein with two stabilizing proline residues developed by BioNTechin in collaboration with Fosun Pharma and Pfizer. This vaccine is in Phase III clinical trials. BNT162b2 was found to be 95% effective against SARS-CoV-2 after 28 days of the first dose and showed a good safety profile (Mulligan et al., 2020). European Medical Agency has received the application for conditional marketing authorization for BNT162b2.

Furthermore, CVnCoV vaccine developed by CureVac is under Phase II, Lunar-COV19 vaccine by Arcturus/Duke-NUS in Phase I/II, and two vaccines from Imperial College London and People's Liberation Army (PLA) Academy of Military Sciences/Walvax Biotech are in Phase I clinical trials (c.f. **Table 3**). Still the current vaccines are in the developmental phase and the process of approval is unclear about issues including bulk production, stability, storage, and mucosal immunity upon injection (Krammer et al., 2020).

Table 3. List of mRNA vaccines in various stages of clinical trials

Vaccine name	Company name	Clinical Status
mRNA-1273	Moderna / NIAID	Phase III
BNT162b2	BionTech / Fosun Pharma / Pfizer	Phase III
CVnCoV	CureVac	Phase II
LUNAR-COV19	Arcturus / Duke-NUS	Phase I/II
LNP-nCoVsaRNA	Imperial College London	Phase I
ARCoV	People's Liberation Army Academy of Military Sciences / Walvax Biotech	Phase I

5.2. Human monoclonal antibody targeting RBD in vaccine development

Targeting only the RBD reduces the levels of antibody titer thereby making it a safe and efficacious target. Tian et al. revealed that the most potent SARS-CoV-specific neutralizing antibodies (e.g., m396, CR3014, CR30222) targeting the ACE2 binding site of SARS-CoV failed to bind SARS-CoV-2 S protein (Tian et al., 2020). This indicates that changes in the amino acid could have caused the exacerbation of antibodies. The effect on cross-neutralizing antibodies is to be further studied to target RBD in the development of vaccines.

5.3. Inhibitors

5.3.1. Small molecule inhibitors

SARS-CoV-2 transfers into the human cell by first binding ~~with~~ the spike of S protein with the host cell receptors. The S protein of SARS-CoV-2 shows an 80% similarity with SARS-CoV-1 and 96% similarity with bat-CoV RaTG13 (Zhou et al., 2020). Zhang et al. also revealed that the genome sequence of SARS-CoV-2 has 89.1% similarity towards SARS-like coronaviruses (Wu et al., 2020). SARS-CoV-2 uses the ACE2 receptor for entry into the host cell similar to SARS-CoV-1 (Zhou et al., 2020). The RBD from SARS-CoV-2 and SARS-CoV-1 interacts with ACE2 is found to have 74% similarity (Yan et al., 2020). The plasmin resonance spectrometry uncovered that the RBD of the spike of SARS-CoV-2 has a high affinity (K_d = 14.7 nM) for the ACE2 receptor of the host cell (Wrapp et al., 2020).

ACE is a central component of the renin-angiotensin system and controls blood pressure. It is a highly glycosylated type I integral membrane protein and converts angiotensin I to angiotensin II. Though ACE1 and ACE2 both cleave the peptide, there is a significant difference in their mechanism of function. Angiotensin (Ang) I (a decapeptide) is converted into Ang II (an octapeptide) by ACE1. This involves dipeptide His-Leu from Ang I to form

Ang II. This process is responsible for vaso- and broncho-constriction, increased vascular permeability, inflammation, fibrosis, and thereby causing acute respiratory distress syndrome (ARDS) and lung failure (Yang et al., 2014).

CoVs use two receptor binding pathways viz., clathrin (endosomal) and non-clathrin pathways (non-endosomal) (Inoue et al., 2007; Wang et al., 2008). In the clathrin pathway, the S protein of the CoV binds to the host receptor and embodies vesicles that mature to late endosomes. These endosomes get acidified and stimulate the H^+-dependent activation of cellular cathepsin L proteinase in late endosomes and lysosomes, cleaving and activating the S protein which initiates viral fusion. SARS-CoV-2 also uses host cell receptor CD147 along with ACE2 for entry into the host cell (Wang et al., 2020). In the non-clathrin pathway, membrane fusion is the critical stage in the CoV life cycle. The membrane fusion is activated by cleavage of the host proteases include Cathepsin L, TMPRSS2, and TMPRSS1 1D (airway trypsin-like protease) at S1/S2 cleavage site (Shirato et al., 2013). These proteases are also an attractive target for SARS-CoV-2 (Zhou et al., 2015). Recently, the significance of TMPRSS2 in the life cycle of SARS-CoV-2-infected VeroE6 cells was confirmed (Geller et al., 2012). **Table 4** illustrates the cellular receptors in the coronaviruses.

Table 4. Classification and cellular receptor of the coronaviruses

HCoV genera	Coronaviruses	Cellular receptor
α-Coronaviruses	HCoV-229E	Human aminopeptidase N (CD13)
	HCoV-NL63	ACE2
β-Coronaviruses	HCoV-OC43	9-*O*-Acetylated sialic acid
	HCoV-HKU1	9-*O*-Acetylated sialic acid
	SARS-CoV-1	ACE2
	MERS-CoV	DPP4
	SARS-CoV-2	ACE2

a) S-domain inhibitors

Adedeji et al. screened a chemical library of 3000 molecules for SARS-CoV-1 entry inhibitor and identified oxazole-carboxamide derivative (**1**) as a lead molecule that interferes with the RBD blocking the ACE2 recognition. Compound **1** showed inhibition with an EC_{50} value of 3.1 µM and a 50% cytotoxic concentration (CC_{50}) value of >100 µM, but it doesn't affect the expression levels of ACE2 (Adedeji et al., 2013).

Yi and his coworkers identified two molecules, TGG (**2**) and Luteolin (**3**) inhibiting the viral entry into Vero E6 cells by binding with the S2 protein of SARS-CoV-1 (Yi et al, 2004). Compounds **2** and **3** exhibited EC_{50} of 4.5 µM and 10.6 µM, respectively, and CC_{50} of 1.08 mM and 0.155 mM with a selectivity index (SI) of 240.0 and 14.62, respectively. The compounds **2** and **3** were safe up to 232.2 mg/kg and 456 mg/kg, respectively in the LD_{50} acute toxicity study. An analog of compound **3**, Quercetin (**4**) showed inhibitory activity at EC_{50} = 83.4 µM and CC_{50} = 3.32 mM (Yi et al, 2004).

A small-molecule HIV entry inhibitor, ADS-J1 (**5**) inhibits >90% of MERS-CoV pseudovirus infection in NBL-7 and Huh-7 cells at a concentration of 20 µM (Zhao et al., 2013). ADS-J1 inhibits the entry of pseudotyped MERS-CoV (EC_{50} = 0.6 µM) in the DPP4-expressing cell line and CC_{50} with 26.9 µM in NBL-7 and Huh-7 cells by MTT assay by forming a six-helix bundle and interrupting the interactions between HR1 and HR2 of MERS-CoV. Chu et al. identified that ADS-J1 (**5**) also possesses potential inhibitory activity against SARS-CoV-1 viral entry (EC_{50} = 3.89 µM) (Chu et al., 2008).

An abelson kinase inhibitor, Imatinib (**6**) inhibits S protein-induced fusion of coronavirus including SARS-CoV, MERS-CoV, and infectious bronchitis virus (IBV) at 10 µM and without cytotoxic effects in Vero cells up to concentrations of 100 µM (Sisk et al., 2018; Coleman et al., 2016).

Lundin et al. screened a library of 16,671 ~~compounds with a~~ diverse set of molecules and identified a small molecule inhibitor, K22 (**7**), inhibiting HCoV-229E with an IC_{50} value of

0.7 μM. K22 targets the initial stage in the life cycle of HCoV-229E and possibly interacts with viral particles and ~~resulted~~results in the inactivation state of the virus (Lundin et al., 2014).

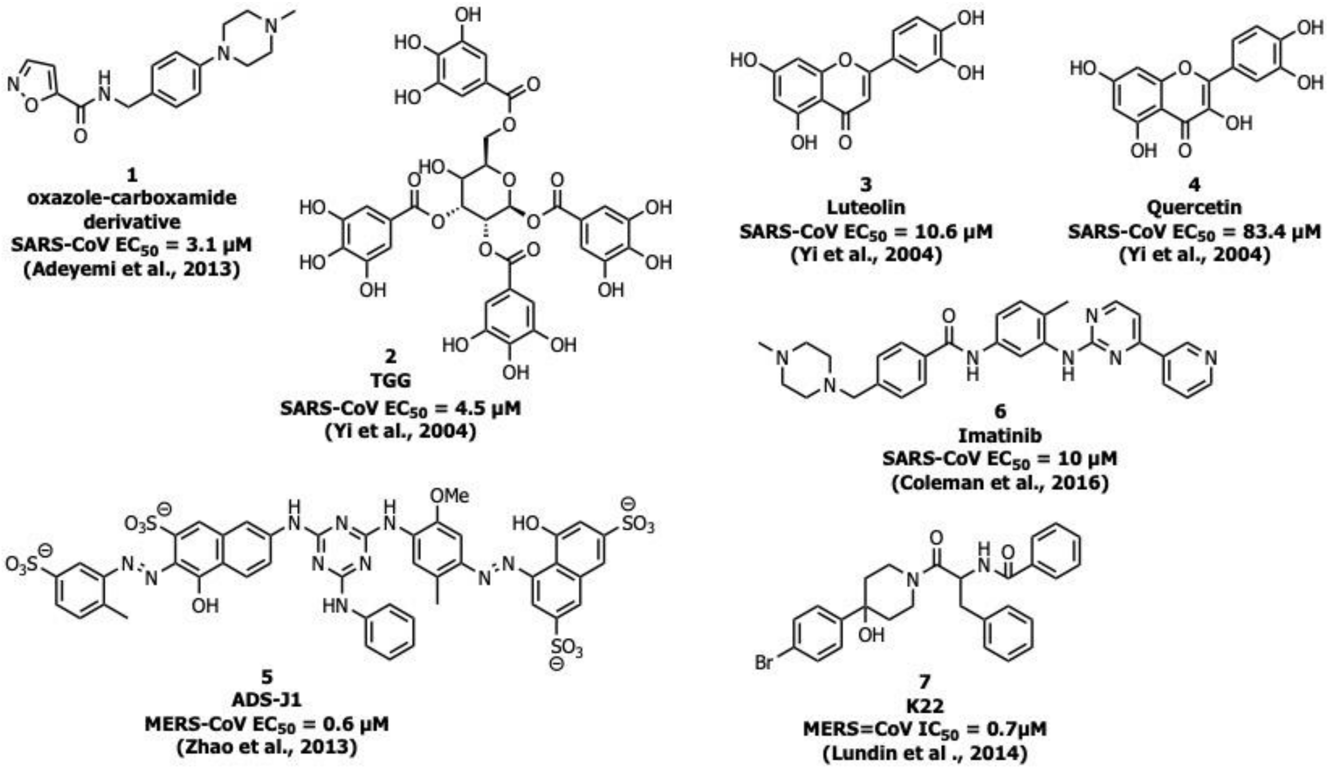

Figure 5. Inhibitors targeting spike protein S-domain, S1, and S2.

b) ACE2 Inhibitors

N-(2-aminoethyl)-1-aziridine-ethanamine (NAAE, **8**) was identified as a potent ACE2 inhibitor with an IC_{50} value of 57 μM and K_i value of 459 μM from a virtual screening of 140,000 compounds which inhibits SARS-CoV-1 by modulating S-glycoprotein-mediated membrane fusion (Huentelman et al., 2004). Savarino et al. reported the antiviral property of chloroquine (**9**), one of the safe and cost-effective drugs for the management of malaria and amoebiasis (Savarino et al., 2003). Chloroquine showed good *in-vitro* activity against mostly all lethal forms of coronavirus, SARS-CoV-1, MERS-CoV, and SARS-CoV-2. Against SARS-CoV-2, chloroquine showed an EC_{50} value of 5.47 μM (Yao et al., 2020; Keyaerts et

al., 2004; Devaux et al., 2020). It is assumed that chloroquine inhibits the production of proinflammatory cytokines (such as interleukin-6) by reducing acute respiratory distress syndrome (ARDS) (Savarino et al., 2003). The mechanistic study showed that chloroquine interferes with the terminal glycosylation of ACE2 and affects the interaction between the RBD of SARS-CoV-1 and ACE2 (Vincent et al., 2005).

A derivative of chloroquine, hydroxychloroquine (**10**) is another antimalarial drug experimented with against SARS-CoV-2, but still, the benefits are unclear (Mahase et al., 2020). It inhibits SARS-CoV-2 *in vitro* with an EC_{50} value of 0.74 μM (Yao et al., 2020). In March 2020, WHO announced that chloroquine and hydroxychloroquine were involved in the clinical trials for the treatment against SARS-CoV-2 (https://www.who.int). The trials were initiated by the US National Institutes of Health (NIH) in April 2020 and the study involved 96,032 subjects affected by SARS CoV-2, however, it is not clear regarding the effective benefits of hydroxychloroquine or chloroquine alone or in combination with macrolides against SARS-CoV-2 (like azithromycin or clarithromycin) (Mehra et al., 2020). Due to safety precautions, in May 2020, WHO announced that the clinical trials were stopped on using hydroxychloroquine as a drug for the treatment against SARS-CoV-2 (https://www.who.int).

One of the most potent and selective small-molecule inhibitors so far against ACE2 is MLN-4760 (**11**) with an IC_{50} of around 440 pM. It interacts with the zinc active site and imitates the transition state peptide. Hence MLN-4760 can be a useful inhibitor in the prevention of viral binding to ACE2 and results in the blockage of infection. (Towler et al., 2004).

Umifenovir or Arbidol (**12**) is a broad-spectrum inhibitor used as an antiviral drug against influenza. Arbidol inhibits the virus-host cell fusion and prevents the entry of virus which is also applicable for coronavirus (Kadam et al., 2017) and currently the drug is under clinical trials for the treatment of SARS-CoV-2 (Li et al., 2020). In another study, Arbidol (**12**) was

found to decrease the viral load and acts by binding with the S protein, and involves in trimerization that inhibits the host cell and membrane fusion (IC_{50} = 4.11 μM) (Wang et. al., 2020).

Hsiang et al. reported the active component from *Polygonum multiflorum* and *Rheum officinale*, emodin (**13**), blocks S protein interaction with ACE2 with an IC_{50} value of 10 μM and an EC_{50} value of 200 μM (Ho et al., 2007).

Recently, three selected compounds among 50,240 structurally diverse molecules, MP576, HE602, and VE607 (**14**) were evaluated against SARS-CoV M^{pro}, Helicase, and viral entry, respectively using a phenotype-based screening. Among them, VE607 (**14**), ((1-[3-(2-Hydroxyl-3-piperidin-1-yl-propoxy)-phenoxy]-3-piperidin-1-ylpropan-2-ol)), blocks SARS-CoV S protein pseudotype virus infection of 293T cells expressing ACE2 with an EC_{50} of 3 μM and inhibited SARS-CoV plaque formation with an EC_{50} of 1.6 μM (Kao et al., 2004).

Hanson et al., performed drug repurposing of 3384 small molecule drugs with 25 hits using a proximity-based assay that measures the binding of SARS-CoV-2 RBD and ACE2. (Hanson et al., 2020). Even though the unbound states of S protein and ACE2 lacks druggable pockets, there are well-defined pockets in the bound states for drug development. By application of computational approaches, Patil et al. showed that several antiviral drugs used against HCV and HIV viruses e.g., Atazanavir, Grazoprevir, Saquinavir, Simeprevir, Telaprevir, and Tipranavir, could serve as immediate investigational molecules and possibly as a potential candidate inhibitor (Patil et al., 2020).

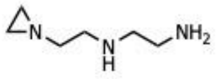

8
NAAE
SARS-CoV IC_{50} value = 57 μM
(Huentelman et al., 2004)

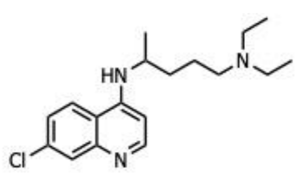

9
Chloroquine
SARS-CoV-2 EC_{50} = 5.47 μM
(Yao el al., 2020)

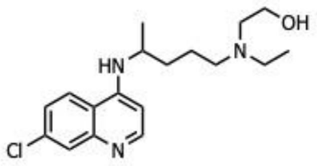

10
Hydroxychloroquine
SARS-CoV-2 EC_{50} = 0.74 μM
(Yao el al., 2020)

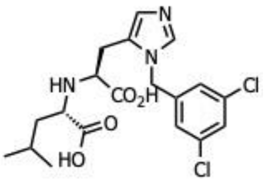

11
MLN-4760
IC_{50} = 0.00044 μM
(Towler et al., 2004)

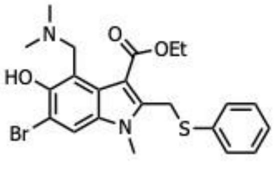

12
Umifenovir or Arbidol
SARS-CoV-2 EC_{50} = 4.11 μM
(Li et al., 2020)

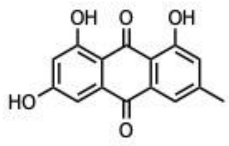

13
Emodin
SARS-CoV IC_{50} = 10 μM
EC_{50} = 200 μM
(Ho et al., 2007)

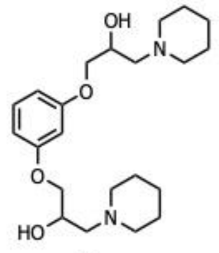

14
VE607
SARS-CoV EC_{50} = 3μM
(Kao et al., 2004)

Figure 6. Inhibitors for SARS-CoV-1 and -2 targeting ACE2.

c) Proteolytic inhibitors

Chlorpromazine (**15**), promethazine (**16**), and fluphenazine (**17**), neurotransmitter blockers, inhibits S protein-induced fusion of MERS-CoV and SARS-CoV-1 (Liu et al., 2015). Chlorpromazine, an inhibitor of clathrin-mediated endocytosis, was already reported to inhibit human CoV-229E, hepatitis C virus, infectious bronchitis virus, as well as mouse hepatitis virus-2 (MHV2) (Chu et al., 2004; Joki-Korpela et al., 2001; Krizanova et al., 1982; Nawa et al., 2003).

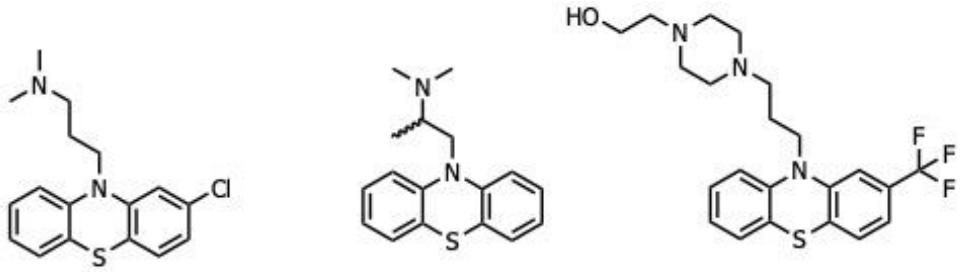

	15 Chlorpromazine	16 Promethazine	17 Fluphenazine
MERS-CoV: IC_{50}	9.51 μM	5.86 μM	11.80 μM
SARS-CoV: IC_{50} (Liu et al., 2015)	12.97 μM.	21.43 μM	11.80 μM

Figure 7. Neurotransmitter inhibitors targeting clathrin/non-clathrin pathways.

Ouabain and bufalin inhibitor blocks clathrin-mediated endocytosis and prevents MERS-CoV entry. The ouabain (50 nM) and bufalin (10 to 15 nM) inhibited infections by MERS-CoV and VSV (vesicular stomatitis virus). Kawase et al. identified camostat (**18**), a protease inhibitor, as a TMPRSS2 blocker at 10 μM in SARS-CoV-1. However, at a higher concentration (100 μM) the inhibition efficiency was only up to 65% which shows that 35% of entry happens via the endosomal cathepsin pathway. The study also showed >95 % blockade of viral entry with a combination treatment of EST (a cathepsin inhibitor) and **18** (Kawase et al., 2012). Complete inhibition of viral entry was also observed with a combination of both **18** and E-64d (a cathepsin inhibitor) (Hoffmann et al., 2020). Tissue cultures of another cysteine protease inhibitor, K11777 (**19**), showed inhibition in the ~~subnanomolar~~sub-nanomolar range against the replication of SARS-CoV-1 and MERS-CoV (Zhou et al., 2016). However further studies using tissue culture and animal models need to be carried out to confirm TMPRSS2 inhibition.

Teicoplanin blocks the entry of SARS-CoV-2 pseudoviruses (IC_{50} = 1.66 μM). Teicoplanin is a glycopeptide antibiotic used in the prophylactic treatment of Gram-positive bacterial infections including methicillin-resistant *Staphylococcus aureus* and *Enterococcus faecalis*. It

is also an inhibitor of cathepsin L of SARS-CoV-1, MERS-CoV, and Ebola virus, and preventing viral entry (Zhang et al., 2020).

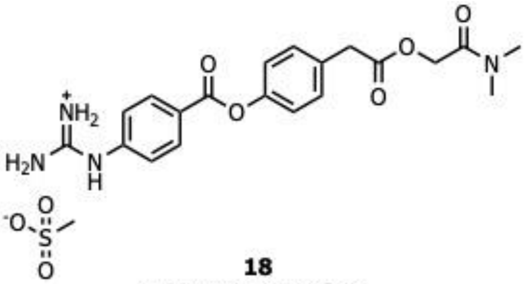

18
Camostat mesylate
SARS-CoV-2 EC_{50} = 1 µM
(Breining et al., 2020)

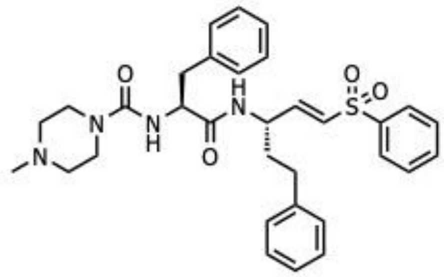

19
K11777
SARS-CoV IC_{50} =0.32nM
(Zhou et al., 2015)

Figure 8. Inhibitors targeting TMPRSS2.

Human cathepsin L, a cysteine endopeptidase, activates the S protein into a fusogenic state to escape the late endosomes, thereby interferes with the viral entry (Dana et al., 2020). MDL28170 (**20**) inhibits cathepsin-L-mediated substrate cleavage with IC_{50} and EC_{50} values of 2.5 nM and 100 nM, respectively (Simmons et al., 2005). CID 16725315 (**21**) and CID 23631927 (**22**) are SARS-CoV cathepsin L inhibitors reported with an IC_{50} value of 6.9 nM and 56 nM respectively (Shah et al., 2010). SSAA09E1 (**22**) was identified as an inhibitor of cathepsin L proteinase among ~14,000 compounds with an IC_{50} value of 5.33 µM. The compound **23** showed an EC_{50} value of around 6.4 µM in a pseudotype-based assay in 293T cells and was non-cytotoxic below 100 µM (Adedeji et al., 2013).

Adedeji et al. reported SSAA09E3 (**24**), as an inhibitor of virus-cell membrane fusion in pseudotype-based and antiviral-based assays. The viral entry inhibitor compound **24** showed an EC_{50} value of 9.7 µM, and a CC_{50} value of 20 µM against Pseudotype-based assay in 293T cells (Adedeji et al., 2013). E-64-D (**25**) blocked Cathepsin of both MERS-CoV and SARS-CoV-1 infections (Lu et al., 2014; Dyall et al., 2014).

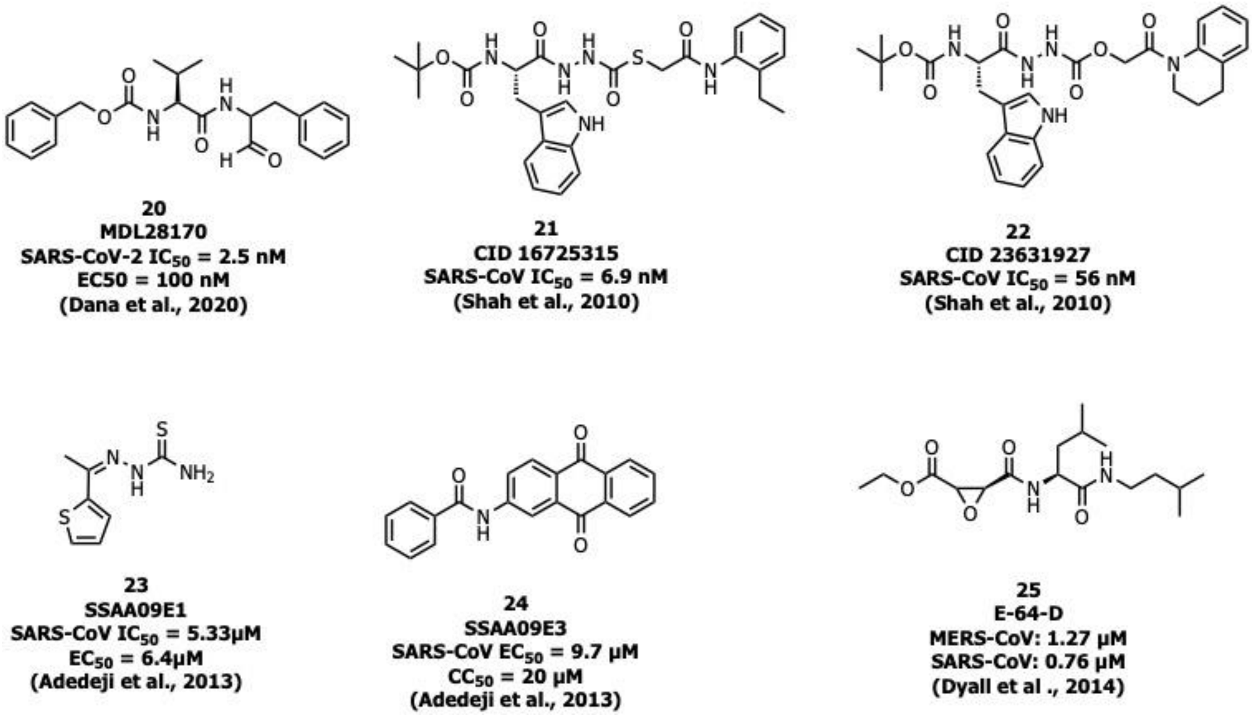

Figure 9. Cathepsin L inhibitors with antiviral activity.

d) Glucose Regulation Protein 78 Inhibitors

Understanding the M, E, and NSP6 proteins suggest that SARS-CoV S protein activates several unfolded protein response (UPR) effectors such as glucose-regulated protein (GRP) 78 (GRP78),, GRP94, and CCAAT/enhancer-binding protein (C/EBP) homologous protein during the transcription process. Endoplasmic reticulum (ER) stress and UPR are induced in infected cells during CoV infection. GRP94 and GRP78 or Binding immunoglobulin protein (BiP) are molecular chaperones and sensitive markers of ER stress (Chang et al., 2006).

The main UPR responsible for the viral entry including human and bat coronaviruses is GRP78. GRP78 is a luminal protein abundantly present in the ER and translocates to the cell surface during ER stress or coronavirus infection. After translocation to the cell surface membrane, GRP78 recognizes the virus by the substrate-binding domain (SBD) and mediates the entry of the virus into the cell. Further, it also plays a major role in the synthesis of viral protein, maturation and inactivates three enzymes responsible for cell death or differentiation

viz., Activating Transcription Factor (ATF) 6~~ (ATF6),~~, Protein kinase RNA-like Endoplasmic Reticulum Kinase (PERK), and Inositol-requiring Enzyme (IRE) 1~~ (IRE1).~~. Once the threshold of UPR accumulation reaches, these enzymes are released by the GRP78 and inhibit protein synthesis, and enhance the refolding (Chang et al., 2006; Ha et al., 2020; Ibrahim et al., 2020).

GRP78 is a crucial element for a viral infection to new cells. Depletion of GRP78 leads to a decrease in protein synthesis or improper folding of viral proteins and ~~the results found~~resulting in impaired budding or immature virions with diminished infectivity. GRP78 maintains the ER homeostasis and thereby expedite the viral component assembly by providing an ecosystem for growth. It is also captured into the viral particle and augment infection (Ha et al., 2020). It would be highly advantageous to inhibit the interaction between the S protein of SARS-CoV-2 and host cell receptor GRP78 to diminish the viral infection rate. (Ibrahim et al., 2020).

Rayner et al. determined that AR12 (a derivative of Celecoxib; **26**) inhibits the production of S protein by SARS-CoV-2 and thereby suppresses infectious virion generation. Compound **26** decreases ACE2 and GRP78 expression in cell surface and total GRP78 levels. Compound **26** not only catalytically inhibits the GRP78 ATPase activity but also reduces the chaperone proteins, which are linked with low S protein and the production of infectious virions (Rayner et al., 2020).

Allam et al., performed *in-silico* screening of a library of compounds and identified four potential phytochemicals (polyphenols *viz.*, epigallocatechin gallate (EGCG; **27**), homoeriodictyol (**28**), isorhamnetin (**29**), and curcumin (**30**)) and five peptides (satpdb18674 (**31**), satpdb18446 (**32**), satpdb12488 (**33**), satpdb14438 (**34**), and satpdb28899 (**35**)) inhibiting the interaction of SARS-CoV-2 S protein with GRP78 using molecular docking approaches (Allam et al., 2020),

Quimque et al. docked 97 antiviral molecules from fungi secondary metabolites followed by molecular dynamics simulation and in silico ADMET prediction. Three fumiquinazoline alkaloids scedapin C (**36**), quinadoline B (**37**), and nor-quinadoline A (**38**); the polyketide isochaetochromin D1 (**39**) and the terpenoid 11a-dehydroxyisoterreulactone A (**40**) exhibited strong in-silico inhibition against GRP78 of SAR-CoV-2 (Quimque et al., 2020). (**Figure 10)**

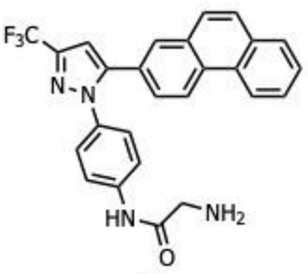

26
AR12
A Derivative of Celecoxib

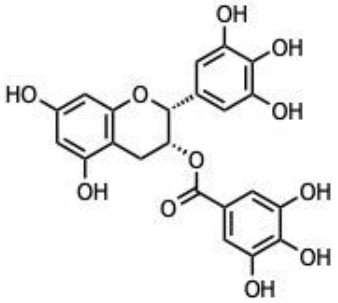

27
(-)-Epigallocatechin gallate

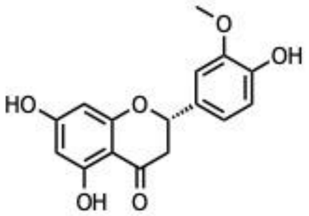

28
Homoeriodictyol

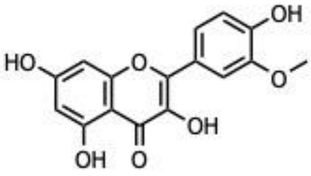

29
Isorhamnetin

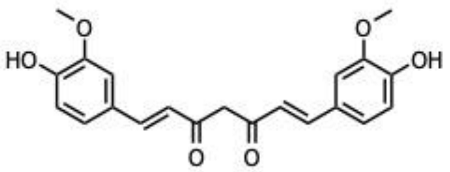

30
Curcumin

PEPTIDES: AMINO ACID SEQUENCE

qYGSFCTqLNRALSGIAAEq

31
satpdb18674

VLYNSTFFSTFKCYGVSATK

32
satpdb18446

PTTFMLKYdENGTITdAVdC

33
satpdb12488

SNNTIAIPTNFSISITTEVM

34
satpdb14438

RdVSdFTdSVRdPKTSEILd

35
satpdb28899

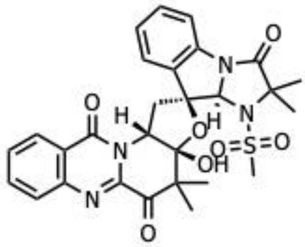

36
Scedapin C

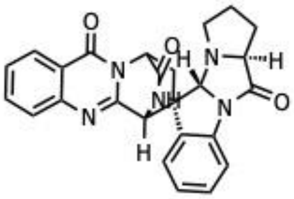

37
Quinadoline B

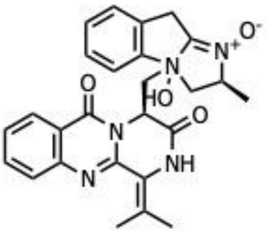

38
Norquinadoline A

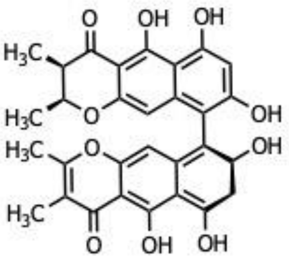

39
Isochaetochromin D1

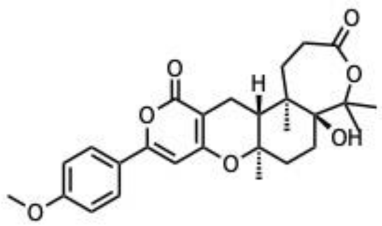

40
11a-dehydroxyisoterreulactone A

Figure 10. GRP78 inhibitors

5.3.2. Peptide-based inhibitors

The peptide-based or peptidomimetic inhibitors are larger molecules consisting of amino acid linkages with molecular size ranging from 1137 to 1814 Da (Ou et. al., 2020). Peptide-based inhibitors are hypothesized to prevent the entry of the virus into the human cells by disrupting the interaction of RBD of SARS-CoV-2 and ACE2. In a molecular dynamics simulation study carried out by Zhang et.al (2020), the protein-protein interactions were analyzed between SARS-COV-2 S protein and human ACE2. The S protein binding peptide 1 (SBP1; **41**) was synthesized with a sequence of 23 amino acids derived from ACE2 α1 helix and a dissociation constant K_d of 14.7nM suggests that SBP1 binds with the RBD of the S protein with low nanomolar affinity. It is the first in class peptide inhibitor preventing entry of the virus into human cells (Coutard et al., 2020).

Ho et al. reported peptide molecules significantly blocking the interaction of S protein with ACE2 (IC_{50} = 1.88 nM) (Ho et al., 2006). Han et al. stated that charged residues located at positions 22 and 57 are critical for entry of virus (Han et al., 2006). Based on this concept, various peptides were synthesized and found two compounds **42** (IC_{50} = 50 µM) and **43** (IC_{50} = 6.0 µM) with significant inhibitory activity against SARS-CoV-1. The introduction of glycine binding linker in compound **42** with ACE2 derived peptide (residues 351-357) further improvised the activity (IC_{50} = 100 nM) and reduced the cytotoxicity up to 200 µM (Han et al., 2006).

An *in-silico* design of antiviral (Seidah et. al., 2012; Chan et. al., 2020) HR2 derived peptide-like structure showed competitive inhibition of the binding of HR2 domain to the HR1 domain (Li et. al., 2008) (Bosch et. al., 2004). It should also be noted here that HR1 derived peptide failed to inhibit the viral infection due to the antiparallel binding of HR1 with three HR2 domains (Li et. al., 2008). This evidence suggests that targeting HR2 with HR2 derived peptide might prove a promising strategy in drug design against SARS-CoV-2.

Xia et al. reported a potent fusion inhibitor, EK1C4 (**44**) lipopeptide targeting S-glycoprotein-mediated cell membrane fusion of SARS-CoV-2, pseudotyped SARS-CoV-2, and live SARS-CoV-2 infection with IC_{50} values of 1.3 nM, 15.8 nM, and 36.5 nM, respectively (Xia et. al., 2020). IPB02 (**45**), another lipopeptide fusion inhibitor targeting the HR1 region were developed (Zhao et. al., 2013), which restricts the cell fusion activity of SARS-CoV-2 S-glycoprotein (IC_{50} = 25 nM) and SARS-CoV-2 pseudovirus (IC_{50} = 80 nM). (**Table 5**).

Table 5. Peptide inhibitors targeting S protein

Peptide	Peptide Sequence	Coronavirus	Activity
SBP1 (**41**)	IEEQAKTFLDKFNHEAEDLFYQS	SARS-CoV-1	K_d =14.7 nM
**42**	EEQAKTFLDKFNHEAEDLFYQSS	SARS-CoV-1	IC_{50} = 50 µM
**43**	EEQAKTFLDKFNHEAEDLFYQSSLASWNYNTNITEE	SARS-CoV-1	IC_{50} = 6.0 µM
EK1C4 (**44**)	SLDQINVTFLDLEYEMKK.EEAIKKLEESYIDLKEL-GSGSG-PEG4-Chol	SARS-CoV-2	IC_{50} = 1.3 nM
IPB02 (**45**)	ISGINASVVNIQKEIDRLNEVAKNLNESLIDLQELK (Chol)	SARS-CoV-2	IC_{50} = 25 nM

5.3.3. Natural product inhibitors

Heparin, a natural anti-coagulant was explored as antiviral agents for SARS-~~CoVs~~CoV, herpes, flavivirus, influenza, and HIV. A recent study has explored that SARS-CoV-2 utilizes HSPG (Heparin Sulfated peptide Glycan) for entry into the host cell ~~(~~Zhang et al., 2020). In order to understand the binding mechanism, Mycroft-West and his co-workers explored and reported the tight binding between S1 RBD and heparin using molecular modeling studies (Mycroft-West et al., 2020). Furthermore, Liu and his coworkers identified a common octasaccharide composed of IdoA2S-GlcNS6S that inhibits the spike – heparin interaction with an IC_{50} value of 38 nM (Liu et al., 2020).

Many natural products possessing immunomodulatory properties and antiviral activity such as curcumin~~,~~ (**46**), Nimbin~~,~~ (**47**), Fisetin (**48**) withaferin A, andrographolide, flavonoids/non-flavonoids were screened against S protein of SARS-CoV-2 (Vimal K. Maurya et al., 2020,

Pandey et al., 2020). An Indian Official Siddha Formulation termed Kabasura Kudineer Chooranam and JACOM (patented formulation) possessing (Kiran et al., 2020) 37 active constituents such as Magnoflorine, (**49**), 5-Hydroxy-7,8-dimethoxyflavanone, (**50**), Vasicinone, Quercetin, Luteolin, etc., were also subjected to docking studies against S protein. Twenty-three different Saikosaponins (Sinha et al., 2020) and 48 active compounds from all cinnamon varieties including Pavetannin C1 (**51**), Kaempferol (**52**) (Prasanth et al 2020) were screened against S protein. Though many of these compounds had shown good binding efficacy with S protein, further lab biological experiments are neededrequired to prove their predicted potency and mechanism of action.

Griffthin (GRFT) is a carbohydrate-binding protein consisting of 121 amino acids (12.7 kDa), inhibits viral entry by binding with S protein (O'Keefe et. al., 2010). GRFT reduced the percentage of cells killed by SARS-CoV with EC_{50} = 48 nM. Urtica dioica agglutinin, a small plant monomeric lectin inhibits SARS-CoV S protein with an IC_{50} value of 0.53 µg/ml. (Kumaki et al., 2011). (**Figure 11**)

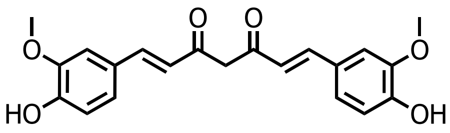

Curcumin

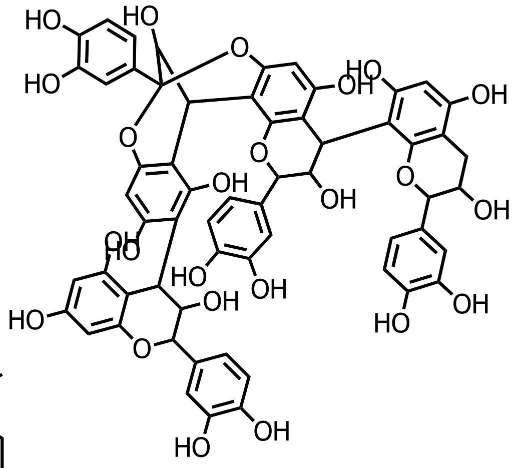

Pavetannin C1

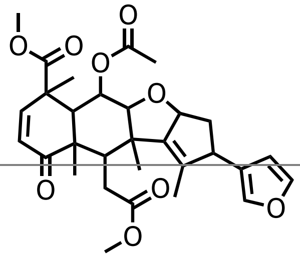

Nimbin

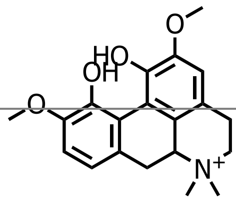

Magnoflorine

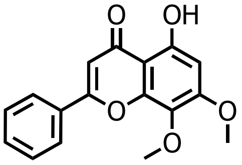

5-Hydroxy-7,8-dimethoxyflavanone

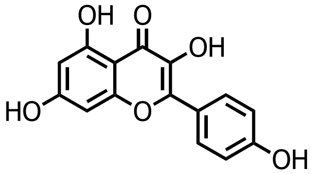

Kaempferol

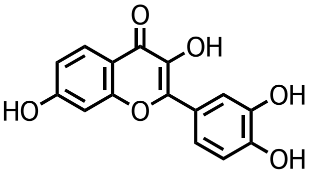

Fisetin

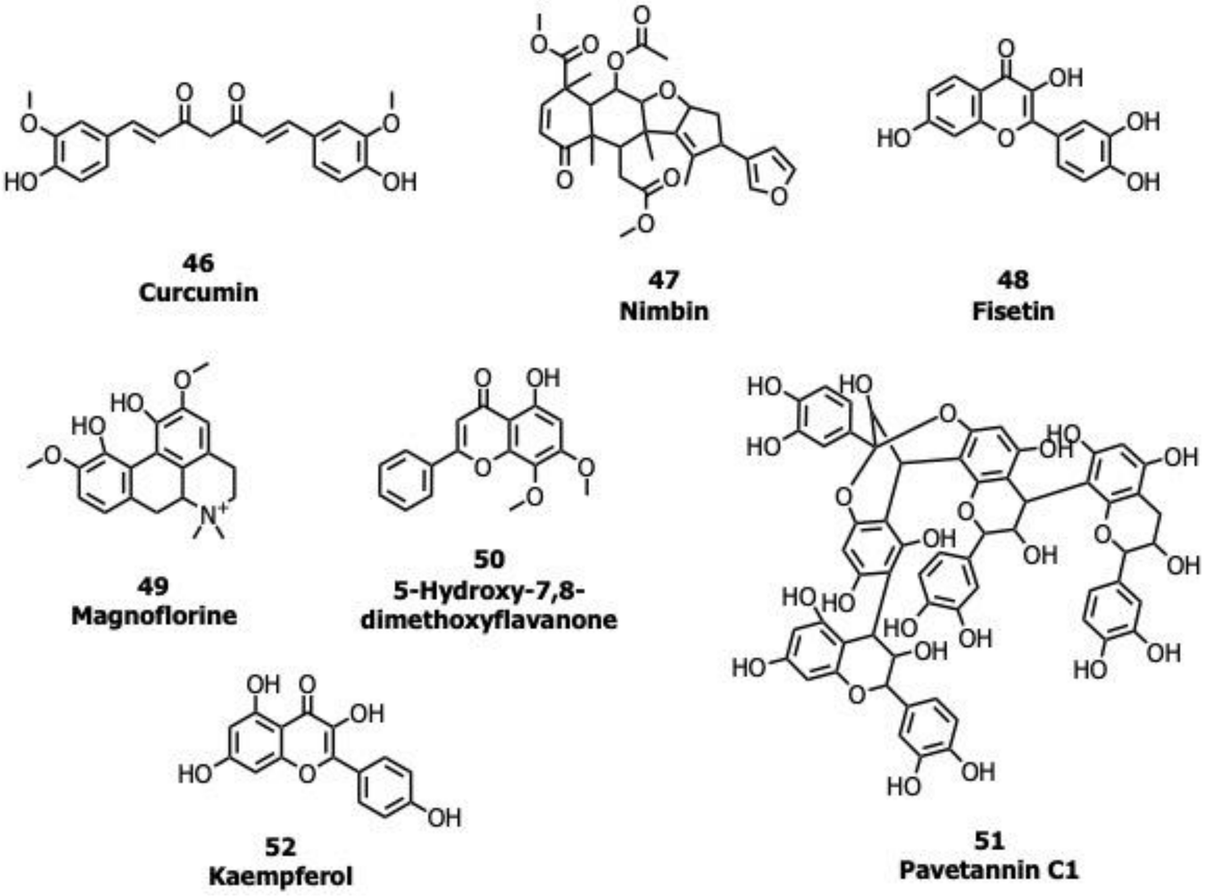

Figure 11. Natural products targeting S protein

6. Conclusion and future perspective

SARS-CoV-2 is one of the highly pathogenic and contagious human coronaviruses next to SARS-CoV-1 and MERS-CoV posing threat to human life globally. Despite advancements in modern medicine and the scientific community till date, it is not clear when the virus first originated and entered humans. This review in detail describesabout the spike protein focused on the structural information, binding mechanism of spike bindingalong with a special emphasis on the S1 and S2 domains comparedof SARS-CoV including the vaccines and inhibitors currently under development. SARS-CoV-2 and SARS-CoV-1 shares similar structural feature in Spike protein with SARSabout 74% similarity in RBD and MERS whenever required. Further development of a molecules differs in "RRAR furin recognition site". The difference in sequence, binding pattern and binding affinity of SARS-CoV-2 in

comparison to SAR-CoV-1 with the host cell receptor ACE2 makes the drug development process more tedious. The structural uniqueness in the Spike protein have led the focus of the drug discovery process towards the development of vaccines targeting full length S protein ~~including vaccine, ~~, RBD-sc dimers, mRNA, human monoclonal antibody and potential drug candidates including small molecule inhibitors~~ such as ~~, S-domain inhibitors, ACE2 inhibitors, proteolytic inhibitors, ~~Glucose Regulation Protein~~glucose regulation protein 78 ~~Inhibitors~~inhibitors, peptide-based inhibitors~~,~~ and natural product inhibitors~~ are also discussed.~~.

~~Prevention~~By the end of June 2020, mutations with the spike protein at the 614th amino acid position were identified due to an alteration in the single-nucleotide of the RNA code (D614G mutation) and ~~early diagnosis~~further mutations in the sequence found to be more transmissible (Korber et al., 2020, Yurkovetskiy et al., 2020). High number of sequence availability with their date of transmission in addition to high-resolution structural information provides an opportunity to analyze the evolutionary pathway and reveal the functional basis of the mutation at the molecular level. With the available data, integrating evolutionary and structural analysis with advanced computational techniques such as artificial intelligence provides important functional information of the mutations in SARS-CoV-s and to combat the current pandemic situation (Garvin et al., 2020).

Although the current pandemic situation has forced the scientists to develop vaccine in a very short period of time, there is a revolution in the vaccine development process. The researchers are ~~crucial~~successful with the development of vaccine based on mRNA encoding the spike protein with the successful examples from Moderna therapeutics and BioNtech. These vaccines are currently under Phase III clinical trials and approved in few countries on the basis of emergency condition. In addition to mRNA, an epitope can also be used to develop a vaccine as it can stimulate immune responses using isolated B-cell or T-cells.

These preliminary success stories give us an implication that targeting spike protein would be more advantageous in rapid drug discovery for controlling SARS-CoV-2. The spreading of Despite great efforts in the development of vaccines for HIV, HBV and HCV, the small molecule therapeutics had proven more effective for the treatment. In the current pandemic situation where the SARS-CoV-2 could have been controlled during an epidemic situation but worsened as a pandemic. Though drugs including has affected large number of population, an effective approach would be attacking the virus from every possible angle.

The application of repurposing strategies with the known antivirals had shownshow beneficial effects in certain populationsstudies, till date there is no systemic treatment for SARS-CoV-2. SmallTargeting small molecule inhibitors including natural inhibitors possibly inhibit the viral replication. Even after the identification of highly potent inhibitors the pharmacokinetic and toxicity studies must be cleared for the candidate molecules can't prevent the spread of SARS-CoV-2 hence an effective way to prevent and control with a long-term strategy is only by a vaccine. An epitope can also be used to develop a vaccine as it can stimulate immune responses using isolated B-cell or T-cells. Till date, two vaccines mRNA-1273 and BNT162b2 are in the process of in order to get the approval for human use. The frequent monitoring of the SARS-CoV-2 genome is essential to identify the various mutations that will help us in understatingas drug. The focus on the natural product drug discovery could possibly reduce the toxicity issues related to small molecule inhibitors. With the available information, it is very clear that future discoveries could aim in targeting the Spike protein, thereby identifying the capability of the phenotypic changes. Hence it is necessary to improve the surveillance using artificial intelligence to look for new viral strains periodically. Worldwide research papers on SARS-CoV-2 are increasing exponentially but we are no way near to cure SARS-CoV-2. The research should focus more on immunomodulatory therapies in the future for an effective way to prevent and control any

viral infection. and act on designing effective candidates in the prevention and transmission of the virus.

References

1. Adedeji AO, Severson W, Jonsson C, Singh K, Weiss SR, Sarafianos SG (2013). Novel inhibitors of severe acute respiratory syndrome coronavirus entry that act by three distinct mechanisms. *Journal of virology* 87**:** 8017-8028.

2. Allam L, Ghrifi F, Mohammed H, El Hafidi N, El Jaoudi R, El Harti J*, et al.* (2020). Targeting the GRP78-Dependant SARS-CoV-2 Cell Entry by Peptides and Small Molecules. *Bioinformatics and biology insights* 14**:** 1177932220965505-1177932220965505.

3. Alnefaie, A., and Albogami, S. (2020). Current approaches used in treating COVID-19 from a molecular mechanisms and immune response perspective. *Saudi pharmaceutical journal* 28: 1333-1352.

4. Bosch BJ, Martina BEE, van der Zee R, Lepault J, Haijema BJ, Versluis C*, et al.* (2004). Severe acute respiratory syndrome coronavirus (SARS-CoV) infection inhibition using spike protein heptad repeat-derived peptides. *Proceedings of the national academy of sciences of the United States of America* 101**:** 8455-8460.

5. Bahrami A & Ferns GA. (2020). Genetic and pathogenic characterization of SARS-CoV-2: *Future Virology* 15(8): 533–549.

~~5.~~**6.** Breining P., Frolund AL., Højen JF. *et al*. (2020). Camostat mesylate against SARS-CoV-2 and COVID-19-Rationale, dosing and safety. *Basic & Clinical Pharmacology Toxicology* 1-9. doi.org/10.1111/bcpt.13533

~~6.~~**7.** Chan CP, Siu KL, Chin KT, Yuen KY, Zheng B, Jin DY (2006). Modulation of the unfolded protein response by the severe acute respiratory syndrome coronavirus spike protein. *Journal of virology* 80**:** 9279-9287.

~~7.~~**8.** Chan, J. F. W., Kok, K. H., Zhu, Z., Chu, H., To, K. K. W., Yuan, S., et al. (2020). Genomic characterization of the 2019 novel human-pathogenic coronavirus isolated from a patient with atypical pneumonia after visiting Wuhan. *Emerging microbes & infections* 9, 221–236.

~~8.~~**9.** Chi X, Liu X, Wang C, Zhang X, Li X, Hou J*, et al.* (2020). Humanized single domain antibodies neutralize SARS-CoV-2 by targeting the spike receptor binding domain. *Nature communications* 11**:** 4528.

~~9.~~**10.** Chu JJ, Ng ML (2004). Infectious entry of West Nile virus occurs through a clathrin-mediated endocytic pathway. *Journal of virology* 78**:** 10543-10555.

~~10.~~**11.** Coleman CM, Sisk JM, Mingo RM, Nelson EA, White JM, Frieman MB (2016). Abelson Kinase Inhibitors Are Potent Inhibitors of Severe Acute Respiratory Syndrome Coronavirus and Middle East Respiratory Syndrome Coronavirus Fusion. *Journal of virology* 90**:** 8924-8933.

~~11.~~**12.** Coutard B, Valle C, de Lamballerie X, Canard B, Seidah NG, Decroly E (2020). The spike glycoprotein of the new coronavirus 2019-nCoV contains a furin-like cleavage site absent in CoV of the same clade. *Antiviral research* 176**:** 104742.

~~12.~~**13.** Courtney J. Mycroft-West, Dunhao Su, Yong Li, Scott E. Guimond, Timothy R. Rudd, Stefano Elli, Gavin Miller, Quentin M. Nunes, Patricia Procter, Antonella Bisio, Nicholas R. Forsyth, Jeremy E. Turnbull, Marco Guerrini, David G. Fernig, Edwin A.

Yates, Marcelo A. Lima, Mark A. Skidmore (2020). Heparin inhibits cellular invasion by SARS-CoV-2: structural dependence of the interaction of the surface protein (spike) S1 receptor binding domain with heparin. *bioRxiv : the preprint server for biology*. doi: https://doi.org/10.1101/2020.04.28.066761

13.14. Dai, L., Zheng, T., Xu, K., Han, Y., Xu, L., Huang, E., et al. (2020). A Universal Design of Betacoronavirus Vaccines against COVID-19, MERS, and SARS. *Cell* 182, 722-733.e11. doi:10.1016/j.cell.2020.06.035

14.15. Dana D, Pathak SK (2020). A Review of Small Molecule Inhibitors and Functional Probes of Human Cathepsin L. *Molecules* 25: 698.

15.16. Devaux CA, Rolain JM, Colson P, Raoult D (2020). New insights on the antiviral effects of chloroquine against coronavirus: what to expect for COVID-19? *International journal of antimicrobial agents* 55**:** 105938.

16.17. Dyall J, Coleman CM, Hart BJ, Venkataraman T, Holbrook MR, Kindrachuk J, *et al.* (2014). Repurposing of clinically developed drugs for treatment of Middle East respiratory syndrome coronavirus infection. *Antimicrobial agents and chemotherapy* 58**:** 4885-4893.

17.18. Garber K (2020). Coronavirus vaccine developers wary of errant antibodies. *Nature biotechnology*.

19. Garvin, M.R., T. Prates, E., Pavicic, M. *et al.* (2020). Potentially adaptive SARS-CoV-2 mutations discovered with novel spatiotemporal and explainable AI models. *Genome Biol* 21**,** 304. https://doi.org/10.1186/s13059-020-02191-0

18.20. Geller C, Varbanov M, Duval RE (2012). Human coronaviruses: insights into environmental resistance and its influence on the development of new antiseptic strategies. *Viruses* 4**:** 3044-3068.

19.21. Ghosh AK, Brindisi M, Shahabi D, Chapman ME, Mesecar AD (2020). Drug Development and Medicinal Chemistry Efforts toward SARS-Coronavirus and Covid-19 Therapeutics. *ChemMedChem* 15**:** 907-932.

20.22. Guangyu Zhao, Lanying Du, Cuiqing Ma, Ye Li, Lin Li, Vincent KM Poon, Lili Wang, Fei Yu, Bo-Jian Zheng, Shibo Jiang & Yusen Zhou (2013). A safe and convenient pseudovirus-based inhibition assay to detect neutralizing antibodies and screen for viral entry inhibitors against the novel human coronavirus MERS-CoV. *Journal of virology* 266.

21.23. Guo, Y. R., Cao, Q. D., Hong, Z. S., Tan, Y. Y., Chen, S. D., Jin, H. J., et al. (2020). The origin, transmission and clinical therapies on coronavirus disease 2019 (COVID-19) outbreak- A n update on the status. *Military medical research* 7:11. doi:10.1186/s40779-020-00240-0.

22.24. Ha DP, Van Krieken R, Carlos AJ, Lee AS (2020). The stress-inducible molecular chaperone GRP78 as potential therapeutic target for coronavirus infection. *The journal of infection* 81**:** 452-482.

23.25. Han DP, Penn-Nicholson A, Cho MW (2006). Identification of critical determinants on ACE2 for SARS-CoV entry and development of a potent entry inhibitor. *Virology* 350**:** 15-25.

24.26. Hanson QM, Wilson KM, Shen M, Itkin Z, Eastman RT, Shinn P, *et al.* (2020). Targeting ACE2–RBD Interaction as a Platform for COVID-19 Therapeutics: Development

and Drug-Repurposing Screen of an AlphaLISA Proximity Assay. *ACS pharmacology & translational science* 3**:** 1352-1360.

~~25.~~27. Ho TY, Wu SL, Chen JC, Li CC, Hsiang CY (2007). Emodin blocks the SARS coronavirus spike protein and angiotensin-converting enzyme 2 interaction. *Antiviral research* 74**:** 92-101.

~~26.~~28. Ho TY, Wu SL, Chen JC, Wei YC, Cheng SE, Chang YH, *et al.* (2006). Design and biological activities of novel inhibitory peptides for SARS-CoV spike protein and angiotensin-converting enzyme 2 interaction. *Antiviral research* 69**:** 70-76.

~~27.~~29. Hoffmann M, Hofmann-Winkler H, Smith JC, Krüger N, Sørensen LK, Søgaard OS, *et al.* (2020). Camostat mesylate inhibits SARS-CoV-2 activation by TMPRSS2-related proteases and its metabolite GBPA exerts antiviral activity. *bioRxiv : the preprint server for biology***:** 2020.2008.2005.237651.

~~28.~~30. Hoffmann, M., Kleine-Weber, H., and Pöhlmann, S. (2020). A Multibasic Cleavage Site in the Spike Protein of SARS-CoV-2 Is Essential for Infection of Human Lung Cells. *Mol. Cell* 78, 779-784.e5. doi:10.1016/j.molcel.2020.04.022.

~~29.~~31. Huang C, Wang Y, Li X, Ren L, Zhao J, Hu Y, *et al.* (2020). Clinical features of patients infected with 2019 novel coronavirus in Wuhan, China. *Lancet (London, England)* 395**:** 497-506.

~~30.~~32. Huang Y, Yang C, Xu X-f, Xu W, Liu S-w (2020). Structural and functional properties of SARS-CoV-2 spike protein: potential antivirus drug development for COVID-19. *Acta Pharmacologica Sinica* 41**:** 1141-1149.

~~31.~~33. Huentelman MJ, Zubcevic J, Hernández Prada JA, Xiao X, Dimitrov DS, Raizada MK, *et al.* (2004). Structure-based discovery of a novel angiotensin-converting enzyme 2 inhibitor. *Hypertension (Dallas, Tex. : 1979)* 44**:** 903-906.

~~32.~~34. Ibrahim IM, Abdelmalek DH, Elshahat ME, Elfiky AA (2020). COVID-19 spike-host cell receptor GRP78 binding site prediction. *The Journal of infection* 80**:** 554-562.

~~33.~~35. Inoue Y, Tanaka N, Tanaka Y, Inoue S, Morita K, Zhuang M, *et al.* (2007). Clathrin-dependent entry of severe acute respiratory syndrome coronavirus into target cells expressing ACE2 with the cytoplasmic tail deleted. *Journal of virology* 81**:** 8722-8729.

~~34.~~36. Iwasaki A, Yang Y (2020). The potential danger of suboptimal antibody responses in COVID-19. *Nature reviews. Immunology* 20**:** 339-341.

~~35.~~37. Jiang, S., Bottazzi, M. E., Du, L., Lustigman, S., Tseng, C. T. K., Curti, E., et al. (2012). Roadmap to developing a recombinant coronavirus S protein receptor-binding domain vaccine for severe acute respiratory syndrome. *Expert Rev. Vaccines*. doi:10.1586/erv.12.126.

~~36.~~38. Jiang S, He Y, Liu S (2005). SARS vaccine development. *Emerging infectious diseases* 11**:** 1016-1020.

39. Jiang S, Hillyer C, Du L (2020). Neutralizing antibodies against SARS-CoV-2 and other human coronaviruses. *Trends Immunol.* 41:355–359. doi: 10.1016/j.it.2020.03.007

~~37.~~40. Joki-Korpela P, Marjomäki V, Krogerus C, Heino J, Hyypiä T (2001). Entry of human parechovirus 1. *Journal of virology* 75**:** 1958-1967.

~~38.~~41. Kadam RU, Wilson IA (2017). Structural basis of influenza virus fusion inhibition by the antiviral drug Arbidol. *Proceedings of the National Academy of Sciences of the United States of America* 114**:** 206-214.

~~39.~~42. Kao RY, Tsui WH, Lee TS, Tanner JA, Watt RM, Huang JD, *et al.* (2004). Identification of novel small-molecule inhibitors of severe acute respiratory syndrome-associated coronavirus by chemical genetics. *Chemistry & biology* 11**:** 1293-1299.

~~40.~~43. Kawase M, Shirato K, van der Hoek L, Taguchi F, Matsuyama S (2012). Simultaneous treatment of human bronchial epithelial cells with serine and cysteine protease inhibitors prevents severe acute respiratory syndrome coronavirus entry. *Journal of virology* 86**:** 6537-6545.

~~41.~~44. Keyaerts E, Vijgen L, Maes P, Neyts J, Van Ranst M (2004). In vitro inhibition of severe acute respiratory syndrome coronavirus by chloroquine. *Biochemical and biophysical research communications* 323**:** 264-268.

~~42.~~45. Kiran G, Karthik L, Shree Devi MS, Sathiyarajeswaran P, Kanakavalli K, Kumar KM, *et al.* (2020). In Silico computational screening of Kabasura Kudineer - Official Siddha Formulation and JACOM against SARS-CoV-2 spike protein. *J Ayurveda Integr Med***:** S0975-9476(0920)30024-30023.

46. Korber B, Fischer WM, Gnanakaran S, Yoon H, Theiler J, Abfalterer W, *et al.* (2020) Tracking Changes in SARS-CoV-2 Spike: Evidence that D614G Increases Infectivity of the COVID-19 Virus. *Cell* 182(4):812-827.e19

~~43.~~47. Krammer F (2020). SARS-CoV-2 vaccines in development. *Nature* 586**:** 516-527.

~~44.~~48. Krishnamoorthy, S., Swain, B., Verma, R.S. et al. (2020). SARS-CoV, MERS-CoV, and 2019-nCoV viruses: an overview of origin, evolution, and genetic variations. VirusDis. 31, 411–423. doi.org/10.1007/s13337-020-00632-9.

~~45.~~49. Krizanová O, Ciampor F, Veber P (1982). Influence of chlorpromazine on the replication of influenza virus in chick embryo cells. *Acta virologica* 26**:** 209-216.

~~46.~~50. Kumaki Y, Wandersee MK, Smith AJ, Zhou Y, Simmons G, Nelson NM, *et al.* (2011). Inhibition of severe acute respiratory syndrome coronavirus replication in a lethal SARS-CoV BALB/c mouse model by stinging nettle lectin, Urtica dioica agglutinin. *Antiviral research* 90**:** 22-32.

~~47.~~51. Lan, J., Ge, J., Yu, J., Shan, S., Zhou, H., Fan, S., et al. (2020). Structure of the SARS-CoV-2 spike receptor-binding domain bound to the ACE2 receptor. *Nature* 581, 215–220. doi:10.1038/s41586-020-2180-5.

~~48.~~52. Li G, De Clercq E (2020). Therapeutic options for the 2019 novel coronavirus (2019-nCoV). *Nature reviews. Drug discovery* 19**:** 149-150.

~~49.~~53. Li W, Li L, Sun T, He Y, Liu G, Xiao Z, *et al.* (2020). Spike protein-based epitopes predicted against SARS-CoV-2 through literature mining. *Medicine in novel technology and devices* 8**:** 100048.

~~50.~~54. Liu L, Chopra P, Li X, Wolfert MA, Tompkins SM, Boons G-J (2020). SARS-CoV-2 spike protein binds heparan sulfate in a length- and sequence-dependent manner. *bioRxiv : the preprint server for biology*: 2020.2005.2010.087288.

~~51.~~55. Liu, S., Xiao, G., Chen, Y., He, Y., Niu, J., Escalante, C. R., et al. (2004). Interaction between heptad repeat 1 and 2 regions in spike protein of SARS-associated coronavirus: Implications for virus fusogenic mechanism and identification of fusion inhibitors. *Lancet* 363, 938–947. doi:10.1016/S0140-6736(04)15788-7.

~~52.~~56. Liu, L., Wei, Q., Lin, Q., Fang, J., Wang, H., Kwok, H., et al. (2019). Anti-spike IgG causes severe acute lung injury by skewing macrophage responses during acute SARS-CoV infection. *JCI insight* 4. doi:10.1172/jci.insight.123158.

~~53.~~57. Liu Q, Xia S, Sun Z, Wang Q, Du L, Lu L, *et al.* (2015). Testing of Middle East respiratory syndrome coronavirus replication inhibitors for the ability to block viral entry. *Antimicrobial agents and chemotherapy* 59**:** 742-744.

~~54.~~58. Lu L, Liu Q, Zhu Y, Chan KH, Qin L, Li Y, *et al.* (2014). Structure-based discovery of Middle East respiratory syndrome coronavirus fusion inhibitor. *Nature communications* 5**:** 3067.,

~~55.~~59. Lundin A, Dijkman R, Bergström T, Kann N, Adamiak B, Hannoun C, *et al.* (2014). Targeting membrane-bound viral RNA synthesis reveals potent inhibition of diverse coronaviruses including the middle East respiratory syndrome virus. *PLoS pathogens* 10**:** e1004166.

~~56.~~60. Mahase E (2020). Covid-19: six million doses of hydroxychloroquine donated to US despite lack of evidence. *BMJ (Clinical research ed.)* 368**:** m1166.

~~57.~~61. Maurya VK, Kumar S, Prasad AK, Bhatt MLB, Saxena SK (2020). Structure-based drug designing for potential antiviral activity of selected natural products from Ayurveda against SARS-CoV-2 spike glycoprotein and its cellular receptor. *Virusdisease* 31**:** 179-193.

~~58.~~62. McKee, D. L., Sternberg, A., Stange, U., Laufer, S., and Naujokat, C. (2020). Candidate drugs against SARS-CoV-2 and COVID-19. *Pharmacological research* 157. doi:10.1016/j.phrs.2020.104859.

~~59.~~63. Mehra MR, Desai SS, Ruschitzka F, Patel AN. Hydroxychloroquine or chloroquine with or without a macrolide for treatment of COVID-19: a multinational registry analysis. *Lancet*. May 22, 2020; DOI: https://doi.org/10.1016/S0140-6736(20)31180-6.

~~60.~~64. Modjarrad, K., Moorthy, V. S., Ben Embarek, P., Van Kerkhove, M., Kim, J., and Kieny, M. P. (2016). A roadmap for MERS-CoV research and product development: Report from a World Health Organization consultation. *Nature medicine* 22, 701–705. doi:10.1038/nm.4131.

~~61.~~65. Mulligan MJ, Lyke KE, Kitchin N, Absalon J, Gurtman A, Lockhart S, *et al.* (2020). Phase I/II study of COVID-19 RNA vaccine BNT162b1 in adults. *Nature* 586**:** 589-593.

~~62.~~66. O'Keefe BR, Giomarelli B, Barnard DL, Shenoy SR, Chan PKS, McMahon JB, *et al.* (2010). Broad-spectrum in vitro activity and in vivo efficacy of the antiviral protein griffithsin against emerging viruses of the family Coronaviridae. *Journal of virology* 84**:** 2511-2521.

~~63.~~67. Ou, X., Liu, Y., Lei, X. *et al.* Characterization of spike glycoprotein of SARS-CoV-2 on virus entry and its immune cross-reactivity with SARS-CoV. *Nature communications* **11,** 1620 (2020).

~~64.~~68. Nawa M, Takasaki T, Yamada KI, Kurane I, Akatsuka T (2003). Interference in Japanese encephalitis virus infection of Vero cells by a cationic amphiphilic drug, chlorpromazine. *The Journal of general virology* 84**:** 1737-1741.

~~65.~~69. Pandey P, Rane JS, Chatterjee A, Kumar A, Khan R, Prakash A, *et al.* (2020). Targeting SARS-CoV-2 spike protein of COVID-19 with naturally occurring phytochemicals: an in silico study for drug development. *Journal of biomolecular structure & dynamics***:** 1-11.

~~66.~~70. Pal M, Berhanu G, Desalegn C, Kandi V (2020). Severe Acute Respiratory Syndrome Coronavirus-2 (SARS-CoV-2): An Update. *Cureus* 12**:** e7423-e7423.

~~67.~~71. Patil, Sachin; Hofer, Jeremy; Ballester, Pedro J.; Fattakhova, Elena; DiFlumeri, Juliette; Campbell, Autumn; et al. (2020): Drug Repurposing for Covid-19: Discovery of Potential Small-Molecule Inhibitors of Spike Protein-ACE2 Receptor Interaction Through Virtual Screening and Consensus Scoring. *ChemRxiv. Preprint*. https://doi.org/10.26434/chemrxiv.12482435.v1

~~68.~~72. Pillaiyar T, Manickam M, Namasivayam V, Hayashi Y, Jung SH (2016). An Overview of Severe Acute Respiratory Syndrome-Coronavirus (SARS-CoV) 3CL Protease Inhibitors: Peptidomimetics and Small Molecule Chemotherapy. *Journal of medicinal chemistry* 59**:** 6595-6628.

~~69.~~73. Pillay TS (2020). Gene of the month: the 2019-nCoV/SARS-CoV-2 novel coronavirus spike protein. *Journal of clinical pathology* 73**:** 366-369.

~~70.~~74. Prasanth DSNBK, Murahari M, Chandramohan V, Panda SP, Atmakuri LR, Guntupalli C (2020). In silico identification of potential inhibitors from Cinnamon against main protease and spike glycoprotein of SARS CoV-2. *Journal of biomolecular structure & dynamics***:** 1-15.

~~71.~~75. Rayner JO, Roberts RA, Kim J, Poklepovic A, Roberts JL, Booth L, *et al.* (2020). AR12 (OSU-03012) suppresses GRP78 expression and inhibits SARS-CoV-2 replication. *Biochem Pharmacol* 182**:** 114227-114227.

~~72.~~76. Quimque MTJ, Notarte KIR, Fernandez RAT, Mendoza MAO, Liman RAD, Lim JAK, *et al.* (2020). Virtual screening-driven drug discovery of SARS-CoV2 enzyme inhibitors targeting viral attachment, replication, post-translational modification and host immunity evasion infection mechanisms. *Journal of biomolecular structure & dynamics***:** 1-18.

~~73.~~77. Robson, B. (2020). Computers and viral diseases. Preliminary bioinformatics studies on the design of a synthetic vaccine and a preventative peptidomimetic antagonist against the SARS-CoV-2 (2019-nCoV, COVID-19) coronavirus. *Comput. Biol. Med.* 119. doi:10.1016/j.compbiomed.2020.103670.

~~74.~~78. Savarino A, Boelaert JR, Cassone A, Majori G, Cauda R (2003). Effects of chloroquine on viral infections: an old drug against today's diseases? *The Lancet. Infectious diseases* 3**:** 722-727.

~~75.~~79. Seidah, N. G., and Prat, A. (2012). The biology and therapeutic targeting of the proprotein convertases. *Nat. Rev. Drug Discov.* 11, 367–383. doi:10.1038/nrd3699.

~~76.~~80. Shah PP, Wang T, Kaletsky RL, Myers MC, Purvis JE, Jing H, *et al.* (2010). A small-molecule oxocarbazate inhibitor of human cathepsin L blocks severe acute respiratory syndrome and ebola pseudotype virus infection into human embryonic kidney 293T cells. *Molecular pharmacology* 78**:** 319-324.

~~77.~~81. Shirato K, Kawase M, Matsuyama S (2013). Middle East respiratory syndrome coronavirus infection mediated by the transmembrane serine protease TMPRSS2. *Journal of virology* 87**:** 12552-12561.

~~78.~~82. Sinha SK, Shakya A, Prasad SK, Singh S, Gurav NS, Prasad RS, *et al.* (2020). An in-silico evaluation of different Saikosaponins for their potency against SARS-CoV-2 using NSP15 and fusion spike glycoprotein as targets. *Journal of biomolecular structure & dynamics***:** 1-12.

~~79.~~83. Simmons G, Gosalia DN, Rennekamp AJ, Reeves JD, Diamond SL, Bates P (2005). Inhibitors of cathepsin L prevent severe acute respiratory syndrome coronavirus entry. *Proceedings of the National Academy of Sciences of the United States of America* 102**:** 11876-11881.

~~80.~~84. Sisk JM, Frieman MB, Machamer CE (2018). Coronavirus S protein-induced fusion is blocked prior to hemifusion by Abl kinase inhibitors. *The Journal of general virology* 99**:** 619-630.

~~81.~~85. Song, W., Gui, M., Wang, X., and Xiang, Y. (2018). Cryo-EM structure of the SARS coronavirus spike glycoprotein in complex with its host cell receptor ACE2. *PLoS Pathog.* 14. doi:10.1371/journal.ppat.1007236.

~~82.~~86. Tetro JA (2020). Is COVID-19 receiving ADE from other coronaviruses? *Microbes and infection* 22**:** 72-73.

~~83.~~87. Tian, X., Li, C., Huang, A., Xia, S., Lu, S., Shi, Z., et al. (2020). Potent binding of 2019 novel coronavirus spike protein by a SARS coronavirus-specific human monoclonal antibody. *Emerg. Microbes Infect.* 9, 382–385. doi:10.1080/22221751.2020.1729069

~~84.~~88. Towler P, Staker B, Prasad SG, Menon S, Tang J, Parsons T, *et al.* (2004). ACE2 X-ray structures reveal a large hinge-bending motion important for inhibitor binding and catalysis. *The Journal of biological chemistry* 279**:** 17996-18007.

~~85.~~89. Ulrich H, Pillat MM, Tárnok A (2020). Dengue Fever, COVID-19 (SARS-CoV-2), and Antibody-Dependent Enhancement (ADE): A Perspective. *Cytometry. Part A : the journal of the International Society for Analytical Cytology* 97**:** 662-667.

~~86.~~90. Vincent MJ, Bergeron E, Benjannet S, Erickson BR, Rollin PE, Ksiazek TG, *et al.* (2005). Chloroquine is a potent inhibitor of SARS coronavirus infection and spread. *Virology journal* 2**:** 69.

~~87.~~91. Walls AC, Park Y-J, Tortorici MA, Wall A, McGuire AT, Veesler D (2020). Structure, Function, and Antigenicity of the SARS-CoV-2 Spike Glycoprotein. *Cell* 181**:** 281-292.e286.

~~88.~~92. Walls AC, Xiong X, Park Y-J, Tortorici MA, Snijder J, Quispe J, *et al.* (2019). Unexpected Receptor Functional Mimicry Elucidates Activation of Coronavirus Fusion. *Cell* 176**:** 1026-1039.e1015.

~~89.~~93. Wang W., Xu Y., Gao R., Lu R., Han K., Wu G., Tan W~~.(~~. (2020). Detection of SARS-CoV-2 in Different Types of Clinical Specimens. *JAMA - J. Am. Med. Assoc.* 323, 1843–1844. doi:10.1001/jama.2020.3786.

~~90.~~94. Wang, Q., Zhang, L., Kuwahara, K., Li, L., Liu, Z., Li, T., et al. (2016). Immunodominant SARS coronavirus epitopes in humans elicited both enhancing and neutralizing effects on infection in non-human primates. *ACS Infect. Dis.* 2, 361–376. doi:10.1021/acsinfecdis.6b00006.

~~91.~~95. Wang K, Chen W, Zhou Y, et al. SARS-CoV-2 invades host cells via a novel route: CD147-spike protein. *BioRxiv* 2020 (DOI: 10.1101/2020.03.14.988345)

~~92.~~96. Wang H, Yang P, Liu K, Guo F, Zhang Y, Zhang G, *et al.* (2008). SARS coronavirus entry into host cells through a novel clathrin- and caveolae-independent endocytic pathway. *Cell research* 18**:** 290-301.

~~93.~~97. Wang, W., Xu, Y., Gao, R., Lu, R., Han, K., Wu, G., et al. (2020). Detection of SARS-CoV-2 in Different Types of Clinical Specimens. *JAMA - J. Am. Med. Assoc.* 323, 1843–1844. doi:10.1001/jama.2020.3786.

~~94.~~98. Wang, C., Horby, P. W., Hayden, F. G., and Gao, G. F. (2020). A novel coronavirus outbreak of global health concern. *Lancet* 395, 470–473. doi:10.1016/S0140-6736(20)30185-9.

~~95.~~99. Wang, Q., Zhang, L., Kuwahara, K., Li, L., Liu, Z., Li, T., et al. (2016). Immunodominant SARS coronavirus epitopes in humans elicited both enhancing and neutralizing effects on infection in non-human primates. *ACS infectious diseases* 2, 361–376. doi:10.1021/acsinfecdis.6b00006.

~~96.~~100. Wrapp D, Wang N, Corbett KS, Goldsmith JA, Hsieh CL, Abiona O, *et al.* (2020). Cryo-EM structure of the 2019-nCoV spike in the prefusion conformation. *Science* 367**:** 1260-1263.

~~97.~~101. Wu F, Zhao S, Yu B, Chen YM, Wang W, Song ZG, *et al.* (2020). A new coronavirus associated with human respiratory disease in China. 579**:** 265-269.

~~98.~~102. Xia S, Liu M, Wang C, Xu W, Lan Q, Feng S, *et al.* (2020). Inhibition of SARS-CoV-2 (previously 2019-nCoV) infection by a highly potent pan-coronavirus fusion inhibitor targeting its spike protein that harbors a high capacity to mediate membrane fusion. *Cell research* 30**:** 343-355.

~~99.~~103. Yan R, Zhang Y, Li Y, Xia L, Guo Y, Zhou Q (2020). Structural basis for the recognition of SARS-CoV-2 by full-length human ACE2. 367**:** 1444-1448.

~~100.~~104. Yang J, Wang W, Chen Z, Lu S, Yang F, Bi Z, *et al.* (2020). A vaccine targeting the RBD of the S protein of SARS-CoV-2 induces protective immunity. *Nature* 586**:** 572-577.

~~101.~~105. Yang Y, Du L, Liu C, Wang L, Ma C, Tang J, *et al.* (2014). Receptor usage and cell entry of bat coronavirus HKU4 provide insight into bat-to-human transmission of

MERS coronavirus. *Proceedings of the National Academy of Sciences of the United States of America* 111**:** 12516-12521.

102.106. Yao X, Ye F, Zhang M, Cui C, Huang B, Niu P, *et al.* (2020). In Vitro Antiviral Activity and Projection of Optimized Dosing Design of Hydroxychloroquine for the Treatment of Severe Acute Respiratory Syndrome Coronavirus 2 (SARS-CoV-2). *Clinical infectious diseases : an official publication of the Infectious Diseases Society of America* 71**:** 732-739.

103.107. Yi L, Li Z, Yuan K, Qu X, Chen J, Wang G, *et al.* (2004). Small molecules blocking the entry of severe acute respiratory syndrome coronavirus into host cells. *Journal of virology* 78**:** 11334-11339.

108. Yurkovetskiy L, Wang X, Pascal KE, Tomkins-Tinch C, Nyalile TP, Wang Y, *et al.* (2020). Structural and Functional Analysis of the D614G SARS-CoV-2 Spike Protein Variant. *Cell*. 183(3):739-751.e8

104.109. Zhai, P., Ding, Y., Wu, X., Long, J., Zhong, Y., and Li, Y. (2020). The epidemiology, diagnosis and treatment of COVID-19. *Int. J. Antimicrob. Agents* 55. doi:10.1016/j.ijantimicag.2020.105955.

105.110. Zhang JS, Ma C, Yu F, Liu J, Zou F, Pan T, Zhang H. Teicoplanin potently blocks the cell entry of 2019-nCoV. *BioRxiv* (DOI: 10.1101/2020.02.05.935387).

106.111. Zhou N, Pan T, Zhang J, Li Q, Zhang X, Bai C, *et al.* (2016). Glycopeptide Antibiotics Potently Inhibit Cathepsin L in the Late Endosome/Lysosome and Block the Entry of Ebola Virus, Middle East Respiratory Syndrome Coronavirus (MERS-CoV), and Severe Acute Respiratory Syndrome Coronavirus (SARS-CoV). *The Journal of biological chemistry* 291**:** 9218-9232.

107.112. Zhou P, Yang XL, Wang XG, Hu B, Zhang L, Zhang W, *et al.* (2020). A pneumonia outbreak associated with a new coronavirus of probable bat origin. *Nature* 579**:** 270-273.

108.113. Zhou Y, Vedantham P, Lu K, Agudelo J, Carrion R, Jr., Nunneley JW, *et al.* (2015). Protease inhibitors targeting coronavirus and filovirus entry. *Antiviral research* 116**:** 76-84.

109.114. Zhou, Y., Yang, Y., Huang, J., Jiang, S., and Du, L. (2019). Advances in MERS-CoV vaccines and therapeutics based on the receptor-binding domain. *Viruses* 11. doi:10.3390/v11010060.
