# Supplementary material for: An Overview of Spike Surface Glycoprotein in Severe Acute Respiratory Syndrome–Coronavirus
Source: Front Mol Biosci. 2021 Mar 16;8:637550. doi: 10.3389/fmolb.2021.637550 (PMC8058706; doi:10.3389/fmolb.2021.637550)
Supplement: Supplementary file 2 [file Table1.DOC]

**Supplementary Information**

**An Overview of Spike Surface Glycoprotein in Severe Acute Respiratory Syndrome–Coronavirus**

Muthu Kumaradoss Kathiravan^1,2#^, Srimathi Radhakrishnan^1,2#^, Vigneshwaran Namasivayam^3*^, Senthilkumar Palaniappan^4*^

^1^Department of Pharmaceutical Chemistry, SRM College of Pharmacy, SRMIST, Kattankulathur, Kancheepuram 603 203. Tamil Nadu. India.

^2^Dr. APJ Abdul Kalam Research Lab, SRM College of Pharmacy, SRMIST, Kattankulathur, Kancheepuram 603 203. Tamil Nadu. India.

^3^Pharmaceutical Institute, University of Bonn, 53121 Bonn, Germany. vnamasiv@uni-bonn.de

^4^Faculty of Pharmacy, Karpagam Academy of Higher Education, Pollachi Main Road, Eachanari Post, Coimbatore 641 021. Tamil Nadu. India. drsenthilkumar.p@kahedu.edu.in.

^#^These authors contributed equally to this work

^*^Corresponding authors

**Supplementary Table S1. Summary of the crystal structures available for MERS-CoV, SARS-CoV-1 and SARS-CoV-2.**

|  |  | **SARS-CoV-2** | **MERS-CoV** | **SARS-CoV-1** | **Total** |
| --- | --- | --- | --- | --- | --- |
| **Method** | **cryo-EM** | 129 | 15 | 17 | 161 |
|  | **X-ray** | 29 | 23 | 22 | 74 |
|  |  | 158 | 38 | 39 | **235** |
|  | | | | | |
| **Release**  **Year** | **before 2015** | - | 3 | 18 | 21 |
|  | **2015** | - | 2 | - | 2 |
|  | **2016** | - | - | - | 0 |
|  | **2017** | - | 20 | 3 | 23 |
|  | **2018** | - | 3 | 12 | 15 |
|  | **2019** | - | 8 | 3 | 11 |
|  | **2020** | 158 | 2 | 3 | 163 |
| **Total number of reported structures** | | | | | **235** |

**Supplementary Table S2. List of crystal structure available for MERS-CoV, SARS-CoV-1, and SARS-CoV-2 with their PDB ID sorted according to their resolution, domain which co-crystallized as complex with the month and year released.**

| **PDB ID** | **Experimental Method** | **Release Year** | **Resolution (Å)** | **Domain** | **Complex** |
| --- | --- | --- | --- | --- | --- |
| **MERS-CoV** | | | | | |
| 5X4R | X-RAY | 2017 | 1.5 |  |  |
| 5GSB | X-RAY | 2017 | 1.8 |  |  |
| 5GSV | X-RAY | 2017 | 2.0 |  |  |
| 5VYH | X-RAY | 2017 | 2.0 | NTD |  |
| 6C6Z | X-RAY | 2018 | 2.1 | RBD |  |
| 5GSR | X-RAY | 2017 | 2.2 |  |  |
| 4NJL | X-RAY | 2014 | 2.3 |  |  |
| 6PXH | X-RAY | 2019 | 2.3 | NTD | Fab |
| 5GR7 | X-RAY | 2017 | 2.4 |  |  |
| 5DO2 | X-RAY | 2015 | 2.4 |  |  |
| 5GSX | X-RAY | 2017 | 2.5 |  |  |
| 6J2J | X-RAY | 2019 | 2.5 |  |  |
| 5GMQ | X-RAY | 2017 | 2.7 | RBD |  |
| 3D0G | X-RAY | 2008 | 2.8 | RBD | ACE2 |
| 5YY5 | X-RAY | 2018 | 2.8 | RBD |  |
| 7C02 | X-RAY | 2020 | 2.9 | RBD |  |
| 6J11 | X-RAY | 2019 | 3.0 | NTD |  |
| 4L72 | X-RAY | 2013 | 3.0 | RBD | DPP4 |
| 6L8Q | X-RAY | 2019 | 3.1 |  |  |
| 4ZS6 | X-RAY | 2015 | 3.2 | RBD |  |
| 5ZVK | X-RAY | 2019 | 3.3 | HR1 Motif | Pan-CoV’s inhibitor EK1 |
| 6WAR | X-RAY | 2020 | 3.4 | RBD |  |
| 5ZXV | X-RAY | 2018 | 4.5 | RBD |  |
| 6NB3 | cryo-EM | 2019 | 3.5 |  |  |
| 5W9I | cryo-EM | 2017 | 3.6 |  |  |
| 6NB4 | cryo-EM | 2019 | 3.6 |  |  |
| 5X59 | cryo-EM | 2017 | 3.7 | NTD |  |
| 5W9H | cryo-EM | 2017 | 4.0 |  |  |
| 5W9P | cryo-EM | 2017 | 4.0 |  |  |
| 5X5C | cryo-EM | 2017 | 4.1 |  |  |
| 6PZ8 | cryo-EM | 2019 | 4.2 |  |  |
| 5X5F | cryo-EM | 2017 | 4.2 |  |  |
| 5W9O | cryo-EM | 2017 | 4.5 |  |  |
| 5W9K | cryo-EM | 2017 | 4.6 |  |  |
| 5W9M | cryo-EM | 2017 | 4.7 |  |  |
| 5W9J | cryo-EM | 2017 | 4.8 |  |  |
| 5W9L | cryo-EM | 2017 | 4.8 |  |  |
| 5W9N | cryo-EM | 2017 | 5.0 |  |  |
| **SARS-CoV-1** | | | | | |
| 1ZVA | X-RAY | 2006 | 1.5 |  |  |
| 2BEQ | X-RAY | 2004 | 1.6 |  |  |
| 2BEZ | X-RAY | 2004 | 1.6 |  |  |
| 1ZVB | X-RAY | 2006 | 1.7 |  |  |
| 1ZV8 | X-RAY | 2006 | 1.9 |  |  |
| 1WYY | X-RAY | 2005 | 2.2 |  |  |
| 2GHV | X-RAY | 2006 | 2.2 | RBD |  |
| 5X4S | X-RAY | 2017 | 2.2 | NTD |  |
| 6WAQ | X-RAY | 2020 | 2.2 | RBD |  |
| 2DD8 | X-RAY | 2006 | 2.3 | RBD |  |
| 2GHW | X-RAY | 2006 | 2.3 | RBD |  |
| 7JN5 | X-RAY | 2020 | 2.7 | RBD |  |
| 1WNC | X-RAY | 2004 | 2.8 |  |  |
| 2AJF | X-RAY | 2005 | 2.9 | RBD |  |
| 3D0I | X-RAY | 2008 | 2.9 | RBD | ACE2 |
| 3SCI | X-RAY | 2012 | 2.9 | RBD | ACE2 |
| 3BGF | X-RAY | 2008 | 3.0 | RBD |  |
| 3SCJ | X-RAY | 2012 | 3.0 | RBD | ACE2 |
| 3SCK | X-RAY | 2012 | 3.0 | RBD | ACE2 |
| 3SCL | X-RAY | 2012 | 3.0 | RBD | ACE2 |
| 3D0H | X-RAY | 2008 | 3.1 | RBD | ACE2 |
| 5ZVM | X-RAY | 2019 | 3.3 | HR1 Motif | Pan-CoV’s inhibitor EK1 |
| 6CRV | cryo-EM | 2018 | 3.2 |  |  |
| 6CRZ | cryo-EM | 2018 | 3.3 |  |  |
| 6ACC | cryo-EM | 2018 | 3.6 |  | ACE2 |
| 5XLR | cryo-EM | 2017 | 3.8 |  |  |
| 6CS0 | cryo-EM | 2018 | 3.8 | CTD |  |
| 6ACD | cryo-EM | 2018 | 3.9 |  | ACE2 |
| 6CRW | cryo-EM | 2018 | 3.9 | CTD |  |
| 6CRX | cryo-EM | 2018 | 3.9 | CTD |  |
| 6M3W | cryo-EM | 2020 | 3.9 |  |  |
| 6ACJ | cryo-EM | 2018 | 4.2 |  | ACE2 |
| 6NB6 | cryo-EM | 2019 | 4.2 |  |  |
| 5WRG | cryo-EM | 2017 | 4.3 |  |  |
| 6CS2 | cryo-EM | 2018 | 4.4 |  | ACE2 |
| 6ACK | cryo-EM | 2018 | 4.5 |  | ACE2 |
| 6NB7 | cryo-EM | 2019 | 4.5 |  |  |
| 6CS1 | cryo-EM | 2018 | 4.6 | CTD |  |
| 6ACG | cryo-EM | 2018 | 5.4 |  | ACE2 |
| **SARS-CoV-2** | | | | | |
| 6M1V | X-RAY | 2020 | 1.5 |  |  |
| 6YZ5 | X-RAY | 2020 | 1.8 | RBD |  |
| 6ZBP | X-RAY | 2020 | 1.9 |  |  |
| 6XC4 | X-RAY | 2020 | 2.3 | RBD |  |
| 6YLA | X-RAY | 2020 | 2.4 | RBD |  |
| 6M0J | X-RAY | 2020 | 2.5 | RBD | ACE2 |
| 6LVN | X-RAY | 2020 | 2.5 | HR2 Domain | |
| 6LZG | X-RAY | 2020 | 2.5 | RBD |  |
| 6XKQ | X-RAY | 2020 | 2.6 | RBD |  |
| 6ZCZ | X-RAY | 2020 | 2.7 |  |  |
| 7JX3 | X-RAY | 2020 | 2.7 |  |  |
| 6VW1 | X-RAY | 2020 | 2.7 | RBD | ACE2 |
| 6XC3 | X-RAY | 2020 | 2.7 | RBD |  |
| 6Z2M | X-RAY | 2020 | 2.7 | RBD |  |
| 6XKP | X-RAY | 2020 | 2.7 | RBD |  |
| 6XE1 | X-RAY | 2020 | 2.8 | RBD |  |
| 7C01 | X-RAY | 2020 | 2.9 | RBD |  |
| 6XC7 | X-RAY | 2020 | 2.9 | RBD |  |
| 6LXT | X-RAY | 2020 | 2.9 |  |  |
| 7K9Z | X-RAY | 2020 | 3.0 | RBD |  |
| 6W41 | X-RAY | 2020 | 3.1 | RBD |  |
| 6ZLR | X-RAY | 2020 | 3.1 | RBD |  |
| 6XC2 | X-RAY | 2020 | 3.1 |  |  |
| 7K8M | X-RAY | 2020 | 3.2 | RBD |  |
| 7JVB | X-RAY | 2020 | 3.3 | RBD |  |
| 6YZ7 | X-RAY | 2020 | 3.3 | RBD |  |
| 6ZH9 | X-RAY | 2020 | 3.3 | RBD |  |
| 6ZER | X-RAY | 2020 | 3.8 |  |  |
| 6YM0 | X-RAY | 2020 | 4.4 | RBD |  |
| 6XLU | cryo-EM | 2020 | 2.4 |  |  |
| 7JWY | cryo-EM | 2020 | 2.5 |  |  |
| 6ZGE | cryo-EM | 2020 | 2.6 |  |  |
| 7K43 | cryo-EM | 2020 | 2.6 |  |  |
| 6X29 | cryo-EM | 2020 | 2.7 |  |  |
| 6XM0 | cryo-EM | 2020 | 2.7 |  |  |
| 7DF3 | cryo-EM | 2020 | 2.7 |  | ACE2 |
| 7JZL | cryo-EM | 2020 | 2.7 |  |  |
| 7A4N | cryo-EM | 2020 | 2.8 |  |  |
| 6VXX | cryo-EM | 2020 | 2.8 |  |  |
| 7KDK | cryo-EM | 2020 | 2.8 | RBD |  |
| 6ZB5 | cryo-EM | 2020 | 2.9 |  |  |
| 7KKL | cryo-EM | 2020 | 2.9 |  |  |
| 6M17 | cryo-EM | 2020 | 2.9 | RBD | ACE2 |
| 6X79 | cryo-EM | 2020 | 2.9 |  |  |
| 6XM3 | cryo-EM | 2020 | 2.9 | RBD |  |
| 6XM4 | cryo-EM | 2020 | 2.9 | RBD |  |
| 6XR8 | cryo-EM | 2020 | 2.9 |  |  |
| 6ZGI | cryo-EM | 2020 | 2.9 |  |  |
| 7AD1 | cryo-EM | 2020 | 2.9 |  |  |
| 6ZXN | cryo-EM | 2020 | 2.9 |  |  |
| 7A29 | cryo-EM | 2020 | 2.9 |  |  |
| 7KDL | cryo-EM | 2020 | 3.0 | RBD |  |
| 6XRA | cryo-EM | 2020 | 3.0 |  |  |
| 6ZOW | cryo-EM | 2020 | 3.0 |  |  |
| 6ZOX | cryo-EM | 2020 | 3.0 |  |  |
| 6ZP0 | cryo-EM | 2020 | 3.0 |  |  |
| 7DDD | cryo-EM | 2020 | 3.0 |  |  |
| 7JV6 | cryo-EM | 2020 | 3.0 |  |  |
| 7KDG | cryo-EM | 2020 | 3.0 | RBD |  |
| 6ZB4 | cryo-EM | 2020 | 3.0 |  |  |
| 7KKK | cryo-EM | 2020 | 3.0 |  |  |
| 7A25 | cryo-EM | 2020 | 3.1 |  |  |
| 7KE9 | cryo-EM | 2020 | 3.1 | RBD |  |
| 6WPS | cryo-EM | 2020 | 3.1 |  |  |
| 6XM5 | cryo-EM | 2020 | 3.1 | RBD |  |
| 6ZOY | cryo-EM | 2020 | 3.1 |  |  |
| 6ZP2 | cryo-EM | 2020 | 3.1 |  |  |
| 6ZP5 | cryo-EM | 2020 | 3.1 |  |  |
| 7C2L | cryo-EM | 2020 | 3.1 |  |  |
| 7JZN | cryo-EM | 2020 | 3.1 |  |  |
| 7JZU | cryo-EM | 2020 | 3.1 |  |  |
| 7KE6 | cryo-EM | 2020 | 3.1 | RBD |  |
| 7L09 | cryo-EM | 2020 | 3.1 |  |  |
| 6VYB | cryo-EM | 2020 | 3.2 |  |  |
| 6X2C | cryo-EM | 2020 | 3.2 | RBD |  |
| 7JWB | cryo-EM | 2020 | 3.2 |  |  |
| 7L02 | cryo-EM | 2020 | 3.2 |  |  |
| 6XKL | cryo-EM | 2020 | 3.2 | RBD |  |
| 7KE4 | cryo-EM | 2020 | 3.2 | RBD |  |
| 6X6P | cryo-EM | 2020 | 3.2 |  |  |
| 7K90 | cryo-EM | 2020 | 3.2 |  |  |
| 6XEY | cryo-EM | 2020 | 3.3 |  |  |
| 7KDI | cryo-EM | 2020 | 3.3 | RBD |  |
| 7KE8 | cryo-EM | 2020 | 3.3 | RBD |  |
| 6X2A | cryo-EM | 2020 | 3.3 | RBD |  |
| 6YOR | cryo-EM | 2020 | 3.3 |  |  |
| 6Z43 | cryo-EM | 2020 | 3.3 |  |  |
| 6ZP1 | cryo-EM | 2020 | 3.3 |  |  |
| 6ZP7 | cryo-EM | 2020 | 3.3 |  |  |
| 7K4N | cryo-EM | 2020 | 3.3 |  |  |
| 7L06 | cryo-EM | 2020 | 3.3 |  |  |
| 7KE7 | cryo-EM | 2020 | 3.3 | RBD |  |
| 7KDH | cryo-EM | 2020 | 3.3 | RBD |  |
| 7KEA | cryo-EM | 2020 | 3.3 | RBD |  |
| 7KMB | cryo-EM | 2020 | 3.4 |  |  |
| 6Z97 | cryo-EM | 2020 | 3.4 |  |  |
| 7JV4 | cryo-EM | 2020 | 3.4 |  |  |
| 7K8S | cryo-EM | 2020 | 3.4 |  |  |
| 7K8T | cryo-EM | 2020 | 3.4 |  |  |
| 7KJ4 | cryo-EM | 2020 | 3.4 |  |  |
| 6XCM | cryo-EM | 2020 | 3.4 |  |  |
| 6XF5 | cryo-EM | 2020 | 3.5 | RBD |  |
| 6VSB | cryo-EM | 2020 | 3.5 | RBD |  |
| 7KEB | cryo-EM | 2020 | 3.5 | RBD |  |
| 7KDJ | cryo-EM | 2020 | 3.5 | RBD |  |
| 6ZOZ | cryo-EM | 2020 | 3.5 |  |  |
| 6ZWV | cryo-EM | 2020 | 3.5 | RBD |  |
| 7JV2 | cryo-EM | 2020 | 3.5 |  |  |
| 7JZM | cryo-EM | 2020 | 3.5 |  |  |
| 7K8Z | cryo-EM | 2020 | 3.5 |  |  |
| 6X2B | cryo-EM | 2020 | 3.6 | RBD |  |
| 7A91 | cryo-EM | 2020 | 3.6 |  | ACE2 |
| 7JVA | cryo-EM | 2020 | 3.6 | RBD |  |
| 7KJ2 | cryo-EM | 2020 | 3.6 |  |  |
| 7KJ5 | cryo-EM | 2020 | 3.6 | RBD |  |
| 7KMZ | cryo-EM | 2020 | 3.6 |  | ACE2 |
| 7KMS | cryo-EM | 2020 | 3.6 |  |  |
| 6XCN | cryo-EM | 2020 | 3.7 |  |  |
| 6WPT | cryo-EM | 2020 | 3.7 |  |  |
| 6XS6 | cryo-EM | 2020 | 3.7 |  |  |
| 6ZDH | cryo-EM | 2020 | 3.7 |  |  |
| 7A5R | cryo-EM | 2020 | 3.7 |  |  |
| 7K45 | cryo-EM | 2020 | 3.7 | RBD |  |
| 7KJ3 | cryo-EM | 2020 | 3.7 |  |  |
| 7KNH | cryo-EM | 2020 | 3.7 |  | ACE2 |
| 6ZGG | cryo-EM | 2020 | 3.8 | RBD |  |
| 7DF4 | cryo-EM | 2020 | 3.8 |  |  |
| 7DK4 | cryo-EM | 2020 | 3.8 | RBD |  |
| 7K8U | cryo-EM | 2020 | 3.8 |  |  |
| 7KEC | cryo-EM | 2020 | 3.8 | RBD |  |
| 7KNE | cryo-EM | 2020 | 3.9 |  | ACE2 |
| 6XDG | cryo-EM | 2020 | 3.9 | RBD |  |
| 7A5S | cryo-EM | 2020 | 3.9 |  |  |
| 7A94 | cryo-EM | 2020 | 3.9 |  | ACE2 |
| 7K8X | cryo-EM | 2020 | 3.9 |  |  |
| 7KNI | cryo-EM | 2020 | 3.9 |  | ACE2 |
| 7KNB | cryo-EM | 2020 | 3.9 |  | ACE2 |
| 6XF6 | cryo-EM | 2020 | 4.0 | RBD |  |
| 7KL9 | cryo-EM | 2020 | 4.1 |  |  |
| 7A92 | cryo-EM | 2020 | 4.2 |  | ACE2 |
| 7A95 | cryo-EM | 2020 | 4.3 | RBD | ACE2 |
| 7DCC | cryo-EM | 2020 | 4.3 | RBD |  |
| 7DK6 | cryo-EM | 2020 | 4.3 | RBD |  |
| 6ZFO | cryo-EM | 2020 | 4.4 |  |  |
| 7A97 | cryo-EM | 2020 | 4.4 |  | ACE2 |
| 7K8Y | cryo-EM | 2020 | 4.4 |  |  |
| 6ZDG | cryo-EM | 2020 | 4.7 |  |  |
| 7A96 | cryo-EM | 2020 | 4.8 | RBD | ACE2 |
| 7A98 | cryo-EM | 2020 | 5.4 |  | ACE2 |
| 7DD2 | cryo-EM | 2020 | 5.6 | RBD |  |
| 7A93 | cryo-EM | 2020 | 5.9 | RBD |  |
| 7DCX | cryo-EM | 2020 | 5.9 | RBD |  |
| 7DK3 | cryo-EM | 2020 | 6.0 |  |  |
| 7DDN | cryo-EM | 2020 | 6.3 |  |  |
| 6ZGH | cryo-EM | 2020 | 6.8 |  |  |
| 7DD8 | cryo-EM | 2020 | 7.5 | RBD |  |
| 7DK7 | cryo-EM | 2020 | 9.7 | RBD |  |
| 7DK5 | cryo-EM | 2020 | 13.5 | RBD |  |
